# Supplementary material for: Sequence analysis of sickness absence and disability pension days in 2012–2018 among privately employed white-collar workers in Sweden: a prospective cohort study
Source: BMJ Open. 2023 Dec 13;13(12):e078066. doi: 10.1136/bmjopen-2023-078066 (PMC10729113; doi:10.1136/bmjopen-2023-078066)
Supplement: Supplementary data [file bmjopen-2023-078066supp001.pdf]

Supplementary materials. Salonen, Farrants, Alexanderson. *Sequence analysis of sickness absence and disability pension days in 2012–2018 among privately employed white-collar workers in Sweden: a prospective cohort study*

Supplementary Table 1. The distribution of sociodemographic variables in each cluster of sickness absence (SA) and disability pension (DP) among privately employed white-collar workers

	Cluster 1) low or no SA or DP		Cluster 2) SA due to other diagnoses		Cluster 3) SA due to mental diagnoses		Cluster 4) ineligible for SA and DP		Cluster 5) DP	
	n	%	n	%	n	%	n	%	n	%
**Sex**										
Women	512 131	*45.0*	45 859	*68.4*	32 110	*73.2*	7416	*40.9*	11 277	*71.7*
Men	626 646	*55.0*	21 138	*31.6*	11 761	*26.8*	10 734	*59.1*	4444	*28.3*
**Age group**										
16-24	58 138	*5.1*	3864	*5.8*	2032	*4.6*	924	*5.1*	132	*0.8*
25-34	245 816	*21.6*	16 017	*23.9*	10 857	*24.7*	3891	*21.4*	588	*3.7*
35-44	338 445	*29.7*	16 566	*24.7*	15 502	*35.3*	4008	*22.1*	2700	*17.2*
45-54	287 310	*25.2*	19 226	*28.7*	11 588	*26.4*	3819	*21.0*	5993	*38.1*
55-64	102 487	*9.0*	8736	*13.0*	3326	*7.6*	2358	*13.0*	4030	*25.6*
65-67	106 581	*9.4*	2588	*3.9*	566	*1.3*	3150	*17.4*	2278	*14.5*
**Type of living area**										
Large city	589 869	*51.8*	33 849	*50.5*	22 600	*51.5*	10 778	*59.4*	5548	*35.3*
Medium-sized town	347 373	*30.5*	20 336	*30.4*	13 230	*30.2*	4745	*26.1*	5552	*35.3*
Rural or small town	201 535	*17.7*	12 812	*19.1*	8041	*18.3*	2627	*14.5*	4621	*29.4*
**Educational level**										
Primary	54 084	*4.7*	3689	*5.5*	2253	*5.1*	1442	*7.9*	1681	*10.7*
Secondary	461 868	*40.6*	32 312	*48.2*	20 029	*45.7*	6127	*33.8*	9339	*59.4*
Tertiary	622 825	*54.7*	30 996	*46.3*	21 589	*49.2*	10 581	*58.3*	4701	*29.9*
**Country of birth**										
Sweden	1 023 594	*89.9*	58 529	*87.4*	38 952	*88.8*	13 606	*75.0*	14 079	*89.6*
Other Nordic country	23 540	*2.1*	1622	*2.4*	1009	*2.3*	1219	*6.7*	548	*3.5*
Other EU25 country	22 782	*2.0*	1301	*1.9*	866	*2.0*	1219	*6.7*	269	*1.7*
Other countries	68 861	*6.0*	5545	*8.3*	3044	*6.9*	2106	*11.6*	825	*5.2*
**Family composition**										
Couple without children <18 at home	15 3085	*13.4*	8510	*12.7*	3455	*7.9*	3310	*18.2*	3991	*25.4*
Couple with children <18 at home	543 569	*47.7*	28 512	*42.6*	20 232	*46.1*	6087	*33.5*	5034	*32.0*
Single without children <18 at home	371 865	*32.7*	23 388	*34.9*	14 354	*32.7*	7822	*43.1*	5102	*32.5*

Supplementary materials. Salonen, Farrants, Alexanderson. *Sequence analysis of sickness absence and disability pension days in 2012–2018 among privately employed white-collar workers in Sweden: a prospective cohort study*

Single with children <18 at home	70 258	*6.2*	6587	*9.8*	5830	*13.3*	931	*5.1*	1594	*10.1*
**Sector**										
Manufacturing	242 538	*21.3*	10 409	*15.5*	6237	*14.2*	4000	*22.0*	2068	*13.2*
Service	495 208	*43.5*	25 925	*38.7*	18 436	*42.0*	8090	*44.6*	6738	*42.9*
Trade, hotel, restaurant	146 137	*12.8*	8461	*12.6*	5636	*12.8*	2135	*11.8*	1855	*11.8*
Transport	49 760	*4.4*	3091	*4.6*	1686	*3.8*	822	*4.5*	728	*4.6*
Construction	46 164	*4.1*	2228	*3.3*	1147	*2.6*	534	*2.9*	598	*3.8*
Education, care, nursing, social services	158 460	*13.9*	16 861	*25.2*	10 718	*24.4*	2547	*14.0*	3733	*23.7*
**Income (SEK)**										
7920-87 999	304 420	*26.7*	26 911	*40.2*	19 263	*43.9*	4687	*25.8*	6798	*43.2*
88 000-175 999	20 142	*1.8*	1206	*1.8*	842	*1.9*	1317	*7.3*	810	*5.2*
176 000-329 999	66 039	*5.8*	5248	*7.8*	4159	*9.5*	1393	*7.7*	6430	*40.9*
330 000-439 999	318 059	*27.9*	18 897	*28.2*	11 372	*25.9*	3951	*21.8*	1161	*7.4*
>440 000	430 117	*37.8*	14 735	*22.0*	8235	*18.8*	6802	*37.5*	522	*3.3*
**Number of SA net days in 2012**										
0	1 080 290	*94.9*	53 898	*80.4*	32 433	*73.9*	15 296	*84.3*	9801	*62.3*
1 - 14	20 262	*1.8*	3935	*5.9*	2719	*6.2*	458	*2.5*	1054	*6.7*
15 - 30	12 121	*1.1*	2523	*3.8*	1808	*4.1*	360	*2.0*	602	*3.8*
31 - 90	16 252	*1.4*	3703	*5.5*	3178	*7.2*	682	*3.8*	1233	*7.8*
91 - 180	6145	*0.5*	1723	*2.6*	1828	*4.2*	515	*2.8*	1274	*8.1*
181 - 365	3353	*0.3*	1077	*1.6*	1619	*3.7*	659	*3.6*	1438	*9.1*
366*	352	*0.0*	138	*0.2*	286	*0.7*	180	*1.0*	319	*2.0*
**SA diagnoses in 2012**										
Mental diagnoses	16 509	*1.4*	3111	*4.6*	6460	*14.7*	486	*2.7*	1793	*11.4*
Musculoskeletal diagnoses	12 285	*1.1*	2815	*4.2*	1418	*3.2*	364	*2.0*	1620	*10.3*
Injury	6655	*0.6*	1278	*1.9*	597	*1.4*	205	*1.1*	444	*2.8*
Cancer	3058	*0.3*	718	*1.1*	245	*0.6*	1071	*5.9*	202	*1.3*
Circulatory diagnoses	2627	*0.2*	483	*0.7*	201	*0.5*	150	*0.8*	423	*2.7*
Other diagnoses	14 648	*1.3*	4171	*6.2*	2323	*5.3*	637	*3.5*	1760	*11.2*
**Total**	1 138 777	100.0	66 997	100.0	43 871	100.0	18 150	100.0	15 721	100.0

* 2012 was a leap year, thus those indviduals were on SA for full-time all year.

Supplementary materials. Salonen, Farrants, Alexanderson. *Sequence analysis of sickness absence and disability pension days in 2012–2018 among privately employed white-collar workers in Sweden: a prospective cohort study*

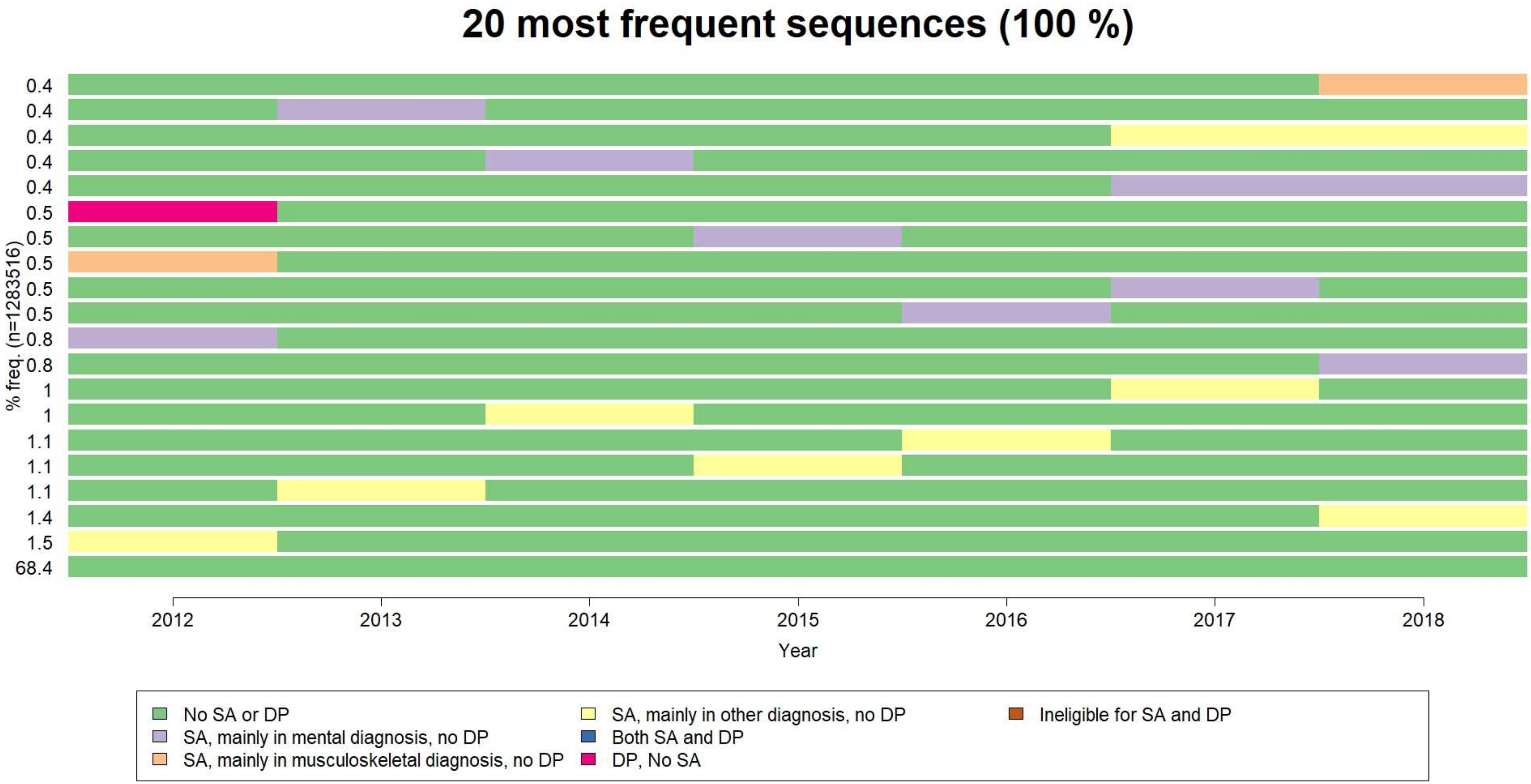

Supplementary Figure 1 Frequency plot for the 20 most frequent sequences of sickness absence (SA) and disability pension (DP) over 7 years among privately employed white-collar workers in Sweden
