# Supplementary material for: Sequence analysis of sickness absence and disability pension days in 2012–2018 among privately employed white-collar workers in Sweden: a prospective cohort study
Source: BMJ Open. 2023 Dec 13;13(12):e078066. doi: 10.1136/bmjopen-2023-078066 (PMC10729113; doi:10.1136/bmjopen-2023-078066)
Supplement: Author's manuscript [file bmjopen-2023-078066.draft_revisions.pdf]

BMJ Open is committed to open peer review. As part of this commitment we make the peer review history of every article we publish publicly available.

When an article is published we post the peer reviewers' comments and the authors' responses online. We also post the versions of the paper that were used during peer review. These are the versions that the peer review comments apply to.

The versions of the paper that follow are the versions that were submitted during the peer review process. They are not the versions of record or the final published versions. They should not be cited or distributed as the published version of this manuscript.

BMJ Open is an open access journal and the full, final, typeset and author-corrected version of record of the manuscript is available on our site with no access controls, subscription charges or pay-per-view fees (http://bmjopen.bmj.com).

If you have any questions on BMJ Open's open peer review process please email
info.bmjopen@bmj.com

BMJ Open

Sequence analysis of sickness absence and disability pension days in 2012–2018 among privately employed white-collar workers in Sweden: a prospective cohort study

Journal:	*BMJ Open*
Manuscript ID	bmjopen-2023-078066
Article Type:	Original research
Date Submitted by the Author:	23-Jul-2023

Complete List of Authors:	Salonen, Laura; Finnish Institute of Occupational Health<br>Alexanderson, Kristina; Karolinska Institutet, Clinical Neuroscience<br>Farrants, Kristin ; Karolinska Institute Division of Insurance Medicine,

Keywords:	EPIDEMIOLOGIC STUDIES, EPIDEMIOLOGY, OCCUPATIONAL & INDUSTRIAL MEDICINE, PUBLIC HEALTH, REGISTRIES

SCHOLARONE™
Manuscripts

I, the Submitting Author has the right to grant and does grant on behalf of all authors of the Work (as defined in the below author licence), an exclusive licence and/or a non-exclusive licence for contributions from authors who are: i) UK Crown employees; ii) where BMJ has agreed a CC-BY licence shall apply, and/or iii) in accordance with the terms applicable for US Federal Government officers or employees acting as part of their official duties; on a worldwide, perpetual, irrevocable, royalty-free basis to BMJ Publishing Group Ltd ("BMJ") its licensees and where the relevant Journal is co-owned by BMJ to the co-owners of the Journal, to publish the Work in this journal and any other BMJ products and to exploit all rights, as set out in our licence.

The Submitting Author accepts and understands that any supply made under these terms is made by BMJ to the Submitting Author unless you are acting as an employee on behalf of your employer or a postgraduate student of an affiliated institution which is paying any applicable article publishing charge ("APC") for Open Access articles. Where the Submitting Author wishes to make the Work available on an Open Access basis (and intends to pay the relevant APC), the terms of reuse of such Open Access shall be governed by a Creative Commons licence – details of these licences and which Creative Commons licence will apply to this Work are set out in our licence referred to above.

Other than as permitted in any relevant BMJ Author's Self Archiving Policies, I confirm this Work has not been accepted for publication elsewhere, is not being considered for publication elsewhere and does not duplicate material already published. I confirm all authors consent to publication of this Work and authorise the granting of this licence.

For peer review only

Sequence analysis of sickness absence and disability pension days in 2012–2018 among privately employed white-collar workers in Sweden: a prospective cohort study

Laura Salonen[1], Kristina Alexanderson[2] & Kristin Farrants[2]

1 Finnish Institute of Occupational Health, Finland

2 Division of Insurance Medicine, Department of Clinical Neuroscience, Karolinska Institutet, Sweden

For peer review only

For peer review only - http://bmjopen.bmj.com/site/about/guidelines.xhtml

ABSTRACT

Objective: To explore sequences of sickness absence (SA) and disability pension (DP) days from 2012 through 2018 among privately employed white-collar workers.

Methods: A seven-year prospective cohort study of all 1,283,516 privately employed white-collar workers in Sweden in 2012 aged 16–67. Microdata from nationwide registers were used for sequence analysis to describe clusters of individuals who followed similar development of SA and DP net days/year, and multinomial logistic regression to analyze associations between sociodemographic variables and belonging to each observed cluster of sequences. Odds ratios (OR) and 95% confidence intervals (CI) were adjusted for baseline sociodemographics.

Results: We identified five clusters of SA and DP sequences: 1) 'low or no SA or DP' (88.7% of the population), 2) 'SA due to other than mental diagnosis' (5.2%), 3) 'SA due to mental diagnosis' (3.4%), 4) 'not eligible for SA or DP' (1.4%), and 5) 'DP' (1.2%). Men, highly educated, born outside Sweden, and high-income earners were more likely to belong to the first and the fourth cluster (ORs range 1.13-4.49). The second, third, and fifth clusters consisted mainly of women, low educated, and low-income (ORs range 1.22-8.90). There were only small differences between branches of industry in adjusted analyses, and many were not significant.

Conclusion: In general, only a few privately employed white-collar workers had SA and even fewer had DP during the seven-year follow-up. The risk of belonging to a cluster characterized by SA or DP varied by sex, levels of education and income, and other sociodemographic factors.

Keywords: sick leave, sequence analysis, private sector

Strengths and limitations of this study:

- Detailed sociodemographic microdata, linked from different population-based registers, about a cohort of all privately employed white-collar workers in Sweden in 2012
- Long study period (7 years) with no loss to follow-up and no bias from self-reports

For peer review only - http://bmjopen.bmj.com/site/about/guidelines.xhtml

- Use of sequence analysis to capture the heterogeneity of the different sickness absence and disability pension patterns over time
- How many and which states to include in the analyses is dependent on researcher judgement and thus can be arbitrary

For peer review only

For peer review only - http://bmjopen.bmj.com/site/about/guidelines.xhtml

KEY MESSAGES

What is already known about this subject?

- Sickness absence and disability pension rates are often very low among privately employed white-collar employees. However, there are differences in the prevalence of sickness absence and disability pension within this group, related to factors such as sociodemographic factors, the branch of industry, or occupation.

What are the new findings?

- We identified five clusters of individuals who followed similar sequences over a seven-year period: 1) 'low or no sickness absence or disability pension' (88.7% of the population), 2) 'sickness absence due to other than mental diagnosis' (5.2%), 3) 'sickness absence due to mental diagnosis' (3.4%), 4) 'not eligible for sickness absence or disability pension' (1.4%), and 5) 'disability pension' (1.2%)
- Female sex, low education, and working in education, care, nursing, or social work industry are factors associated with a higher risk of future sickness absence or disability pension, especially due to mental diagnoses
- While the previously mentioned characteristics usually explain occupational class differences in sickness absence and or disability pension, a novel finding was that they also explained the differences among privately employed white-collar workers

How might this impact on policy or clinical practice in the foreseeable future?

- Recognizing sequence groups that are associated with a higher risk of future sickness absence or disability pension can help to plan preventive measures at an early phase; this exploratory study provides information on factors that need to be further studied.

For peer review only - http://bmjopen.bmj.com/site/about/guidelines.xhtml

INTRODUCTION

Sickness absence (SA) and disability pension (DP) have adverse consequences for individuals, their employers, and welfare states. The development of a SA and DP is often a long process and varies with the type of occupation and work tasks (1,2). In general, white-collar workers have a lower risk of SA and DP compared to other occupational groups (3–5). However, they constitute a large part of the workforce – approximately half in Sweden in 2018 (6). Thus, work incapacity in this group can impose high costs for employees, employers, and the welfare state. To prevent work incapacity in this population, more knowledge is needed on the determinants and the process of developing a long-term work incapacity.

Previous research on SA and DP among specific occupations or occupational groups have mainly focused on so-called high-risk groups, i.e., manual workers and blue-collar workers (7–13), while studies on white-collar workers are scarce. Those conducted are mainly based on small sample sizes (14,15). To the best of our knowledge, only four large-scale studies have been conducted on SA and DP among white-collar employees; the Whitehall-II studies of British civil servants (16,17), a Swedish cross-sectional study on privately employed white-collar workers (3) and a Swedish study on privately employed white-collar workers in the trade and retail industry (18). The results of the two latter studies showed that the risk of SA and DP – and the risk of belonging to an adverse SA/DP trajectory – differed among white-collar workers by age, sex, education, a branch of industry, psychosocial exposures at work, and other sociodemographic factors. More studies on full population data with a longitudinal research design are needed to increase the knowledge base.

Studies on white-collar workers in the *private* sector are even more limited. In general, large-scale studies have demonstrated that SA rates in the private sector are generally lower than in the public sector (19,20). Only a few studies exist on SA and/or DP specifically among private-sector employees, but not among white-collar workers, and they are mainly conducted on small, selected populations, with a large drop-out rate, and mainly based on self-reported data (5,21–23). Three large-scale studies on private sector white-collar employees have been published so far: the previously mentioned Swedish studies (3,18) and a Greek study on private sector employees that found a smaller SA rate in the shipyard industry than in other industries (24). Further, none of these studies have accounted for transitions between other labour market states in addition to SA and DP, such as employment and unemployment.

Sequence analysis is a good method to study development over time. Unlike more traditionally used methods, such as event history analysis or growth curve models, sequence analysis can describe the duration and frequency of multiple categorical statuses. This holistic perspective is essential in providing an overview of the development of SA and DP days, and in identifying potential sub-groups within a population who share particular patterns in terms of these SA and DP days.

For peer review only - http://bmjopen.bmj.com/site/about/guidelines.xhtml

The aim of this study was to identify sequences of white-collar workers in the private sector who follow future similar sequences of SA and DP days/year and second, to analyze the sociodemographic and diagnostic composition of the observed clusters of SA and DP.

METHODS

Data sources and population

We conducted a seven-year prospective population-based cohort study. We used the following three nationwide Swedish administrative registers linked at the individual level by personal identity number (PIN; a unique 10-digit number assigned to all Swedish residents) (25):

-The Longitudinal Integration Database for Health Insurance and Labour Market Studies (LISA) held by Statistics Sweden to identify the study cohort and for information on socio-demographic characteristics for baseline 2012 and regarding being in paid work or not in 2012-2018 (see Variables below) or emigrating in 2013-2018.

-The MicroData for Analysis of the Social Insurance database (MiDAS) held by the Swedish Social Insurance Agency for information on SA and DP in the years 2012–2018 regarding SA and DP (dates, grades (full- or part-time) and diagnoses), and

-The Cause of Death Register held by the National Board of Health and Welfare for year of death.

The study population consisted of all individuals aged 18–67 years who lived in Sweden on both 31 December 2011 and 31 December 2012, who had an occupational code according to the Swedish Standard for Occupational Classification indicating a white-collar occupation and who were employed at a private-sector company during 2012 and had an income from work, parental benefits, SA, and/or DP that amounted to at least 75% of the necessary income level to qualify for SA benefits from the Social Insurance Agency (7920 Swedish krona (SEK) in 2012, approx. €910 by the 2012 exchange rate, updated yearly in line with inflation). We excluded unemployed, self-employed persons, and those who were on DP full-time for the entire year 2012 (n=461). The total study cohort included 1,283,516 individuals.

Public sickness absence insurance in Sweden

In Sweden, all residents aged at least 16 years with an income from work or unemployment benefits who have a reduced work capacity due to morbidity are covered by the national public SA insurance (26). A physician's certificate is required after seven days. After an unpaid qualifying day, the employer pays the first 14 SA days, after which SA benefits are paid by the Social Insurance Agency. For the unemployed, the Social Insurance Agency pays after the first qualifying day. Thus, we excluded SA spells shorter than 15 days, in order not to introduce bias, since we only had information of SA spells exceeding 14 days for the employed. There was no limitation regarding how long a SA spell could be ongoing for. Residents in Sweden aged 19–64 years whose work capacity is reduced

For peer review only - http://bmjopen.bmj.com/site/about/guidelines.xhtml

permanently or for a long-term period can be granted DP from the Social Insurance Agency. SA covers about 80% of lost income, DP about 65% of lost income, both up to a certain level. Both SA and DP can be granted for part- or full-time (25%, 50%, 75%, or 100% of ordinary work hours). This means that people can be on partial SA and DP at the same time.

Sociodemographic and work-related variables

We included information on sex, age group, country of birth, educational level, family composition, type of living area, and branch of industry based on the Swedish Standard for Industry Classification (SNI) categorized into the following six groups: manufacturing, services, transport, construction and installation, care and education, or commerce and hospitality. All variables were measured at the baseline year 2012.

Measures on sickness absence and disability pension

We used SA net days/year and DP net days/year as outcomes. Net days were calculated so that partial days of SA or DP were combined, e.g., two days of part-time SA for 50% were summed to one net day, and a similar procedure was used for DP days. The first 14 days in SA spells (>14 days) were counted as being of the same grade as day 15 for the purpose of calculating net days during these first 14 days. The number of SA net days in 2012 were categorized as shown in Table 1. The SA diagnoses were categorized into the following seven International Classification of Diseases (ICD-10) groups (27): Cancer (C00-D48), Mental diagnoses (F00-F99 and Z73), Circulatory diseases (I00-I99), Musculoskeletal diagnoses (M00-M99), Pregnancy-related diagnoses (O00-O99), Injuries (S00-T98), and other diagnostic groups (all others, including missing diagnosis (approximately 1% of all spells). In the sequence analyses, pregnancy-related diagnoses were dropped, as no men could have pregnancy-related diagnoses, which made it highly correlated with sex.

In analyses of the yearly states of SA/DP, all diagnoses other than mental disorders and musculoskeletal diseases were combined to form one status. Any DP, regardless of diagnosis, was considered as one group.

Sequence analysis and multinomial regression analysis

We used sequence analysis to examine different statuses of SA and DP days/year, and the transitions between such statuses. SA and DP status was measured on a yearly basis for each of the seven follow-up years and was coded into one of the following seven statuses:

1) no SA or DP,
2) SA due to mental diagnosis but no DP,
3) SA due to musculoskeletal diseases but no DP,
4) SA due to other diagnoses but no DP,

For peer review only - http://bmjopen.bmj.com/site/about/guidelines.xhtml

5) both SA and DP,

6) only DP, and

7) ineligible for SA and DP (emigrated, dead, or no qualifying income from work or related benefits).

Individuals who had SA in more than one diagnostic category were assigned to the diagnostic category they had the most days in that year. We illustrated the individual and proportional changes in SA/DP statuses over time with sequence index plots and status proportion plots.

We used optimal matching (OM) method to group similar sequences with each other. OM measures the dissimilarities through the changes needed to make two sequences identical (28), in other words, the OM algorithm creates metric distances between two sequences, which can be defined as the minimum combination of replacements, insertion and deletions to transform one sequence to another (29). We used R statistical program version 4.1.0 and packages TraMineR and nnet for the sequence analysis.

We used multinomial regression analysis to analyze how sociodemographic characteristics and job industry were associated with each of the obtained clusters, using the first cluster as the reference category. Odds ratios (ORs) with their 95% confidence intervals were reported.

Patient and public involvement

Representatives from the private white-collar sector in Sweden, both for employees and employers (the labour union PTK, the Confederation of Swedish Enterprise, and Alecta) were involved in selecting the research questions through joint meetings throughout the project period, and afterwards in disseminating results.

RESULTS

Characteristics of the study population

Table 1 shows the characteristics of the study cohort of the 1,283,516 privately employed white-collar workers. There were slightly more men (52.4%) in the cohort. The largest age group was those aged 35-44 years (29.5%), over half lived in a large city (51.5%) and had a tertiary education (53.7%). The majority were born in Sweden (89.7%), and almost half were married or cohabiting and having children below the age of 18 at home (47.3%). The largest group was the service industry (43.1%) and the largest income group was those who earned over 440,000 SEK (around 50,556 EUR according to the average 2012 conversion rate) per year (35.8%). A large majority did not have any SA during the follow-up: only around 7% had at least some SA spell >14 days. During the first year of the study, in

For peer review only - http://bmjopen.bmj.com/site/about/guidelines.xhtml

For peer review only

2012, around 2.2% had SA due to mental diagnoses, 1.4% due to musculoskeletal diagnoses and around 3.8% due to any other diagnoses.

[Table 1. Characteristics of the study population in 2012.]

Clusters of sickness absence and disability pension trajectories

We identified five different groups of sequences, i.e., clusters (Figure 1). The sociodemographic characteristics of each cluster can be seen in Supplementary Table 1. The first cluster (n=1,138,777, 88.7% of all in the cohort) was the largest one, and almost 95% of individuals in this cluster had no SA or DP days. We called this cluster 'low or no SA or DP'.

Cluster 2 (n=66 997, 5.2%), which was the second largest, was characterized by SA due other than mental diagnosis, including those with mainly musculoskeletal diagnoses (Figure 1). We called this cluster 'SA due to other diagnoses'.

Cluster 3 (n=43 871, 3.4%) consisted mostly of those who had SA mainly due to mental diagnoses (Figure 1). We called this cluster 'SA due to mental diagnoses'.

Cluster 4 (n=18 150, 1.4%) was characterized by individuals who were not eligible for SA or DP since they either died, emigrated, or left the labor force (Figure 1). We called this cluster 'ineligible for SA and DP'.

The smallest cluster, Cluster 5 (n=15 721, 1.2%) was characterized by individuals who had either partial or full-time DP (Figure 1). We called this cluster 'DP'.

To better understand the most common SA and DP sequences, we examined the 20 most frequent sequences (Supplementary Figure 1). Most (68.4%) had no SA or DP during the follow-up. The remaining trajectories largely consisted of sequences where individuals had SA for one year and then returned to no SA or DP. Very few had DP during the follow-up.

For peer review only - http://bmjopen.bmj.com/site/about/guidelines.xhtml

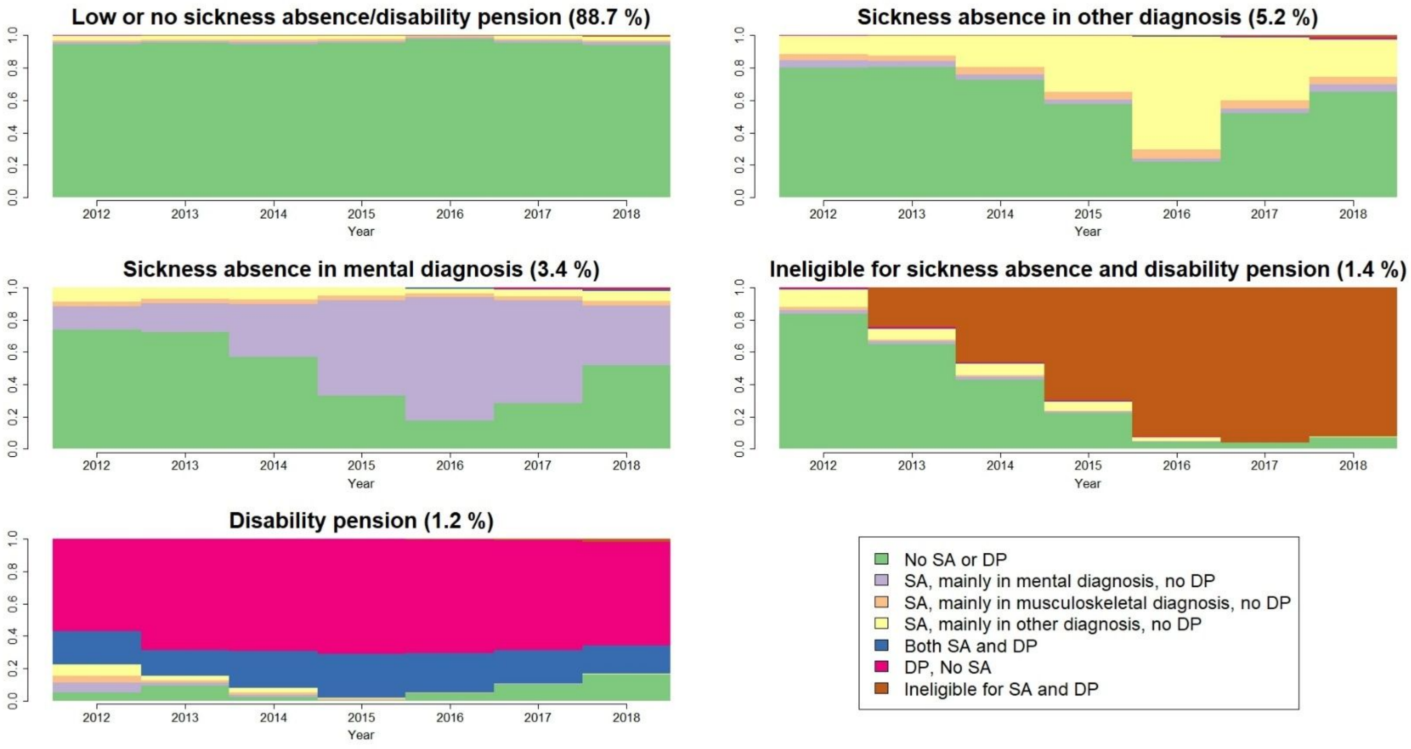

[Figure 1: density plot of sickness absence and disability pension visualizing the proportion of each sickness absence and disability pension status for each cluster over the follow-up]

The associations between individual characteristics and belonging to clusters of sickness absence and disability pension

To study how individual characteristics and SA at baseline were associated with cluster membership, we used multinomial regression analysis. Cluster 1 ‘low or no SA or DP’ was used as the reference category since it was the largest and most homogenous in its sequence content (Table 2). Cluster 1 could be described as consisting of men of younger working-age, who had high levels of education and income, worked in service industry or in manufacturing and had no or only little SA in 2012 (Supplementary Table 1).

In the fully adjusted models, compared to cluster 1 ‘low or no SA or DP’, women (men having an OR of 0.47 (95% CI 0.46-0.47)), over or under 35-44 year old (but not over 64 year old), those with less than tertiary education, who were born outside EU25 countries, living with children, worked in service industry or education, care, nursing, or social service industry, had a medium income, had any SA and especially SA due to cancer or due to ‘other diagnoses’ were more likely to belong to cluster 2 ‘SA due to mental diagnoses’ (Table 2).

For peer review only - http://bmjopen.bmj.com/site/about/guidelines.xhtml

Women (men having an OR of 0.38 (95% CI 0.37-0.39)), 34-44 years old, who had less than tertiary education, who were single living with children, worked in education, care, nursing, or social service industry, had medium low income, had more than 188 SA days in 2012, especially due to mental diagnoses had the highest ORs of belonging to cluster 3 'SA due to mental diagnoses' (Table 2). The second and third clusters could be described as consisting of working-age women, who had less than tertiary education and medium income, who worked in education, care, nursing, or social service industry and had some SA in baseline year, especially due to mental diagnoses in the third cluster (Supplementary Table 1).

The OR for belonging to cluster 4 'ineligible for SA and DP' was the highest in men (OR 1.13; 95% CI 1.10-1.17), 65-67 years old, had primary education, lived without children, were born outside Sweden, had a very low income, who worked in trade, hotel, or restaurant industry or transport industry, had >180 SA days in 2012 and had SA due to circulatory diagnoses (Table 2). The fourth cluster could be described as consisting of men over 64 years old, who had primary education and were born outside Sweden, had low income, and had long-term SA in 2012, especially due to cancer (Supplementary Table 1).

The OR of belonging to cluster 5 'DP' were higher in women (OR 0.69 (95% CI 0.66-0.72) in men), 45-64 years old, who had less than tertiary education, were born in Sweden, who were single, worked in manufacturing, had low to medium low income, had at least 30 SA days in 2012 and especially those with SA due to circulatory diagnoses (Table 2). This fifth cluster could be described as consisting of older working age women, with low education, working in service industry with low income and long-term SA at baseline (Supplementary Table 1).

[Table 2: associations between sociodemographic factors and work disability clusters]

DISCUSSION

In this large prospective cohort study of all 1.2 million privately employed white-collar workers in Sweden in 2012, we analyzed the development of their future number of SA and DP days/year up through 2018. In general, most of the employees had no SA during the follow-up and DP was even rarer. We found five clusters of future SA and DP trajectories: 1) 'low or no SA or DP' (88.7% of all), 2) 'SA due to other (than mental) diagnosis' (5.2%), 3) 'SA due to mental diagnosis' (3.4%), 4) 'not eligible for SA or DP' (1.4%), and 5) 'DP' (1.2%). These results suggest that the majority of privately employed white-collar workers are doing well in terms of SA/DP.

We found some differences related to sociodemographic factors in terms of belonging to different sequence clusters. Many of those in cluster 1 'low or no SA or DP' were Swedish-born, 25–54-years

For peer review only - http://bmjopen.bmj.com/site/about/guidelines.xhtml

old, highly educated, and high-income earning men, who lived in a large city, and were married or cohabiting and having children at home. The same sociodemographic characteristics are typically associated with lower risk of SA or DP in longitudinal nationwide studies (30,31).

We also found that female sex, low education, low income, and working in education, care, nursing, or social services were associated with a higher risk of belonging to clusters characterized by at least some SA or DP. Similar results were found in a previous cross-sectional study using the same data with number and prevalence of SA days as outcomes (3), as well as studies on SA and DP among white-collar workers in the trade and retail industry (18,32). In general, previous longitudinal population-based studies have consistently found that women, low educated, and low-and income earners (30,31) and those working in health care and service industry (20) have a higher risk of SA and/or DP. While these characteristics – low education, low income, and working in the healthcare industry - are usually considered as explanations to why blue-collar workers have a higher risk of SA or DP than white-collar workers (4,33), our results indicate that the same risk factors apply within white-collar employees working in the private sector.

It is understandable that SA due to mental diagnoses constituted an independent cluster since among white-collar workers that is the most common specific diagnostic group of SA and/or DP (1,32,34–36). This cluster was more common among women, 34-44 years olds less than tertiary educated, low-income earners who worked in education, care, nursing and social industry, and had a long SA spell in 2012, which are known risk factors for SA due to mental diagnoses in general (37,38).

The cluster 'ineligible for SA or DP' had relatively many individuals aged ≥55 years, which makes sense since those who left paid work (e.g., through old-age pension) or died during the follow-up belonged to this cluster. There were also many highly educated and high-income earners, who typically are occupationally and geographically mobile, in this cluster. Relatively many of them were born outside Sweden; many of them probably emigrated from Sweden. Those who had SA due to cancer in 2012, had higher OR of belonging to this cluster than to any other cluster.

We found that the estimates for associations between branch of industry and cluster attenuated in the adjusted analyses, indicating that differences between the various branches of industry were more related to other factors. The Swedish Social Insurance Agency has found that in Sweden, occupation is more closely associated with SA than branch of industry (39). However, to what extent this is true within the group white-collar workers is unknown and should be further studied.

Strength and limitations

Strengths of this study are the use of a large, population-based cohort, that linked microdata from three high-quality nationwide registers without dropouts, the long follow-up, and that all data were

For peer review only - http://bmjopen.bmj.com/site/about/guidelines.xhtml

administrative, not self-reports with possible bias. Using sequence analysis allowed us to explore specific sub-groups in the development of SA and DP. Other strengths are that all included were covered by the public SA and DP insurances, and the high employment-frequency in Sweden, that is, the healthy-worker effect did not bias the result much.

Since the study population consisted of privately employed white-collar workers in Sweden, the results cannot directly be generalized to other types of occupational populations or to other countries with other SA/DP systems or employment frequencies. Future studies might choose to explore other, or more specific SA states, regarding number of SA days or part- and full time SA/DP. As this was an observational study, no causal inferences can be drawn from the results.

CONCLUSION

In general, privately employed white-collar workers rarely had SA and even more rarely DP days during the seven-year follow-up. The risk of belonging to a cluster characterized by receiving SA varied by sex, levels of education and income, job industry, and other sociodemographic factors.

Funding: The study was funded by Alecta Insurance. We utilized data from the REWHARD consortium supported by the Swedish Research Council (grant no. 2017-00624).

Data availability statement: The used data cannot be made publicly available due to privacy regulations. According to the General Data Protection Regulation, the Swedish law SFS 2018:218, the Swedish Data Protection Act, the Swedish Ethical Review Act, and the Public Access to Information and Secrecy Act, these types of sensitive data can only be made available for specific purposes that meets the criteria for access to this type of sensitive and confidential data as determined by a legal review. Professor Kristina Alexanderson (Kristina.alexanderson@ki.se) can be contacted regarding the data.

Ethics statements: The project was approved by the Regional Ethical Review Board in Stockholm, reference numbers 2009/1917-32, 2016/1533-32. In this observational study, based on population-based de-identified register data, informed consent was not applicable. All methods were performed in accordance with the relevant guidelines and regulations.

Patient consent for publication: Not applicable.

Acknowledgements: Not applicable.

Competing Interest: None declared.

For peer review only - http://bmjopen.bmj.com/site/about/guidelines.xhtml

REFERENCES

1. Helgadóttir B, Narusyte J, Ropponen A, et al. The role of occupational class on the association between sickness absence and disability pension: A Swedish register-based twin study. Scand J Work Environ Health. 2019; 45(6):622-630. doi: 10.5271/sjweh.3816.

2. Salonen L, Blomgren J, Laaksonen M, et al. Sickness absence as a predictor of disability retirement in different occupational classes: a register-based study of a working-age cohort in Finland in 2007–2014. BMJ Open. 2018;8(5):e020491. doi: 10.1136/bmjopen-2017-020491.

3. Farrants K, Alexanderson K. Sickness absence among privately employed white-collar workers: A total population study in Sweden. Scand J Public Health. 2021;49(2):159–67. doi: 10.1177/1403494820934275.

4. Piha K, Laaksonen M, Martikainen P, et al. Interrelationships between education, occupational class, income and sickness absence. Eur J Public Health. 2010;20(3):276–80. doi: 10.1093/eurpub/ckp162.

5. Väänänen A, Kalimo R, Toppinen-Tanner S, et al. Role clarity, fairness, and organizational climate as predictors of sickness absence: A prospective study in the private sector. Scand J Public Health. 2004;32(6):426–34. doi: 10.1080/14034940410028136.

6. Statistics Sweden. Yrkesregistret med yrkesstatistik 2018: Yrkesstrukturen I Sverige [The Swedish Occupational Register with statistics 2018: The occupational structure in Sweden]. Stockholm, Sweden: Statistics Sweden; 2020.

7. Ahola K, Gould R, Virtanen M, et al. Occupational burnout as a predictor of disability pension: a population-based cohort study. Occup Environ Med. 2009;66(5):284–90. doi: 10.1136/oem.2008.038935.

8. Alexopoulos EC. Prognostic factors for respiratory sickness absence and return to work among blue collar workers and office personnel. Occup Environ Med. 2001;58(4):246–52. doi: 10.1136/oem.58.4.246.

9. Andersen LL, Fallentin N, Thorsen SV, et al. Physical workload and risk of long-term sickness absence in the general working population and among blue-collar workers: prospective cohort study with register follow-up. Occup Environ Med. 2016;73(4):246–53. doi: 10.1093/eurpub/cky073.

10. Arndt V. Construction work and risk of occupational disability: a ten year follow up of 14 474 male workers. Occup Environ Med. 2005;62(8):559–66. doi: 10.1136/oem.2004.018135.

11. Haukka E, Kaila-Kangas L, Luukkonen R, et al. Predictors of sickness absence related to musculoskeletal pain: a two-year follow-up study of workers in municipal kitchens. Scand J Work Environ Health. 2014;40(3):278–86. doi: 10.5271/sjweh.3415.

12. Järvholm B, Stattin M, Robroek SJ, et al. Heavy work and disability pension – a long term follow-up of Swedish construction workers. Scand J Work Environ Health. 2014;40(4):335–42. doi: 10.5271/sjweh.3413.

13. Rantonen O, Alexanderson K, Pentti J, et al. Trends in work disability with mental diagnoses among social workers in Finland and Sweden in 2005–2012. Epidemiology and Psychiatric Sciences. 2016;1–11. doi: 10.1017/S2045796016000597.

For peer review only - http://bmjopen.bmj.com/site/about/guidelines.xhtml

14. Krantz G, Lundberg U. Workload, work stress, and sickness absence in Swedish male and female white-collar employees. Scand J Soc Med. 2006;34(3):238–46.

15. Roelen CAM, Heymans MW, van Rhenen W, et al. Fatigue as Prognostic Risk Marker of Mental Sickness Absence in White Collar Employees. J Occup Rehabil. 2013.

16. Feeney A, North F, Head J, et al. Socioeconomic and sex differentials in reason for sickness absence from the Whitehall II Study. Occupational and Environmental Medicine. 1998;55(2):91–8. doi: 10.1136/oem.55.2.91.

17. Head J, Ferrie JE, Alexanderson K, et al. Diagnosis-specific sickness absence as a predictor of mortality: the Whitehall II prospective cohort study. BMJ. 2008;337(oct02 2):a1469–a1469. doi: 10.1136/bmj.a1469.

18. Farrants K, Alexanderson K. Trajectories of sickness absence and disability pension days among 189,321 white-collar workers in the trade and retail industry; a 7-year longitudinal Swedish cohort study. BMC Public Health. 2022;22(1):1592. doi: 10.1186/s12889-022-14005-y.

19. Björkenstam E, Helgesson M, Gustafsson K, et al. Sickness absence due to common mental disorders in young employees in Sweden: are there differences in occupational class and employment sector? Soc Psychiatry Psychiatr Epidemiol. 2021;57(5):1097-1106. doi: 10.1007/s00127-021-02152-3

20. Lund T, Labriola M, Villadsen E. Who is at risk for long-term sickness absence? A prospective cohort study of Danish employees. Work. 2007;28(3):225–30.

21. Koopmans PC, Roelen CAM, Groothoff JW. Frequent and long-term absence as a risk factor for work disability and job termination among employees in the private sector. Occup Environ Med. 2008;65(7):494–9. doi: 10.1136/oem.2007.034322.

22. Lund T, Kivimaki M, Labriola M, et al. Using administrative sickness absence data as a marker of future disability pension: the prospective DREAM study of Danish private sector employees. Occup Environ Med. 2008;65(1):28–31. 10.1136/oem.2006.031393.

23. Väänänen A, Toppinen-Tanner S, Kalimo R, et al. Job characteristics, physical and psychological symptoms, and social support as antecedents of sickness absence among men and women in the private industrial sector. Soc Sci & Med. 2003;57(5):807–24. doi: 10.1016/S0277-9536(02)00450-1.

24. Alexopoulos EC, Merekoulias G, Tanagra D, et al. Sickness Absence in the Private Sector of Greece: Comparing Shipyard Industry and National Insurance Data. IJERPH. 2012;9(4):1171–81. doi: 10.3390/ijerph9041171.

25. Ludvigsson JF, Otterblad-Olausson P, Pettersson BU, et al. The Swedish personal identity number: possibilities and pitfalls in healthcare and medical research. Eur J Epidemiol. 2009;24(11):659–67. doi: 10.1007/s10654-009-9350-y.

26. Swedish Social Insurance Agency. Social Insurance in Figures 2021. Stockholm, Sweden; 2021. https://statistik.forsakringskassan.se/wps/wcm/connect/11bc72d6-4bbb-4893-8a3b-c9e9eae568f8/social-insurance-in-figures-2021.pdf?MOD=AJPERES&CVID= (accessed 15 Jun 2023).

27. WHO WHO. International Classification of Diseases: Tenth Revision, ICD-10. 2010. http://apps.who.int/classifications/icd10/browse/2010/en. (accessed 15 Jun 2023).

For peer review only - http://bmjopen.bmj.com/site/about/guidelines.xhtml

28. Abbott A, Forrest J. Optimal Matching Methods for Historical Sequences. J Interdiscip. 1986;16(3):471–94.

29. Abbott A, Tsay A. Sequence Analysis and Optimal Matching Methods in Sociology: Review and Prospect. Sociol Methods Res. 2000;29(1):3–33. doi: 10.1177/0049124100029001001.

30. Allebeck P, Mastekaasa A, Swedish Council on Technology Assessment in Health Care (SBU). Chapter 5. Risk factors for sick leave - general studies. Scand J Public Health. 2004 Oct;32(63_suppl):49–108. doi: 10.1080/14034950410021853.

31. Beemsterboer W, Stewart R, Groothoff J, et al. A literature review on sick leave determinants (1984-2004). Int J Occup. 2009;22(2). doi: 10.2478/v10001-009-0013-8.

32. Farrants K, Alexanderson K. Sickness Absence and Disability Pension in the Trade and Retail Industry: A Prospective Cohort Study of 192,000 White-Collar Workers in Sweden. J Occup Environ Med. 2022;64(11):912–9. doi: 10.1097/JOM.0000000000002634.

33. Sumanen H, Pietiläinen O, Lahti J, et al. Interrelationships between education, occupational class and income as determinants of sickness absence among young employees in 2002–2007 and 2008–2013. BMC Public Health. 2015;15(1):332. doi: 10.1186/s12889-015-1718-1.

34. Karolaakso T, Autio R, Näppilä T, et al. Socioeconomic factors in disability retirement due to mental disorders in Finland. Eur J Public Health. 2020;30(6):1218–24. doi: 10.1093/eurpub/ckaa132.

35. Leinonen T, Pietiläinen O, Laaksonen M, et al. Occupational social class and disability retirement among municipal employees – the contribution of health behaviors and working conditions. Scand J Work Environ Health. 2011;37(6):464–72. doi: 10.5271/sjweh.3182.

36. Salonen L, Alexanderson K, Rugulies R, et al. Combinations of Job Demands and Job Control and Future Trajectories of Sickness Absence and Disability Pension An 11-year Follow-up of Two Million Employees in Sweden. J Occup Environ Med. 2020;62(10):795–802. doi: 10.1097/JOM.0000000000001919.

37. Lidwall U, Bill S, Palmer E, et al. Mental disorder sick leave in Sweden: A population study. Work. 2018;59(2):259–72. doi: 10.3233/WOR-172672.

38. Foss L, Gravseth HM, Kristensen P, et al. Risk Factors for Long-Term Absence Due To Psychiatric Sickness: A Register-Based 5-Year Follow-Up From the Oslo Health Study. J Occup Environ Med. 2010;52(7):698–705. doi: 10.1097/JOM.0b013e3181e98731.

39. The Swedish Social Insurance Agency. Sjukfrånvaron på svensk arbetsmarknad: Sjukskrivningar längre än 14 dagar och avslut inom 180 dagar i olika branscher och yrken. [Sickness absence on the Swedish labour market: Sickness absence spells longer than 14 days and ended within 180 days in different branches of industry and occupations]. 2018. Report No.: 12.

For peer review only

For peer review only - http://bmjopen.bmj.com/site/about/guidelines.xhtml

Table 1. Characteristics of the study cohort in 2012.

	Total	
**Sex**	**n**	**%**
Women	598 965	*47.59*
Men	659 755	*52.41*
**Age group**		
16-24	63 788	*5.07*
25-34	271 754	*21.59*
35-44	371 803	*29.54*
45-54	322 900	*25.65*
55-64	117 802	*9.36*
65-67	110 673	*8.79*
**Type of living area**		
Large city	647 868	*51.47*
Medium-sized town	384 746	*30.57*
Rural or small town	226 106	*17.96*
**Educational level**		
Primary	61 256	*4.87*
Secondary	521 351	*41.42*
Tertiary	676 113	*53.71*
**Country of birth**		
Sweden	1 129 201	*89.71*
Other Nordic country	26 478	*2.10*
Other EU25 country	25 010	*1.99*
Other countries	78 031	*6.20*
**Family composition**		
Couple without children <18 at home	167 791	*13.33*
Couple with children <18 at home	595 073	*47.28*
Single without children <18 at home	411 846	*32.72*
Single with children <18 at home	84 010	*6.67*
**Branch of industry**		
Manufacturing	259 419	*20.61*
Service	543 452	*43.17*
Trade, hotel, restaurant	161 308	*12.82*
Transport	54 978	*4.37*
Construction	49 938	*3.97*
Education, care, nursing, social services	189 083	*15.02*
Unknown	542	*0.04*
**Income (SEK)**		
7920-87 999	23 701	*1.88*
88 000-175 999	81 257	*6.46*
176 000-329 999	355 583	*28.25*
330 000-439 999	347 772	*27.63*
>440 000	450 407	*35.78*
**Number of SA net days in 2012 in SA spells >14 gross days**		

For peer review only

0	1 170 169	*92.96*
1-14	27 895	*2.22*
15-30	17 001	*1.35*
31-90	24 292	*1.93*
91-180	10 885	*0.86*
181-365	7405	*0.59*
366*	1071	*0.09*
**Total**	1 258 720	*100.00*
**SA diagnoses in 2012****		
Mental diagnoses	27 765	*2.21*
Musculoskeletal diagnoses	18 502	*1.44*
Injury	9179	*0.72*
Cancer	5294	*0.41*
Circulatory diagnoses	3884	*0.30*
Pregnancy-related diagnoses	7005	*0.55*
Other diagnoses	23 539	*1.83*

* 2012 was a leap yea year. ** Individuals could have had several SA spells with different diagnoses.

SA = sickness absence. SEK = Swedish Krona

For peer review only

Table 2. Multinomial regression with five clusters of sickness absence (SA) and disability pension (DP) days/year among privately employed white-collar workers, odds ratios (OR) with their 95% confidence intervals, Cluster 1 'low or no SA or DP' was used as reference group.

	Cluster 2) SA due to other diagnoses		Cluster 3) SA due to mental diagnoses		Cluster 4) ineligible for SA and DP		Cluster 5) DP	
	Crude OR	Adjusted OR	Crude OR	Adjusted OR	Crude OR	Adjusted OR	Crude OR	Adjusted OR
**Sex**								
Women	ref.	ref.	ref.	ref.	ref.	ref.	ref.	ref.
Men	0.38 (0.37 - 0.38)	0.47 (0.46 - 0.47)	0.30 (0.29 - 0.31)	0.38 (0.37 - 0.39)	1.18 (1.15 - 1.22)	1.13 (1.10 - 1.17)	0.32 (0.31 - 0.33)	0.69 (0.66 - 0.72)
**Age group**								
16-24	1.36 (1.31 - 1.41)	1.09 (1.05 - 1.14)	0.76 (0.73 - 0.80)	0.37 (0.35 - 0.39)	1.34 (1.25 - 1.44)	1.54 (1.42 - 1.66)	0.28 (0.24 - 0.34)	0.00 (0.00 – 0.00)
25-34	1.33 (1.30 - 1.36)	1.30 (1.27 - 1.33)	0.96 (0.94 - 0.99)	0.79 (0.77 - 0.81)	1.34 (1.28 - 1.4)	1.54 (1.47 - 1.61)	0.30 (0.27 - 0.33)	0.10 (0.09 - 0.11)
35-44	ref.	ref.	ref.	ref.	ref.	ref.	ref.	ref.
45-54	1.37 (1.34 - 1.40)	1.42 (1.39 - 1.45)	0.88 (0.86 - 0.90)	0.79 (0.77 - 0.82)	1.12 (1.07 - 1.17)	1.28 (1.22 - 1.34)	2.61 (2.50 - 2.74)	3.34 (3.18 - 3.51)
55-64	1.74 (1.70 - 1.79)	1.73 (1.67 - 1.78)	0.71 (0.68 - 0.74)	0.57 (0.54 - 0.59)	1.94 (1.85 - 2.05)	1.99 (1.88 - 2.10)	4.93 (4.69 - 5.18)	4.29 (4.04 - 4.55)
65-67	0.50 (0.48 - 0.52)	0.45 (0.43 - 0.47)	0.12 (0.11 - 0.13)	0.10 (0.09 - 0.11)	2.50 (2.38 - 2.62)	2.32 (2.20 - 2.45)	2.68 (2.53 - 2.83)	1.26 (1.18 - 1.35)
**Type of living area**								
Large city	ref.	ref.	ref.	ref.	ref.	ref.	ref.	ref.
Medium-sized town	1.02 (1.00 - 1.04)	1.02 (1.00 - 1.04)	0.99 (0.97 - 1.02)	1.06 (1.03 - 1.08)	0.75 (0.72 - 0.77)	0.82 (0.79 - 0.85)	1.70 (1.64 - 1.76)	1.05 (1.01 - 1.10)
Rural or small town	1.11 (1.08 - 1.13)	1.07 (1.05 - 1.09)	1.04 (1.01 - 1.07)	0.97 (0.94 - 0.99)	0.71 (0.68 - 0.74)	0.67 (0.64 - 0.70)	2.44 (2.34 - 2.54)	1.03 (0.99 - 1.08)
**Educational level**								
Primary	1.37 (1.32 - 1.42)	1.66 (1.60 - 1.73)	1.20 (1.15 - 1.26)	1.79 (1.71 - 1.87)	1.57 (1.48 - 1.66)	1.39 (1.32 - 1.48)	4.12 (3.89 - 4.36)	1.68 (1.57 - 1.79)
Secondary	1.41 (1.38 - 1.43)	1.34 (1.32 - 1.36)	1.25 (1.23 - 1.28)	1.22 (1.19 - 1.24)	0.78 (0.76 - 0.81)	0.86 (0.84 - 0.89)	2.68 (2.59 - 2.78)	1.50 (1.44 - 1.56)
Tertiary	ref.	ref.	ref.	ref.	ref.	ref.	ref.	ref.
**Country of birth**								
Sweden	ref.	ref.	ref.	ref.	ref.	ref.	ref.	ref.
Other Nordic country	1.21 (1.15 - 1.27)	1.04 (0.99 - 1.10)	1.13 (1.06 - 1.20)	0.86 (0.80 - 0.92)	3.90 (3.67 - 4.14)	3.25 (3.05 - 3.46)	1.69 (1.55 - 1.85)	0.80 (0.72 - 0.89)
Other EU25 country	1.00 (0.94 - 1.06)	1.07 (1.01 - 1.13)	1.00 (0.93 - 1.07)	1.11 (1.05 - 1.19)	4.03 (3.79 - 4.27)	4.49 (4.25 - 4.76)	0.86 (0.76 - 0.97)	0.31 (0.26 - 0.37)
Other countries	1.41 (1.37 - 1.45)	1.13 (1.10 - 1.17)	1.16 (1.12 - 1.21)	0.87 (0.84 - 0.91)	2.30 (2.20 - 2.41)	2.58 (2.46 - 2.70)	0.87 (0.81 - 0.93)	0.48 (0.44 - 0.52)
**Family composition**								
Couple without children <18 at home	ref.	ref.	ref.	ref.	ref.	ref.	ref.	ref.
Couple with children <18 at home	0.94 (0.92 - 0.97)	0.98 (0.95 – 1.00)	1.65 (1.59 - 1.71)	0.79 (0.76 - 0.82)	0.52 (0.50 - 0.54)	0.62 (0.59 - 0.65)	0.36 (0.34 - 0.37)	0.71 (0.67 - 0.74)
Single without children <18 at home	1.13 (1.10 - 1.16)	1.19 (1.15 - 1.22)	1.71 (1.65 - 1.78)	0.93 (0.90 - 0.97)	0.97 (0.93 - 1.01)	1.04 (0.99 - 1.08)	0.53 (0.50 - 0.55)	1.22 (1.16 - 1.28)

For peer review only - http://bmjopen.bmj.com/site/about/guidelines.xhtml

Single with children <18 at home	1.69 (1.63 - 1.74)	1.35 (1.30 - 1.40)	3.68 (3.52 - 3.84)	1.31 (1.25 - 1.36)	0.61 (0.57 - 0.66)	0.54 (0.50 - 0.59)	0.87 (0.82 - 0.92)	1.05 (0.98 - 1.13)
**Branch of industry**								
Manufacturing	0.82 (0.80 - 0.84)	0.89 (0.87 - 0.91)	0.69 (0.67 - 0.71)	0.83 (0.80 - 0.85)	1.01 (0.97 - 1.05)	1.07 (1.03 - 1.11)	0.63 (0.60 - 0.66)	1.11 (1.05 - 1.17)
Service	ref.	ref.	ref.	ref.	ref.	ref.	ref.	ref.
Trade, hotel, restaurant	1.11 (1.08 - 1.13)	0.89 (0.87 - 0.91)	1.04 (1.01 - 1.07)	0.92 (0.89 - 0.95)	0.89 (0.85 - 0.94)	1.10 (1.05 - 1.16)	0.93 (0.89 - 0.98)	0.67 (0.63 - 0.71)
Transport	1.19 (1.14 - 1.23)	1.02 (0.98 - 1.07)	0.91 (0.87 - 0.96)	0.85 (0.81 - 0.90)	1.01 (0.94 - 1.09)	1.11 (1.03 - 1.19)	1.08 (1.00 - 1.16)	0.66 (0.60 - 0.72)
Construction	0.92 (0.88 - 0.96)	1.01 (0.97 - 1.06)	0.67 (0.63 - 0.71)	0.45 (0.42 - 0.49)	0.71 (0.65 - 0.77)	0.98 (0.90 - 1.06)	0.95 (0.88 - 1.04)	0.78 (0.71 - 0.86)
Education, care, nursing, social services	2.03 (1.99 - 2.07)	1.34 (1.31 - 1.37)	1.82 (1.77 - 1.86)	1.19 (1.16 - 1.22)	0.98 (0.94 - 1.03)	1.00 (0.96 - 1.05)	1.73 (1.66 - 1.80)	0.80 (0.76 - 0.83)
**Income (SEK)**								
7920-87 999	0.68 (0.64 - 0.72)	0.62 (0.58 - 0.66)	0.66 (0.62 - 0.71)	0.75 (0.70 - 0.81)	4.25 (3.99 - 4.52)	3.81 (3.55 - 4.10)	1.80 (1.67 - 1.94)	4.50 (4.17 - 4.87)
88 000-175 999	0.90 (0.87 - 0.93)	0.94 (0.91 - 0.98)	1.00 (0.96 - 1.03)	1.17 (1.13 - 1.21)	1.37 (1.29 - 1.46)	1.06 (0.99 - 1.14)	4.36 (4.21 - 4.52)	8.90 (8.57 - 9.25)
176 000-329 999	ref.	ref.	ref.	ref.	ref.	ref.	ref.	ref.
330 000-439 999	0.67 (0.66 - 0.69)	0.89 (0.87 - 0.91)	0.57 (0.55 - 0.58)	0.73 (0.71 - 0.74)	0.81 (0.77 - 0.84)	1.20 (1.15 - 1.25)	0.16 (0.15 - 0.17)	0.12 (0.11 - 0.12)
>440 000	0.39 (0.38 - 0.40)	0.58 (0.57 - 0.60)	0.30 (0.29 - 0.31)	0.45 (0.44 - 0.47)	1.03 (0.99 - 1.07)	1.56 (1.49 - 1.63)	0.05 (0.05 - 0.06)	0.00 (0.00 – 0.00)
**Number of SA net days in 2012**								
0	ref.	ref.	ref.	ref.	ref.	ref.	ref.	ref.
1-14	3.89 (3.76 - 4.03)	3.59 (3.47 - 3.72)	4.47 (4.29 - 4.66)	2.91 (2.77 - 3.05)	1.60 (1.45 - 1.75)	1.72 (1.56 - 1.89)	5.71 (5.35 - 6.09)	3.36 (3.10 - 3.65)
15-30	4.17 (3.99 - 4.35)	2.80 (2.67 - 2.94)	4.97 (4.73 - 5.23)	4.04 (3.84 - 4.26)	2.10 (1.89 - 2.33)	1.22 (1.06 - 1.41)	5.48 (5.04 - 5.96)	3.27 (2.95 - 3.62)
31-90	4.56 (4.40 - 4.73)	4.10 (3.95 - 4.25)	6.50 (6.25 - 6.76)	4.70 (4.50 - 4.90)	2.96 (2.74 - 3.20)	2.44 (2.23 - 2.67)	8.36 (7.87 - 8.89)	6.78 (6.34 - 7.26)
91-180	5.62 (5.32 - 5.93)	5.42 (5.14 - 5.72)	9.92 (9.40 - 10.46)	7.71 (7.28 - 8.17)	5.92 (5.4 - 6.49)	5.11 (4.60 - 5.67)	22.85 (21.45 - 24.35)	20.67 (19.25 - 22.2)
181-365	6.43 (6.00 - 6.89)	4.73 (4.41 - 5.08)	16.10 (15.16 - 17.10)	10.92 (10.25 - 11.63)	13.88 (12.74 - 15.11)	13.60 (12.42 - 14.89)	47.31 (44.34 - 50.49)	22.75 (21.08 - 24.55)
366*	7.86 (6.45 - 9.57)	2.65 (2.07 - 3.39)	27.08 (23.16 - 31.67)	4.76 (3.78 - 5.98)	36.19 (30.22 - 43.33)	50.60 (42.93 - 59.65)	100.07 (85.89 - 116.59)	61.44 (52.82 - 71.47)
**SA diagnoses in 2012**								
Mental diagnoses	3.31 (3.18 - 3.44)	2.80 (2.69 - 2.91)	11.74 (11.39 - 12.10)	8.27 (8.00 - 8.54)	1.87 (1.71 - 2.05)	2.44 (2.24 - 2.65)	8.76 (8.32 - 9.22)	6.71 (6.32 - 7.12)
Musculoskeletal diagnoses	4.02 (3.86 - 4.19)	3.16 (3.03 - 3.30)	3.06 (2.90 - 3.24)	2.47 (2.33 - 2.62)	1.88 (1.69 - 2.08)	1.54 (1.37 - 1.73)	10.54 (9.98 - 11.13)	6.63 (6.23 - 7.06)
Injury	3.31 (3.12 - 3.52)	3.09 (2.91 - 3.29)	2.35 (2.16 - 2.55)	2.43 (2.24 - 2.64)	1.94 (1.69 - 2.23)	2.66 (2.35 - 3.02)	4.95 (4.49 - 5.45)	3.23 (2.88 - 3.61)
Cancer	4.02 (3.71 - 4.37)	4.04 (3.73 - 4.37)	2.09 (1.83 - 2.38)	1.80 (1.57 - 2.06)	23.29 (21.69 - 25.01)	19.35 (17.86 - 20.97)	4.83 (4.19 - 5.58)	3.04 (2.61 - 3.55)
Circulatory diagnoses	3.14 (2.85 - 3.46)	3.32 (2.97 - 3.71)	1.99 (1.72 - 2.30)	2.30 (1.94 - 2.72)	3.60 (3.06 - 4.25)	6.61 (5.84 - 7.48)	11.96 (10.78 - 13.27)	18.02 (16.09 - 20.19)
Other diagnoses	5.09 (4.92 - 5.28)	3.93 (3.79 - 4.08)	4.29 (4.10 - 4.49)	3.56 (3.40 - 3.73)	2.79 (2.57 - 3.03)	1.25 (1.11 - 1.41)	9.67 (9.18 - 10.19)	7.25 (6.82 - 7.69)

* 2012 was a leap year, thus those people were on SA for full-time all that.

For peer review only

For peer review only - http://bmjopen.bmj.com/site/about/guidelines.xhtml

For peer review only

For peer review only - http://bmjopen.bmj.com/site/about/guidelines.xhtml

Supplementary Table 1. The distribution of sociodemographic/socioeconomic variables in each cluster of sickness absence (SA) and disability pension among privately employed white-collar workers

	Cluster 1) low or no SA or DP		Cluster 2) SA due to other diagnoses		Cluster 3) SA due to mental diagnoses		Cluster 4) ineligible for SA and DP		Cluster 5) DP	
	n	%	n	%	n	%	n	%	n	%
**Sex**										
Women	512 131	*45.0*	45 859	*68.4*	32 110	*73.2*	7416	*40.9*	11 277	*71.7*
Men	626 646	*55.0*	21 138	*31.6*	11 761	*26.8*	10 734	*59.1*	4444	*28.3*
**Age group**										
16-24	58 138	*5.1*	3864	*5.8*	2032	*4.6*	924	*5.1*	132	*0.8*
25-34	245 816	*21.6*	16 017	*23.9*	10 857	*24.7*	3891	*21.4*	588	*3.7*
35-44	338 445	*29.7*	16 566	*24.7*	15 502	*35.3*	4008	*22.1*	2700	*17.2*
45-54	287 310	*25.2*	19 226	*28.7*	11 588	*26.4*	3819	*21.0*	5993	*38.1*
55-64	102 487	*9.0*	8736	*13.0*	3326	*7.6*	2358	*13.0*	4030	*25.6*
65-67	106 581	*9.4*	2588	*3.9*	566	*1.3*	3150	*17.4*	2278	*14.5*
**Type of living area**										
Large city	589 869	*51.8*	33 849	*50.5*	22 600	*51.5*	10 778	*59.4*	5548	*35.3*
Medium-sized town	347 373	*30.5*	20 336	*30.4*	13 230	*30.2*	4745	*26.1*	5552	*35.3*
Rural or small town	201 535	*17.7*	12 812	*19.1*	8041	*18.3*	2627	*14.5*	4621	*29.4*
**Educational level**										
Primary	54 084	*4.7*	3689	*5.5*	2253	*5.1*	1442	*7.9*	1681	*10.7*
Secondary	461 868	*40.6*	32 312	*48.2*	20 029	*45.7*	6127	*33.8*	9339	*59.4*
Tertiary	622 825	*54.7*	30 996	*46.3*	21 589	*49.2*	10 581	*58.3*	4701	*29.9*
**Country of birth**										
Sweden	1 023 594	*89.9*	58 529	*87.4*	38 952	*88.8*	13 606	*75.0*	14 079	*89.6*
Other Nordic country	23 540	*2.1*	1622	*2.4*	1009	*2.3*	1219	*6.7*	548	*3.5*
Other EU25 country	22 782	*2.0*	1301	*1.9*	866	*2.0*	1219	*6.7*	269	*1.7*
Other countries	68 861	*6.0*	5545	*8.3*	3044	*6.9*	2106	*11.6*	825	*5.2*
**Family composition**										
Couple without children <18 at home	15 3085	*13.4*	8510	*12.7*	3455	*7.9*	3310	*18.2*	3991	*25.4*
Couple with children <18 at home	543 569	*47.7*	28 512	*42.6*	20 232	*46.1*	6087	*33.5*	5034	*32.0*
Single without children <18 at home	371 865	*32.7*	23 388	*34.9*	14 354	*32.7*	7822	*43.1*	5102	*32.5*

For peer review only

Single with children <18 at home	70 258	*6.2*	6587	*9.8*	5830	*13.3*	931	*5.1*	1594	*10.1*
**Sector**										
Manufacturing	242 538	*21.3*	10 409	*15.5*	6237	*14.2*	4000	*22.0*	2068	*13.2*
Service	495 208	*43.5*	25 925	*38.7*	18 436	*42.0*	8090	*44.6*	6738	*42.9*
Trade, hotel, restaurant	146 137	*12.8*	8461	*12.6*	5636	*12.8*	2135	*11.8*	1855	*11.8*
Transport	49 760	*4.4*	3091	*4.6*	1686	*3.8*	822	*4.5*	728	*4.6*
Construction	46 164	*4.1*	2228	*3.3*	1147	*2.6*	534	*2.9*	598	*3.8*
Education, care, nursing, social services	158 460	*13.9*	16 861	*25.2*	10 718	*24.4*	2547	*14.0*	3733	*23.7*
**Income (SEK)**										
7920-87 999	304 420	*26.7*	26 911	*40.2*	19 263	*43.9*	4687	*25.8*	6798	*43.2*
88 000-175 999	20 142	*1.8*	1206	*1.8*	842	*1.9*	1317	*7.3*	810	*5.2*
176 000-329 999	66 039	*5.8*	5248	*7.8*	4159	*9.5*	1393	*7.7*	6430	*40.9*
330 000-439 999	318 059	*27.9*	18 897	*28.2*	11 372	*25.9*	3951	*21.8*	1161	*7.4*
>440 000	430 117	*37.8*	14 735	*22.0*	8235	*18.8*	6802	*37.5*	522	*3.3*
**Number of SA net days in 2012**										
0	1 080 290	*94.9*	53 898	*80.4*	32 433	*73.9*	15 296	*84.3*	9801	*62.3*
1 - 14	20 262	*1.8*	3935	*5.9*	2719	*6.2*	458	*2.5*	1054	*6.7*
15 - 30	12 121	*1.1*	2523	*3.8*	1808	*4.1*	360	*2.0*	602	*3.8*
31 - 90	16 252	*1.4*	3703	*5.5*	3178	*7.2*	682	*3.8*	1233	*7.8*
91 - 180	6145	*0.5*	1723	*2.6*	1828	*4.2*	515	*2.8*	1274	*8.1*
181 - 365	3353	*0.3*	1077	*1.6*	1619	*3.7*	659	*3.6*	1438	*9.1*
366*	352	*0.0*	138	*0.2*	286	*0.7*	180	*1.0*	319	*2.0*
**SA diagnoses in 2012**										
Mental diagnoses	16 509	*1.4*	3111	*4.6*	6460	*14.7*	486	*2.7*	1793	*11.4*
Musculoskeletal diagnoses	12 285	*1.1*	2815	*4.2*	1418	*3.2*	364	*2.0*	1620	*10.3*
Injury	6655	*0.6*	1278	*1.9*	597	*1.4*	205	*1.1*	444	*2.8*
Cancer	3058	*0.3*	718	*1.1*	245	*0.6*	1071	*5.9*	202	*1.3*
Circulatory diagnoses	2627	*0.2*	483	*0.7*	201	*0.5*	150	*0.8*	423	*2.7*
Other diagnoses	14 648	*1.3*	4171	*6.2*	2323	*5.3*	637	*3.5*	1760	*11.2*
**Total**	1 138 777	100.0	66 997	100.0	43 871	100.0	18 150	100.0	15 721	100.0

* 2012 was a leap year, thus those people were on SA for full-time all that.

For peer review only

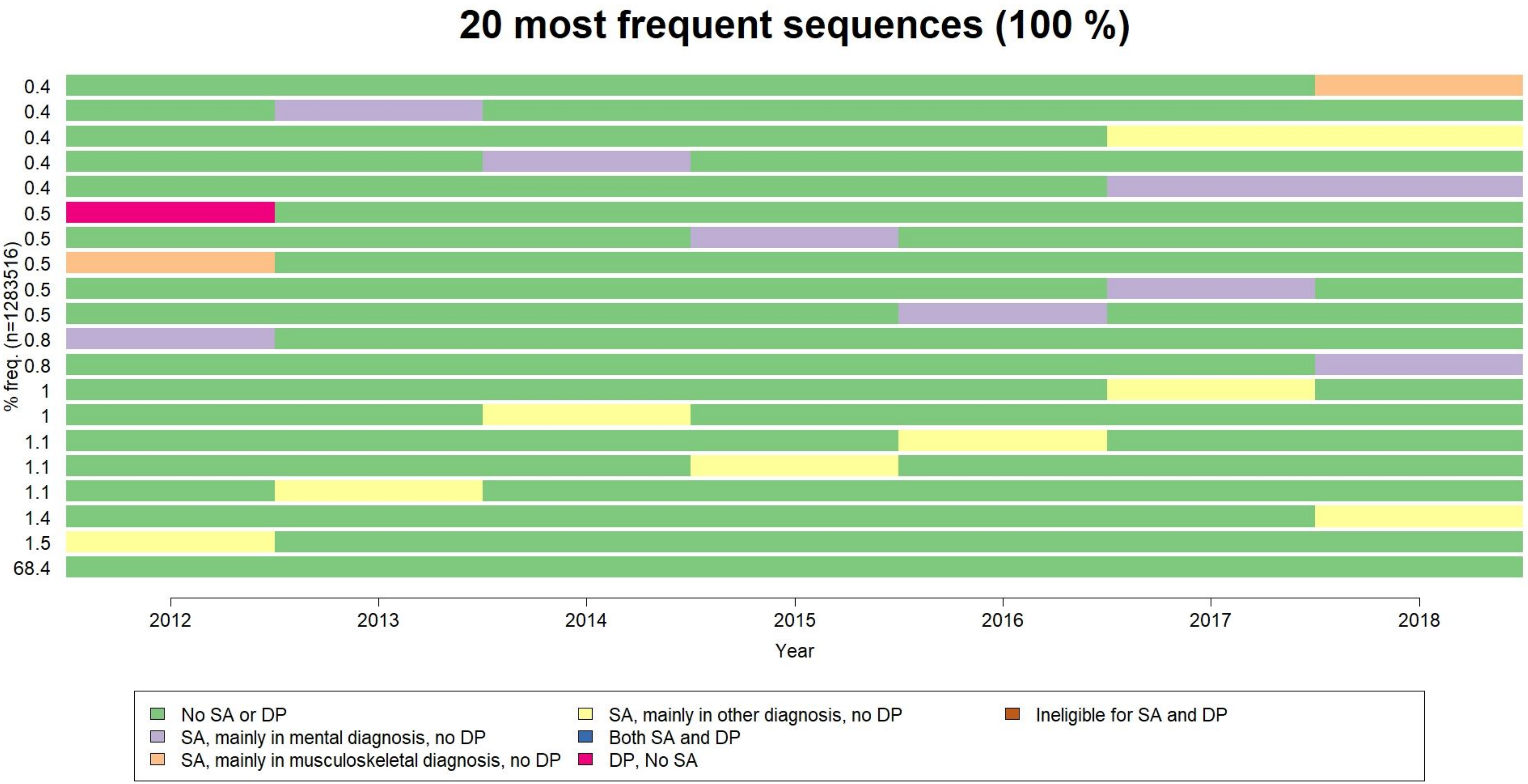

Supplementary Figure 1 Frequency plot for the 20 most frequent sequences of sickness absence (SA) and disability pension (DP) over 7 years among privately employed white-collar workers in Sweden

STROBE Statement—Checklist of items that should be included in reports of ***cohort studies***

	Item No	Recommendation	Page No
**Title and abstract**	1	(*a*) Indicate the study's design with a commonly used term in the title or the abstract	1
		(*b*) Provide in the abstract an informative and balanced summary of what was done and what was found	2
**Introduction**			
Background/rationale	2	Explain the scientific background and rationale for the investigation being reported	4
Objectives	3	State specific objectives, including any prespecified hypotheses	5
**Methods**			
Study design	4	Present key elements of study design early in the paper	5
Setting	5	Describe the setting, locations, and relevant dates, including periods of recruitment, exposure, follow-up, and data collection	5
Participants	6	(*a*) Give the eligibility criteria, and the sources and methods of selection of participants. Describe methods of follow-up	5
		(*b*) For matched studies, give matching criteria and number of exposed and unexposed	NA
Variables	7	Clearly define all outcomes, exposures, predictors, potential confounders, and effect modifiers. Give diagnostic criteria, if applicable	6
Data sources/ measurement	8*	For each variable of interest, give sources of data and details of methods of assessment (measurement). Describe comparability of assessment methods if there is more than one group	6
Bias	9	Describe any efforts to address potential sources of bias	5
Study size	10	Explain how the study size was arrived at	6
Quantitative variables	11	Explain how quantitative variables were handled in the analyses. If applicable, describe which groupings were chosen and why	6
Statistical methods	12	(*a*) Describe all statistical methods, including those used to control for confounding	6-7
		(*b*) Describe any methods used to examine subgroups and interactions	7
		(*c*) Explain how missing data were addressed	NA
		(*d*) If applicable, explain how loss to follow-up was addressed	NA
		(*e*) Describe any sensitivity analyses	NA
**Results**			
Participants	13*	(a) Report numbers of individuals at each stage of study—eg numbers potentially eligible, examined for eligibility, confirmed eligible, included in the study, completing follow-up, and analysed	5
		(b) Give reasons for non-participation at each stage	NA
		(c) Consider use of a flow diagram	NA
Descriptive data	14*	(a) Give characteristics of study participants (eg demographic, clinical, social) and information on exposures and potential confounders	Table 1
		(b) Indicate number of participants with missing data for each variable of interest	Table 1
		(c) Summarise follow-up time (eg, average and total amount)	5
Outcome data	15*	Report numbers of outcome events or summary measures over time	7-8

For peer review only - http://bmjopen.bmj.com/site/about/guidelines.xhtml

Main results	16	(*a*) Give unadjusted estimates and, if applicable, confounder-adjusted estimates and their precision (eg, 95% confidence interval). Make clear which confounders were adjusted for and why they were included	Table 2
		(*b*) Report category boundaries when continuous variables were categorized	Table 2
		(*c*) If relevant, consider translating estimates of relative risk into absolute risk for a meaningful time period	NA
Other analyses	17	Report other analyses done—eg analyses of subgroups and interactions, and sensitivity analyses	NA
**Discussion**			
Key results	18	Summarise key results with reference to study objectives	10
Limitations	19	Discuss limitations of the study, taking into account sources of potential bias or imprecision. Discuss both direction and magnitude of any potential bias	11
Interpretation	20	Give a cautious overall interpretation of results considering objectives, limitations, multiplicity of analyses, results from similar studies, and other relevant evidence	11
Generalisability	21	Discuss the generalisability (external validity) of the study results	11
**Other information**			
Funding	22	Give the source of funding and the role of the funders for the present study and, if applicable, for the original study on which the present article is based	12

*Give information separately for exposed and unexposed groups.

Note: An Explanation and Elaboration article discusses each checklist item and gives methodological background and published examples of transparent reporting. The STROBE checklist is best used in conjunction with this article (freely available on the Web sites of PLoS Medicine at http://www.plosmedicine.org/, Annals of Internal Medicine at http://www.annals.org/, and Epidemiology at http://www.epidem.com/). Information on the STROBE Initiative is available at http://www.strobe-statement.org.

For peer review only

BMJ Open

Sequence analysis of sickness absence and disability pension days in 2012–2018 among privately employed white-collar workers in Sweden: a prospective cohort study

Journal:	*BMJ Open*
Manuscript ID	bmjopen-2023-078066.R1
Article Type:	Original research
Date Submitted by the Author:	17-Nov-2023
Complete List of Authors:	Salonen, Laura; Finnish Institute of Occupational Health<br>Alexanderson, Kristina; Karolinska Institute Division of Insurance Medicine<br>Farrants, Kristin ; Karolinska Institute Division of Insurance Medicine,
**Primary Subject Heading**:	Occupational and environmental medicine
Secondary Subject Heading:	Epidemiology, Public health
Keywords:	EPIDEMIOLOGIC STUDIES, EPIDEMIOLOGY, OCCUPATIONAL & INDUSTRIAL MEDICINE, PUBLIC HEALTH, REGISTRIES

SCHOLARONE™
Manuscripts

I, the Submitting Author has the right to grant and does grant on behalf of all authors of the Work (as defined in the below author licence), an exclusive licence and/or a non-exclusive licence for contributions from authors who are: i) UK Crown employees; ii) where BMJ has agreed a CC-BY licence shall apply, and/or iii) in accordance with the terms applicable for US Federal Government officers or employees acting as part of their official duties; on a worldwide, perpetual, irrevocable, royalty-free basis to BMJ Publishing Group Ltd ("BMJ") its licensees and where the relevant Journal is co-owned by BMJ to the co-owners of the Journal, to publish the Work in this journal and any other BMJ products and to exploit all rights, as set out in our licence.

The Submitting Author accepts and understands that any supply made under these terms is made by BMJ to the Submitting Author unless you are acting as an employee on behalf of your employer or a postgraduate student of an affiliated institution which is paying any applicable article publishing charge ("APC") for Open Access articles. Where the Submitting Author wishes to make the Work available on an Open Access basis (and intends to pay the relevant APC), the terms of reuse of such Open Access shall be governed by a Creative Commons licence – details of these licences and which Creative Commons licence will apply to this Work are set out in our licence referred to above.

Other than as permitted in any relevant BMJ Author's Self Archiving Policies, I confirm this Work has not been accepted for publication elsewhere, is not being considered for publication elsewhere and does not duplicate material already published. I confirm all authors consent to publication of this Work and authorise the granting of this licence.

For peer review only

Sequence analysis of sickness absence and disability pension days in 2012–2018 among privately employed white-collar workers in Sweden: a prospective cohort study

Laura Salonen[1], Kristina Alexanderson[2], & Kristin Farrants[2]

1 Finnish Institute of Occupational Health, Finland

2 Division of Insurance Medicine, Department of Clinical Neuroscience, Karolinska Institutet, Sweden

Corresponding author: Kristin Farrants, kristin.farrants@ki.se

For peer review only

ABSTRACT

Objective: To explore sequences of sickness absence (SA) and disability pension (DP) days from 2012 through 2018 among privately employed white-collar workers.

Methods: A seven-year prospective cohort study of all 1,283,516 privately employed white-collar workers in Sweden in 2012 aged 16–67. Microdata from nationwide registers were used for sequence analysis to describe clusters of individuals who followed similar development of SA and DP net days/year, and multinomial logistic regression to analyze associations between sociodemographic variables and belonging to each observed cluster of sequences. Odds ratios (OR) and 95% confidence intervals (CI) were adjusted for baseline sociodemographics.

Results: We identified five clusters of SA and DP sequences: 1) 'low or no SA or DP' (88.7% of the population), 2) 'SA due to other than mental diagnosis' (5.2%), 3) 'SA due to mental diagnosis' (3.4%), 4) 'not eligible for SA or DP' (1.4%), and 5) 'DP' (1.2%). Men, highly educated, born outside Sweden, and high-income earners were more likely to belong to the first and the fourth cluster (ORs range 1.13-4.49). The second, third, and fifth clusters consisted mainly of women, low educated, and low-income (ORs range 1.22-8.90). There were only small differences between branches of industry in adjusted analyses, and many were not significant.

Conclusion: In general, only a few privately employed white-collar workers had SA and even fewer had DP during the seven-year follow-up. The risk of belonging to a cluster characterized by SA or DP varied by sex, levels of education and income, and other sociodemographic factors.

Keywords: sick leave, sequence analysis, private sector

Strengths and limitations of this study:

- Detailed sociodemographic microdata, linked from different population-based registers, about a cohort of all privately employed white-collar workers in Sweden in 2012
- Long study period (7 years) with no loss to follow-up and no bias from self-reports

For peer review only - http://bmjopen.bmj.com/site/about/guidelines.xhtml

- Use of sequence analysis to capture the heterogeneity of the different sickness absence and disability pension patterns over time
- How many and which states to include in the analyses is dependent on researcher judgement and thus can be arbitrary

For peer review only

INTRODUCTION

Sickness absence (SA) and disability pension (DP) have adverse consequences for individuals, their employers, and welfare states. The development of SA and DP is often a long process and varies with type of occupation and work tasks (1,2). In general, white-collar workers have a lower risk of SA and DP compared to other occupational groups (3–5). Nevertheless, they constitute a large part of the workforce—approximately half in Sweden in 2018 (6), and about half of them are privately employed (7). Thus, work incapacity in this group can impose high costs for employees, employers, and the welfare state. To prevent work incapacity in this population, more knowledge is needed on the determinants and the process of developing long-term work incapacity.

Previous research on SA and DP within specific occupations or occupational groups has mainly focused on so-called high-risk groups, e.g., manual workers and blue-collar workers (8–14), while studies on white-collar workers are scarce. Those conducted are mainly based on small sample sizes (15,16). Most of the research on SA and DP among white-collar employees has focused on publicly employed white-collar employees; for instance, the Whitehall-II studies of British civil servants (17–20). These studies have shown that there are differences in rates of SA among white-collar workers by age, gender, education, occupational status, and other sociodemographic and socioeconomic factors.

Studies on white-collar workers in the *private* sector are even more limited. In general, large-scale studies have demonstrated that SA rates in the private sector are generally lower than in the public sector (21,22). There are several studies on SA and/or DP among private-sector employees, however, hardly any among specifically among white-collar workers, despite how many it concerns. Moreover, the few such studies are mainly based on small, selected populations, have large drop-out rates, and are mainly based on self-reported data (5,23–25). So far, only three large-scale studies on private sector white-collar employees have been published: two Swedish studies (3,26) and a Greek study on private sector employees (also including blue-collar employees) that found a smaller SA rate in the shipyard industry than in other industries (27). The results of the two Swedish studies showed that the risk of SA and DP—and the risk of belonging to an adverse SA/DP trajectory—differed among white-collar workers by age, sex, education, a branch of industry, psychosocial exposures at work, and other sociodemographic factors. Further, none of these studies have accounted for transitions between other labour market states in addition to SA and DP, such as employment and unemployment. More studies using full population data with a longitudinal research design are needed to increase the knowledge base.

Moreover, both SA and DP are complex phenomena affected by many factors. Both increase with age, are lower in people with higher education and non-immigrants, and differ by sex; in most occupations, women have higher SA/DP levels than men, hence it is important to include such factors in studies of future SA/DP (3,28–30).

For peer review only - http://bmjopen.bmj.com/site/about/guidelines.xhtml

Sequence analysis is a good method to study developments over time. Unlike more traditionally used methods, such as event history analysis or growth curve models, sequence analysis can describe the duration and frequency of multiple categorical statuses. This holistic perspective is essential in providing an overview of the future development of SA and DP, and in identifying potential sub-groups within a population who share particular patterns in terms of such SA and DP.

The aim of this study was to identify sequences of white-collar workers in the private sector who follow future similar sequences of SA and DP days/year and second, to analyze the sociodemographic and diagnostic composition of the observed clusters of SA and DP.

METHODS

Data sources and population

We conducted a seven-year prospective population-based cohort study. We used microdata from the following three nationwide Swedish administrative registers, linked at the individual level by personal identity number (PIN; a unique 10-digit number assigned to all Swedish residents) (31):

-The Longitudinal Integration Database for Health Insurance and Labour Market Studies (LISA) held by Statistics Sweden, to identify the study cohort and for information on socio-demographic characteristics at baseline 2012 and regarding being in paid work or not in 2012-2018 (see Variables below) or emigrating in 2013-2018.

-The MicroData for Analysis of the Social Insurance database (MiDAS) held by the Swedish Social Insurance Agency, for information on SA and DP in the years 2012–2018 regarding SA and DP (dates, grades (full- or part-time), and diagnoses), and

-The Cause of Death Register held by the National Board of Health and Welfare for year of death.

The study population consisted of all individuals aged 18–67 years who lived in Sweden on both 31 December 2011 and 31 December 2012, who had an occupational code according to the Swedish Standard for Occupational Classification indicating a white-collar occupation (3), were employed at a private-sector company during 2012, and had an income from work, parental benefits, SA, and/or DP that amounted to at least 75% of the necessary income level to qualify for SA benefits from the Social Insurance Agency (7920 Swedish krona (SEK) in 2012, approx. €910 by the 2012 exchange rate, updated yearly in line with inflation). We excluded unemployed, self-employed, and those who were on full-time DP for the entire year 2012 (n=461). The total study cohort included 1,283,516 individuals.

Public sickness absence insurance in Sweden

In Sweden, all residents aged at least 16 years with an income from work or unemployment benefits who have a reduced work capacity due to morbidity are covered by the national public SA insurance

For peer review only - http://bmjopen.bmj.com/site/about/guidelines.xhtml

(32). A physician's certificate is required after seven days. After an unpaid qualifying day, the employer pays the following 13 SA days, after which SA benefits are paid by the Social Insurance Agency. For unemployed, the Social Insurance Agency pays after the first qualifying day. Thus, we excluded SA spells shorter than 15 days, in order not to introduce bias, since we only had information of SA spells exceeding 14 days for the employed. There was no limitation regarding how long a SA spell could be ongoing for. Residents in Sweden aged 19–64 years, whose work capacity is long-term or permanently reduced can be granted DP from the Social Insurance Agency. SA covers about 80% and DP about 65% of lost income, both up to a certain level. Both SA and DP can be granted for part- or full-time (25%, 50%, 75%, or 100% of ordinary work hours). This means that people can be on partial SA and DP at the same time.

Sociodemographic and work-related variables

We included information on sex, age group, country of birth, educational level, family composition, type of living area, and branch of industry based on the Swedish Standard for Industry Classification (SNI) categorized into the following six groups: manufacturing, services, transport, construction and installation, care and education, or commerce and hospitality. All variables were measured at the baseline year 2012.

Measures on sickness absence and disability pension

We used SA net days/year and DP net days/year as outcomes. Net days were calculated so that partial days of SA or DP were combined, e.g., two days of part-time SA for 50% were summed to one net day, and a similar procedure was used for DP days. The first 14 days of SA spells (>14 days) were counted as being of the same grade as day 15 for the purpose of calculating net days. The number of SA net days in 2012 were categorized as shown in Table 1. The SA diagnoses were categorized into the following seven International Classification of Diseases (ICD-10) groups (33): Cancer (C00-D48), Mental diagnoses (F00-F99 and Z73), Circulatory diseases (I00-I99), Musculoskeletal diagnoses (M00-M99), Pregnancy-related diagnoses (O00-O99), Injuries (S00-T98), and other diagnostic groups (all others, including missing diagnosis (approximately 1% of all spells). In the multinomial logistic regression, pregnancy-related diagnoses were dropped, as no men could have pregnancy-related diagnoses, which made it highly correlated with sex.

In analyses of the yearly states of SA/DP, all diagnoses other than mental and musculoskeletal diseases were combined to form one status. Any DP, regardless of diagnosis, was considered as one group.

Sequence analysis and multinomial regression analysis

For peer review only - http://bmjopen.bmj.com/site/about/guidelines.xhtml

We used sequence analysis to examine different statuses of SA and DP days/year, and the transitions between such statuses. SA and DP status was measured on a yearly basis for each of the seven follow-up years and was coded into one of the following seven statuses:

1) no SA or DP,
2) SA due to mental diagnoses but no DP,
3) SA due to musculoskeletal diagnoses but no DP,
4) SA due to other diagnoses but no DP,
5) both SA and DP,
6) only DP, and
7) ineligible for SA and DP (due to being emigrated, dead, retired, or having no qualifying income from work or work-related benefits).

Individuals who had SA in more than one diagnostic category were assigned to the diagnostic category they had the most days in that year. We illustrated the individual and proportional changes in SA/DP statuses over time with sequence index plots and status proportion plots (34).

We used optimal matching (OM) method to group similar sequences with each other. OM measures the dissimilarities through the changes needed to make two sequences identical (35). In other words, the OM algorithm creates metric distances between two sequences, which can be defined as the minimum combination of replacements, insertion and deletions to transform one sequence to another (36). We used R statistical program version 4.1.0 and packages TraMineR and nnet for the sequence analysis.

We used multinomial regression analysis to analyze how sociodemographic characteristics and branch of industry were associated with each of the obtained clusters, using the first cluster as the reference category. Odds ratios (ORs) with their 95% confidence intervals were reported.

Patient and public involvement

Representatives from the private white-collar sector in Sweden, both for employees and employers (the labour union PTK, the Confederation of Swedish Enterprise, and Alecta) were involved in selecting the research questions through joint meetings throughout the project period, and afterwards in disseminating results.

RESULTS

Characteristics of the study population

For peer review only - http://bmjopen.bmj.com/site/about/guidelines.xhtml

Table 1 shows the characteristics of the study cohort of the 1,283,516 privately employed white-collar workers. There were slightly more men (52.4%) in the cohort. The largest age group was those aged 35-44 years (29.5%), over half lived in a large city (51.5%) and had a tertiary education (53.7%). The majority were born in Sweden (89.7%), and almost half were married or cohabiting and having children below the age of 18 at home (47.3%). The largest group was the service industry (43.1%) and the largest income group was those who earned over 440,000 SEK (around 50,556 EUR according to the average 2012 conversion rate) per year (35.8%). A large majority did not have any SA in 2012: only around 7% had at least some SA spell >14 days. Around 2.2% had SA due to mental diagnoses, 1.4% due to musculoskeletal diagnoses and around 3.8% due to any other diagnoses.

[Table 1. Characteristics of the study population in 2012.]

Clusters of sickness absence and disability pension trajectories

We identified five different groups of sequences, i.e., clusters. Figure 1 shows each of the five clusters, as well as the proportion of individuals in each cluster and the proportion of individuals within the respective clusters in each state during each year. The sociodemographic characteristics of each cluster can be seen in Supplementary Table 1. The first cluster (n=1,138,777, 88.7% of all in the cohort) was the largest one, and almost 95% of individuals in this cluster had no SA or DP days. We called this cluster 'low or no SA or DP'.

Cluster 2 (n=66 997, 5.2%), which was the second largest, was characterized by SA due other than mental diagnosis, including those with mainly musculoskeletal diagnoses (Figure 1). We called this cluster 'SA due to other diagnoses'.

Cluster 3 (n=43 871, 3.4%) consisted mostly of those who had SA mainly due to mental diagnoses (Figure 1). We called this cluster 'SA due to mental diagnoses'.

Cluster 4 (n=18 150, 1.4%) was characterized by individuals who were not eligible for SA or DP since they either died, emigrated, or left the labor force (Figure 1). We called this cluster 'ineligible for SA and DP'.

The smallest cluster, Cluster 5 (n=15 721, 1.2%) was characterized by individuals who had either partial or full-time DP (Figure 1). We called this cluster 'DP'.

To better understand the most common SA and DP sequences, we examined the 20 most frequent sequences (Supplementary Figure 1). Most (68.4%) had no SA or DP during the follow-up. The remaining trajectories largely consisted of sequences where individuals had SA for one year and then returned to no SA or DP. Very few had DP during the follow-up.

For peer review only - http://bmjopen.bmj.com/site/about/guidelines.xhtml

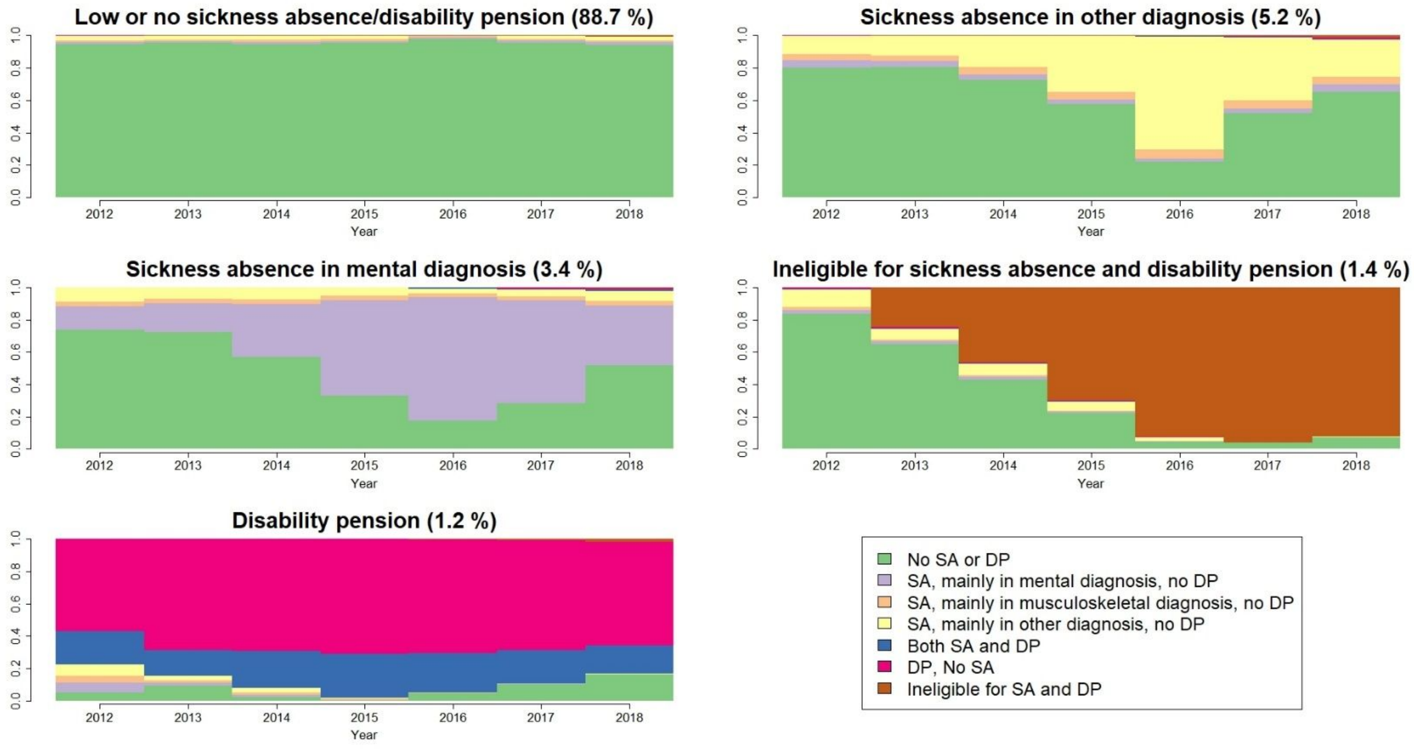

[Figure 1: density plot of sickness absence and disability pension visualizing the proportion of each sickness absence and disability pension status for each cluster over the follow-up]

The associations between individual characteristics and belonging to clusters of sickness absence and disability pension

To study how individual characteristics and SA at baseline were associated with cluster membership, we used multinomial regression analysis. Cluster 1 'low or no SA or DP' was used as the reference category since it was the largest and most homogenous in its sequence content (Table 2). Cluster 1 could be described as consisting of men of younger working-age, who had high levels of education and income, worked in service industry or in manufacturing and had no or only little SA in 2012 (Supplementary Table 1).

In the fully adjusted models, compared to cluster 1 'low or no SA or DP', women (men having an OR of 0.47 (95% CI 0.46-0.47)), over or under 35-44 year old (but not over 64 year old), those with less than tertiary education, who were born outside EU25 countries, living with children, worked in service industry or education, care, nursing, or social service industry, had a medium income, had any SA and especially SA due to cancer or due to 'other diagnoses' were more likely to belong to cluster 2 'SA due to mental diagnoses' (Table 2).

For peer review only - http://bmjopen.bmj.com/site/about/guidelines.xhtml

Women (men having an OR of 0.38 (95% CI 0.37-0.39)), 34-44 years old, who had less than tertiary education, who were single living with children, worked in education, care, nursing, or social service industry, had medium low income, had more than 188 SA days in 2012, especially due to mental diagnoses had the highest ORs of belonging to cluster 3 'SA due to mental diagnoses' (Table 2). The second and third clusters could be described as consisting of working-age women, who had less than tertiary education and medium income, who worked in education, care, nursing, or social service industry and had some SA in baseline year, especially due to mental diagnoses in the third cluster (Supplementary Table 1).

The OR for belonging to cluster 4 'ineligible for SA and DP' was the highest in men (OR 1.13; 95% CI 1.10-1.17), 65-67 years old, had primary education, lived without children, were born outside Sweden, had a very low income, who worked in trade, hotel, or restaurant industry or transport industry, had >180 SA days in 2012 and had SA due to circulatory diagnoses (Table 2). The fourth cluster could be described as consisting of men over 64 years old, who had primary education and were born outside Sweden, had low income, and had long-term SA in 2012, especially due to cancer (Supplementary Table 1).

The OR of belonging to cluster 5 'DP' were higher in women (OR 0.69 (95% CI 0.66-0.72) in men), 45-64 years old, who had less than tertiary education, were born in Sweden, who were single, worked in manufacturing, had low to medium low income, had at least 30 SA days in 2012 and especially those with SA due to circulatory diagnoses (Table 2). This fifth cluster could be described as consisting of older working age women, with low education, working in service industry with low income and long-term SA at baseline (Supplementary Table 1).

[Table 2: associations between sociodemographic factors and work disability clusters]

DISCUSSION

In this large prospective cohort study of all 1.3 million privately employed white-collar workers in Sweden in 2012, we analyzed the development of their future number of SA and DP days/year up through 2018. In general, most of the employees had no SA during the follow-up and DP was even rarer. We found five clusters of future SA and DP trajectories: 1) 'low or no SA or DP' (88.7% of all), 2) 'SA due to other (than mental) diagnosis' (5.2%), 3) 'SA due to mental diagnosis' (3.4%), 4) 'not eligible for SA or DP' (1.4%), and 5) 'DP' (1.2%). These results suggest that the majority of privately employed white-collar workers were doing well in terms of SA/DP.

We found some differences related to sociodemographic factors in terms of belonging to different sequence clusters. Many of those in cluster 1 'low or no SA or DP' were Swedish-born, 25–54-years

For peer review only - http://bmjopen.bmj.com/site/about/guidelines.xhtml

old, highly educated, and high-income earning men, who lived in a large city, and were married or cohabiting with children at home. The same sociodemographic characteristics are typically associated with lower risk of SA or DP in longitudinal nationwide studies (28,29).

We also found that female sex, low education, low income, and working in education, care, nursing, or social services were associated with a higher risk of belonging to clusters characterized by at least some SA or DP. Similar results were found in a previous cross-sectional study using the same data with number and prevalence of SA days as outcomes (3), as well as studies on SA and DP among white-collar workers in the retail and wholesale industry (26,37). In general, previous longitudinal population-based studies have consistently found that women, low educated, and low-and income earners (28,29), and those working in healthcare and service industries (22) have a higher risk of SA and/or DP. While these characteristics—low education, low income, and working in the healthcare industry—are usually considered as explanations to why blue-collar workers have a higher risk of SA or DP than white-collar workers (4,38), our results indicate that the same risk factors apply within white-collar employees working in the private sector. More knowledge is warranted regarding potential mechanisms behind this.

It is understandable that SA due to mental diagnoses constituted an independent cluster since among white-collar workers that is the most common specific diagnostic group of SA and/or DP (1,37,39–41). This cluster was more common among women, 34-44 years old, less than tertiary educated, low-income earners who worked in education, care, nursing and social industry, and had a long SA spell in 2012, which are known risk factors for SA due to mental diagnoses in general (42,43).

The cluster 'ineligible for SA or DP' had relatively many individuals aged ≥55 years, which makes sense since those who left paid work (e.g., through old-age pension) or died during the follow-up belonged to this cluster. There were also many highly educated and high-income earners, who typically are occupationally and geographically mobile, in this cluster. Relatively many of them were born outside Sweden; hence many of them probably emigrated from Sweden. Those who had SA due to cancer in 2012, had higher OR of belonging to this cluster than to any other cluster.

We found that the estimates for associations between branch of industry and cluster attenuated in the adjusted analyses, indicating that differences between the various branches of industry were more related to other factors. The Swedish Social Insurance Agency has found that in Sweden, occupation is more closely associated with SA than branch of industry (44). However, to what extent this is true within the group white-collar workers is unknown and should be further studied.

Strength and limitations

For peer review only - http://bmjopen.bmj.com/site/about/guidelines.xhtml

For peer review only

Strengths of this study are the use of a large, population-based cohort, that use of linked microdata from three high-quality nationwide registers without dropouts, the long prospective follow-up, and that all data were administrative, not self-reports with possible bias. Using sequence analysis allowed us to explore specific sub-groups in the development of SA and DP. Other strengths are that all included were covered by the same public SA and DP insurances, and the high employment-frequency in Sweden, that is, the healthy-worker effect did not bias the result much.

Since the study population consisted of privately employed white-collar workers in Sweden, the results cannot directly be generalized to other types of occupational populations or to other countries with other SA/DP systems or employment frequencies. Future studies might choose to explore other, or more specific SA states, regarding number of SA days or part- and full time SA/DP. As this was an observational study, no causal inferences can be drawn from the results.

CONCLUSION

In general, privately employed white-collar workers rarely had SA and even more rarely DP days during the seven-year follow-up. The risk of belonging to a cluster characterized by receiving SA varied by sex, levels of education and income, branch of industry, and other sociodemographic factors.

Funding: The study was funded by Alecta Insurance, Award/Grant number is not applicable. We utilized data from the REWHARD consortium supported by the Swedish Research Council (grant no. 2017-00624).

For peer review only - http://bmjopen.bmj.com/site/about/guidelines.xhtml

Data availability statement: The used data cannot be made publicly available due to privacy regulations. According to the General Data Protection Regulation, the Swedish law SFS 2018:218, the Swedish Data Protection Act, the Swedish Ethical Review Act, and the Public Access to Information and Secrecy Act, these types of sensitive data can only be made available for specific purposes that meets the criteria for access to this type of sensitive and confidential data as determined by a legal review. Professor Kristina Alexanderson (Kristina.alexanderson@ki.se) can be contacted regarding the data.

Ethics statements: The project was approved by the Regional Ethical Review Board in Stockholm, reference numbers 2009/1917-32, 2016/1533-32. In this observational study, based on population-based de-identified register data, informed consent was not applicable. All methods were performed in accordance with the relevant guidelines and regulations.

Patient consent for publication: Not applicable.

Acknowledgements: Not applicable.

Competing Interest: None declared.

Author Contributions: KF and KA planned and designed the study. KF supervised the analyses. LS wrote the first draft of the paper. All authors critically revised the paper for intellectual content. All authors approved the submission of the study.

REFERENCES

1. Helgadóttir B, Narusyte J, Ropponen A, Bergström G, Mather L, Blom V, et al. The role of occupational class on the association between sickness absence and disability pension: A Swedish register-based twin study. Scandinavian Journal of Work, Environment & Health. 2019. 20;45(6):622–30.

2. Salonen L, Blomgren J, Laaksonen M, Niemelä M. Sickness absence as a predictor of disability retirement in different occupational classes: a register-based study of a working-age cohort in Finland in 2007–2014. BMJ Open. 2018. 8(5):e020491.

3. Farrants K, Alexanderson K. Sickness absence among privately employed white-collar workers: A total population study in Sweden. Scand J Public Health. 2021. 49(2):159–67.

4. Piha K, Laaksonen M, Martikainen P, Rahkonen O, Lahelma E. Interrelationships between education, occupational class, income and sickness absence. The European Journal of Public Health. 2010. 20(3):276–80.

For peer review only - http://bmjopen.bmj.com/site/about/guidelines.xhtml

5. Väänänen A, Kalimo R, Toppinen-Tanner S, Mutanen P, Peiró JM, Kivimäki M, et al. Role clarity, fairness, and organizational climate as predictors of sickness absence: A prospective study in the private sector. Scandinavian Journal of Public Health. 2004. 32(6):426–34.

6. Statistics Sweden. Yrkesregistret med yrkesstatistik 2018: Yrkesstrukturen I Sverige [The Swedish Occupational Register with statistics 2018: The occupational structure in Sweden]. Available from: https://www.scb.se/hitta-statistik/statistik-efter-amne/arbetsmarknad/sysselsattning-forvarvsarbete-och-arbetstider/yrkesregistret-med-yrkesstatistik/. Accessed on 31 October 2023.

7. Alecta. Särskild statistik med anledning av coronapandemin. 2023. Available from: https://www.alecta.se/om-alecta/var-syn-pa-saken/var-statistik/coronasiffror/. Accessed on 31 October 2023.

8. Ahola K, Gould R, Virtanen M, Honkonen T, Aromaa A, Lonnqvist J. Occupational burnout as a predictor of disability pension: a population-based cohort study. Occupational and Environmental Medicine. 2009. 66(5):284–90.

9. Alexopoulos EC. Prognostic factors for respiratory sickness absence and return to work among blue collar workers and office personnel. Occupational and Environmental Medicine. 2001. 58(4):246–52.

10. Andersen LL, Fallentin N, Thorsen SV, Holtermann A. Physical workload and risk of long-term sickness absence in the general working population and among blue-collar workers: prospective cohort study with register follow-up. Occupational and Environmental Medicine. 2016. 73(4):246–53.

11. Arndt V. Construction work and risk of occupational disability: a ten year follow up of 14 474 male workers. Occupational and Environmental Medicine. 2005. 62(8):559–66.

12. Haukka E, Kaila-Kangas L, Luukkonen R, Takala EP, Viikari-Juntura E, Leino-Arjas P. Predictors of sickness absence related to musculoskeletal pain: a two-year follow-up study of workers in municipal kitchens. Scandinavian Journal of Work, Environment & Health. 2014. 40(3):278–86.

13. Järvholm B, Stattin M, Robroek SJ, Janlert U, Karlsson B, Burdorf A A. Heavy work and disability pension—a long term follow-up of Swedish construction workers. Scandinavian Journal of Work, Environment & Health. 2014. 40(4):335–42.

14. Rantonen O, Alexanderson K, Pentti J, Kjeldgård L, Hämäläinen J, Mittendorfer-Rutz E, et al. Trends in work disability with mental diagnoses among social workers in Finland and Sweden in 2005–2012. Epidemiology and Psychiatric Sciences. 2016. 1–11.

15. Krantz G, Lundberg U. Workload, work stress, and sickness absence in Swedish male and female white-collar employees. Scandinavian Journal of Social Medicine. 2006. 34(3):238–46.

For peer review only - http://bmjopen.bmj.com/site/about/guidelines.xhtml

16. Roelen CAM, Heymans MW, van Rhenen W, Groothoff JW, Twisk JWR, Bültmann U. Fatigue as Prognostic Risk Marker of Mental Sickness Absence in White Collar Employees. Journal of Occupational Rehabilitation. 2013.

17. Feeney A, North F, Head J, Canner R, Marmot M. Socioeconomic and sex differentials in reason for sickness absence from the Whitehall II Study. Occupational and Environmental Medicine. 1998. 55(2):91–8.

18. Head J, Ferrie JE, Alexanderson K, Westerlund H, Vahtera J, Kivimaki M. Diagnosis-specific sickness absence as a predictor of mortality: the Whitehall II prospective cohort study. BMJ. 2008. 337(oct02 2):a1469–a1469.

19. North F, Syme SL, Feeney A, Shipley MJ, Marmot MG. Psychosocial work environment and sickness absence among British civil servants: the Whitehall II study. American Journal of Public Health. 1996. 86:332–40.

20. Stansfeld S, Feeney A, Head J, Canner R, North F, Marmot M. Sickness absence for psychiatric illness: The Whitehall II study. Social Science & Medicine. 1995. 40(2):189–97.

21. Björkenstam E, Helgesson M, Gustafsson K, Virtanen M, Hanson LLM, Mittendorfer-Rutz E. Sickness absence due to common mental disorders in young employees in Sweden: are there differences in occupational class and employment sector? Social Psychiatry and Psychiatric Epidemiology. 2021. 57(5):1097–106.

22. Lund T, Labriola M, Villadsen E. Who is at risk for long-term sickness absence? A prospective cohort study of Danish employees. Work. 2007. 28(3):225–30.

23. Koopmans PC, Roelen CAM, Groothoff JW. Frequent and long-term absence as a risk factor for work disability and job termination among employees in the private sector. Occupational and Environmental Medicine. 2008. 65(7):494–9.

24. Lund T, Kivimäki M, Labriola M, Villadsen E, Christensen KB. Using administrative sickness absence data as a marker of future disability pension: the prospective DREAM study of Danish private sector employees. Occupational and Environmental Medicine. 2008. 65(1):28–31.

25. Väänänen A, Toppinen-Tanner S, Kalimo R, Mutanen P, Vahtera J, Peiró JM. Job characteristics, physical and psychological symptoms, and social support as antecedents of sickness absence among men and women in the private industrial sector. Social Science & Medicine. 2003. 57(5):807–24.

For peer review only

26. Farrants K, Alexanderson K. Trajectories of sickness absence and disability pension days among 189,321 white-collar workers in the trade and retail industry; a 7-year longitudinal Swedish cohort study. BMC Public Health. 2022. 22(1):1592.

27. Alexopoulos EC, Merekoulias G, Tanagra D, Konstantinou EC, Mikelatou E, Jelastopulu E. Sickness Absence in the Private Sector of Greece: Comparing Shipyard Industry and National Insurance Data. International Journal of Environmental Research and Public Health. 2012. 9(4):1171–81.

28. Allebeck P, Mastekaasa A, Swedish Council on Technology Assessment in Health Care (SBU). Chapter 5. Risk factors for sick leave - general studies. Scandinavian Journal of Public Health. 2004. 32(63_suppl):49–108.

29. Beemsterboer W, Stewart R, Groothoff J, Nijhuis F. A literature review on sick leave determinants (1984-2004). International Journal of Occupational Medicine and Environmental Health 2009. 22(2).

30. De Vries H, Fishta A, Weikert B, Rodriguez Sanchez A, Wegewitz U. Determinants of Sickness Absence and Return to Work Among Employees with Common Mental Disorders: A Scoping Review. J Occup Rehabil. 2018. 28(3):393–417.

31. Ludvigsson JF, Otterblad-Olausson P, Pettersson BU, Ekbom A. The Swedish personal identity number: possibilities and pitfalls in healthcare and medical research. European Journal of Epidemiology. 2009. 24(11):659–67.

32. Swedish Social Insurance Agency. Social Insurance in Figures 2021. Stockholm, Sweden; 2021. Available from: https://statistik.forsakringskassan.se/wps/wcm/connect/11bc72d6-4bbb-4893-8a3b-c9e9eae568f8/social-insurance-in-figures-2021.pdf?MOD=AJPERES&CVID=. Accessed on 31 October 2023.

33. WHO. International Classification of Diseases: Tenth Revision, ICD-10. 2010. Available from: http://apps.who.int/classifications/icd10/browse/2010/en. Accessed on 31 October 2023.

34. Aisenbrey S, Fasang AE. New Life for Old Ideas: The “Second Wave” of Sequence Analysis Bringing the “Course” Back Into the Life Course. Sociological Methods & Research. 2010. 38(3):420–62.

35. Abbott A, Forrest J. Optimal Matching Methods for Historical Sequences. Journal of Interdisciplinary History. 1986. 16(3):471–94.

36. Abbott A, Tsay A. Sequence Analysis and Optimal Matching Methods in Sociology: Review and Prospect. Sociological Methods & Research. 2000. 29(1):3–33.

For peer review only - http://bmjopen.bmj.com/site/about/guidelines.xhtml

37. Farrants K, Alexanderson K. Sickness Absence and Disability Pension in the Trade and Retail Industry: A Prospective Cohort Study of 192,000 White-Collar Workers in Sweden. Journal of Occupational and Environmental Medicine. 2022. 64(11):912–9.

38. Sumanen H, Pietiläinen O, Lahti J, Lahelma E, Rahkonen O. Interrelationships between education, occupational class and income as determinants of sickness absence among young employees in 2002–2007 and 2008–2013. BMC Public Health. 2015. 15(1):332.

39. Karolaakso T, Autio R, Näppilä T, Nurmela K, Pirkola S. Socioeconomic factors in disability retirement due to mental disorders in Finland. European Journal of Public Health. 2020. 30(6):1218–24.

40. Leinonen T, Pietiläinen O, Laaksonen M, Rahkonen O, Lahelma E, Martikainen P. Occupational social class and disability retirement among municipal employees—the contribution of health behaviors and working conditions. Scandinavian Journal of Work, Environment & Health. 2011. 37(6):464–72.

41. Salonen L, Alexanderson K, Rugulies R, Framke E, Niemelä M, Farrants K. Combinations of Job Demands and Job Control and Future Trajectories of Sickness Absence and Disability Pension An 11-year Follow-up of Two Million Employees in Sweden. Journal of Occupational & Environmental Medicine. 2020. 62(10):795–802.

42. Lidwall U, Bill S, Palmer E, Olsson Bohlin C. Mental disorder sick leave in Sweden: A population study. Work. 2018. 59(2):259–72.

43. Foss L, Gravseth HM, Kristensen P, Claussen B, Mehlum IS, Skyberg K. Risk Factors for Long-Term Absence Due To Psychiatric Sickness: A Register-Based 5-Year Follow-Up From the Oslo Health Study. Journal of Occupational & Environmental Medicine. 2010. 52(7):698–705.

44. The Swedish Social Insurance Agency. Sjukfrånvaron på svensk arbetsmarknad: Sjukskrivningar längre än 14 dagar och avslut inom 180 dagar i olika branscher och yrken. [Sickness absence on the Swedish labour market: Sickness absence spells longer than 14 days and ended within 180 days in different branches of industry and occupations]. 2018. Report No.: 12.

For peer review only

Table 1. Characteristics of the study cohort in 2012.

	Total	
**Sex**	**n**	**%**
Women	598 965	*47.59*
Men	659 755	*52.41*
**Age group**		
16-24	63 788	*5.07*
25-34	271 754	*21.59*
35-44	371 803	*29.54*
45-54	322 900	*25.65*
55-64	117 802	*9.36*
65-67	110 673	*8.79*
**Type of living area**		
Large city	647 868	*51.47*
Medium-sized town	384 746	*30.57*
Rural or small town	226 106	*17.96*
**Educational level**		
Primary	61 256	*4.87*
Secondary	521 351	*41.42*
Tertiary	676 113	*53.71*
**Country of birth**		
Sweden	1 129 201	*89.71*
Other Nordic country	26 478	*2.10*
Other EU25 country	25 010	*1.99*
Other countries	78 031	*6.20*
**Family composition**		
Couple without children <18 at home	167 791	*13.33*
Couple with children <18 at home	595 073	*47.28*
Single without children <18 at home	411 846	*32.72*
Single with children <18 at home	84 010	*6.67*
**Branch of industry**		
Manufacturing	259 419	*20.61*
Service	543 452	*43.17*
Trade, hotel, restaurant	161 308	*12.82*
Transport	54 978	*4.37*
Construction	49 938	*3.97*
Education, care, nursing, social services	189 083	*15.02*
Unknown	542	*0.04*
**Income (SEK)**		
7920-87 999	23 701	*1.88*
88 000-175 999	81 257	*6.46*
176 000-329 999	355 583	*28.25*
330 000-439 999	347 772	*27.63*
>440 000	450 407	*35.78*
**Number of SA net days in 2012 in SA spells >14 gross days**		

For peer review only

0	1 170 169	*92.96*
1-14	27 895	*2.22*
15-30	17 001	*1.35*
31-90	24 292	*1.93*
91-180	10 885	*0.86*
181-365	7405	*0.59*
366*	1071	*0.09*
**Total**	1 258 720	*100.00*
**SA diagnoses in 2012****		
Mental diagnoses	27 765	*2.21*
Musculoskeletal diagnoses	18 502	*1.44*
Injury	9179	*0.72*
Cancer	5294	*0.41*
Circulatory diagnoses	3884	*0.30*
Pregnancy-related diagnoses	7005	*0.55*
Other diagnoses	23 539	*1.83*

* 2012 was a leap year. ** Individuals could have had several SA spells with different diagnoses.

SA = sickness absence. SEK = Swedish Krona

For peer review only

Table 2. Multinomial regression with five clusters of sickness absence (SA) and disability pension (DP) days/year among privately employed white-collar workers, odds ratios (OR) with their 95% confidence intervals, Cluster 1 ‘low or no SA or DP’ was used as reference group.

	Cluster 2) SA due to other diagnoses		Cluster 3) SA due to mental diagnoses		Cluster 4) ineligible for SA and DP		Cluster 5) DP	
	Crude OR	Adjusted OR	Crude OR	Adjusted OR	Crude OR	Adjusted OR	Crude OR	Adjusted OR
**Sex**								
Women	ref.	ref.	ref.	ref.	ref.	ref.	ref.	ref.
Men	0.38 (0.37 - 0.38)	0.47 (0.46 - 0.47)	0.30 (0.29 - 0.31)	0.38 (0.37 - 0.39)	1.18 (1.15 - 1.22)	1.13 (1.10 - 1.17)	0.32 (0.31 - 0.33)	0.69 (0.66 - 0.72)
**Age group**								
16-24	1.36 (1.31 - 1.41)	1.09 (1.05 - 1.14)	0.76 (0.73 - 0.80)	0.37 (0.35 - 0.39)	1.34 (1.25 - 1.44)	1.54 (1.42 - 1.66)	0.28 (0.24 - 0.34)	0.00 (0.00—0.00)
25-34	1.33 (1.30 - 1.36)	1.30 (1.27 - 1.33)	0.96 (0.94 - 0.99)	0.79 (0.77 - 0.81)	1.34 (1.28 - 1.4)	1.54 (1.47 - 1.61)	0.30 (0.27 - 0.33)	0.10 (0.09 - 0.11)
35-44	ref.	ref.	ref.	ref.	ref.	ref.	ref.	ref.
45-54	1.37 (1.34 - 1.40)	1.42 (1.39 - 1.45)	0.88 (0.86 - 0.90)	0.79 (0.77 - 0.82)	1.12 (1.07 - 1.17)	1.28 (1.22 - 1.34)	2.61 (2.50 - 2.74)	3.34 (3.18 - 3.51)
55-64	1.74 (1.70 - 1.79)	1.73 (1.67 - 1.78)	0.71 (0.68 - 0.74)	0.57 (0.54 - 0.59)	1.94 (1.85 - 2.05)	1.99 (1.88 - 2.10)	4.93 (4.69 - 5.18)	4.29 (4.04 - 4.55)
65-67	0.50 (0.48 - 0.52)	0.45 (0.43 - 0.47)	0.12 (0.11 - 0.13)	0.10 (0.09 - 0.11)	2.50 (2.38 - 2.62)	2.32 (2.20 - 2.45)	2.68 (2.53 - 2.83)	1.26 (1.18 - 1.35)
**Type of living area**								
Large city	ref.	ref.	ref.	ref.	ref.	ref.	ref.	ref.
Medium-sized town	1.02 (1.00 - 1.04)	1.02 (1.00 - 1.04)	0.99 (0.97 - 1.02)	1.06 (1.03 - 1.08)	0.75 (0.72 - 0.77)	0.82 (0.79 - 0.85)	1.70 (1.64 - 1.76)	1.05 (1.01 - 1.10)
Rural or small town	1.11 (1.08 - 1.13)	1.07 (1.05 - 1.09)	1.04 (1.01 - 1.07)	0.97 (0.94 - 0.99)	0.71 (0.68 - 0.74)	0.67 (0.64 - 0.70)	2.44 (2.34 - 2.54)	1.03 (0.99 - 1.08)
**Educational level**								
Primary	1.37 (1.32 - 1.42)	1.66 (1.60 - 1.73)	1.20 (1.15 - 1.26)	1.79 (1.71 - 1.87)	1.57 (1.48 - 1.66)	1.39 (1.32 - 1.48)	4.12 (3.89 - 4.36)	1.68 (1.57 - 1.79)
Secondary	1.41 (1.38 - 1.43)	1.34 (1.32 - 1.36)	1.25 (1.23 - 1.28)	1.22 (1.19 - 1.24)	0.78 (0.76 - 0.81)	0.86 (0.84 - 0.89)	2.68 (2.59 - 2.78)	1.50 (1.44 - 1.56)
Tertiary	ref.	ref.	ref.	ref.	ref.	ref.	ref.	ref.
**Country of birth**								
Sweden	ref.	ref.	ref.	ref.	ref.	ref.	ref.	ref.
Other Nordic country	1.21 (1.15 - 1.27)	1.04 (0.99 - 1.10)	1.13 (1.06 - 1.20)	0.86 (0.80 - 0.92)	3.90 (3.67 - 4.14)	3.25 (3.05 - 3.46)	1.69 (1.55 - 1.85)	0.80 (0.72 - 0.89)
Other EU25 country	1.00 (0.94 - 1.06)	1.07 (1.01 - 1.13)	1.00 (0.93 - 1.07)	1.11 (1.05 - 1.19)	4.03 (3.79 - 4.27)	4.49 (4.25 - 4.76)	0.86 (0.76 - 0.97)	0.31 (0.26 - 0.37)
Other countries	1.41 (1.37 - 1.45)	1.13 (1.10 - 1.17)	1.16 (1.12 - 1.21)	0.87 (0.84 - 0.91)	2.30 (2.20 - 2.41)	2.58 (2.46 - 2.70)	0.87 (0.81 - 0.93)	0.48 (0.44 - 0.52)
**Family composition**								
Couple without children <18 at home	ref.	ref.	ref.	ref.	ref.	ref.	ref.	ref.
Couple with children <18 at home	0.94 (0.92 - 0.97)	0.98 (0.95—1.00)	1.65 (1.59 - 1.71)	0.79 (0.76 - 0.82)	0.52 (0.50 - 0.54)	0.62 (0.59 - 0.65)	0.36 (0.34 - 0.37)	0.71 (0.67 - 0.74)
Single without children <18 at home	1.13 (1.10 - 1.16)	1.19 (1.15 - 1.22)	1.71 (1.65 - 1.78)	0.93 (0.90 - 0.97)	0.97 (0.93 - 1.01)	1.04 (0.99 - 1.08)	0.53 (0.50 - 0.55)	1.22 (1.16 - 1.28)

For peer review only - http://bmjopen.bmj.com/site/about/guidelines.xhtml

Single with children <18 at home	1.69 (1.63 - 1.74)	1.35 (1.30 - 1.40)	3.68 (3.52 - 3.84)	1.31 (1.25 - 1.36)	0.61 (0.57 - 0.66)	0.54 (0.50 - 0.59)	0.87 (0.82 - 0.92)	1.05 (0.98 - 1.13)
**Branch of industry**								
Manufacturing	0.82 (0.80 - 0.84)	0.89 (0.87 - 0.91)	0.69 (0.67 - 0.71)	0.83 (0.80 - 0.85)	1.01 (0.97 - 1.05)	1.07 (1.03 - 1.11)	0.63 (0.60 - 0.66)	1.11 (1.05 - 1.17)
Service	ref.	ref.	ref.	ref.	ref.	ref.	ref.	ref.
Trade, hotel, restaurant	1.11 (1.08 - 1.13)	0.89 (0.87 - 0.91)	1.04 (1.01 - 1.07)	0.92 (0.89 - 0.95)	0.89 (0.85 - 0.94)	1.10 (1.05 - 1.16)	0.93 (0.89 - 0.98)	0.67 (0.63 - 0.71)
Transport	1.19 (1.14 - 1.23)	1.02 (0.98 - 1.07)	0.91 (0.87 - 0.96)	0.85 (0.81 - 0.90)	1.01 (0.94 - 1.09)	1.11 (1.03 - 1.19)	1.08 (1.00 - 1.16)	0.66 (0.60 - 0.72)
Construction	0.92 (0.88 - 0.96)	1.01 (0.97 - 1.06)	0.67 (0.63 - 0.71)	0.45 (0.42 - 0.49)	0.71 (0.65 - 0.77)	0.98 (0.90 - 1.06)	0.95 (0.88 - 1.04)	0.78 (0.71 - 0.86)
Education, care, nursing, social services	2.03 (1.99 - 2.07)	1.34 (1.31 - 1.37)	1.82 (1.77 - 1.86)	1.19 (1.16 - 1.22)	0.98 (0.94 - 1.03)	1.00 (0.96 - 1.05)	1.73 (1.66 - 1.80)	0.80 (0.76 - 0.83)
**Income (SEK)**								
7920-87 999	0.68 (0.64 - 0.72)	0.62 (0.58 - 0.66)	0.66 (0.62 - 0.71)	0.75 (0.70 - 0.81)	4.25 (3.99 - 4.52)	3.81 (3.55 - 4.10)	1.80 (1.67 - 1.94)	4.50 (4.17 - 4.87)
88 000-175 999	0.90 (0.87 - 0.93)	0.94 (0.91 - 0.98)	1.00 (0.96 - 1.03)	1.17 (1.13 - 1.21)	1.37 (1.29 - 1.46)	1.06 (0.99 - 1.14)	4.36 (4.21 - 4.52)	8.90 (8.57 - 9.25)
176 000-329 999	ref.	ref.	ref.	ref.	ref.	ref.	ref.	ref.
330 000-439 999	0.67 (0.66 - 0.69)	0.89 (0.87 - 0.91)	0.57 (0.55 - 0.58)	0.73 (0.71 - 0.74)	0.81 (0.77 - 0.84)	1.20 (1.15 - 1.25)	0.16 (0.15 - 0.17)	0.12 (0.11 - 0.12)
>440 000	0.39 (0.38 - 0.40)	0.58 (0.57 - 0.60)	0.30 (0.29 - 0.31)	0.45 (0.44 - 0.47)	1.03 (0.99 - 1.07)	1.56 (1.49 - 1.63)	0.05 (0.05 - 0.06)	0.00 (0.00—0.00)
**Number of SA net days in 2012**								
0	ref.	ref.	ref.	ref.	ref.	ref.	ref.	ref.
1-14	3.89 (3.76 - 4.03)	3.59 (3.47 - 3.72)	4.47 (4.29 - 4.66)	2.91 (2.77 - 3.05)	1.60 (1.45 - 1.75)	1.72 (1.56 - 1.89)	5.71 (5.35 - 6.09)	3.36 (3.10 - 3.65)
15-30	4.17 (3.99 - 4.35)	2.80 (2.67 - 2.94)	4.97 (4.73 - 5.23)	4.04 (3.84 - 4.26)	2.10 (1.89 - 2.33)	1.22 (1.06 - 1.41)	5.48 (5.04 - 5.96)	3.27 (2.95 - 3.62)
31-90	4.56 (4.40 - 4.73)	4.10 (3.95 - 4.25)	6.50 (6.25 - 6.76)	4.70 (4.50 - 4.90)	2.96 (2.74 - 3.20)	2.44 (2.23 - 2.67)	8.36 (7.87 - 8.89)	6.78 (6.34 - 7.26)
91-180	5.62 (5.32 - 5.93)	5.42 (5.14 - 5.72)	9.92 (9.40 - 10.46)	7.71 (7.28 - 8.17)	5.92 (5.4 - 6.49)	5.11 (4.60 - 5.67)	22.85 (21.45 - 24.35)	20.67 (19.25 - 22.2)
181-365	6.43 (6.00 - 6.89)	4.73 (4.41 - 5.08)	16.10 (15.16 - 17.10)	10.92 (10.25 - 11.63)	13.88 (12.74 - 15.11)	13.60 (12.42 - 14.89)	47.31 (44.34 - 50.49)	22.75 (21.08 - 24.55)
366*	7.86 (6.45 - 9.57)	2.65 (2.07 - 3.39)	27.08 (23.16 - 31.67)	4.76 (3.78 - 5.98)	36.19 (30.22 - 43.33)	50.60 (42.93 - 59.65)	100.07 (85.89 - 116.59)	61.44 (52.82 - 71.47)
**SA diagnoses in 2012**								
Mental diagnoses	3.31 (3.18 - 3.44)	2.80 (2.69 - 2.91)	11.74 (11.39 - 12.10)	8.27 (8.00 - 8.54)	1.87 (1.71 - 2.05)	2.44 (2.24 - 2.65)	8.76 (8.32 - 9.22)	6.71 (6.32 - 7.12)
Musculoskeletal diagnoses	4.02 (3.86 - 4.19)	3.16 (3.03 - 3.30)	3.06 (2.90 - 3.24)	2.47 (2.33 - 2.62)	1.88 (1.69 - 2.08)	1.54 (1.37 - 1.73)	10.54 (9.98 - 11.13)	6.63 (6.23 - 7.06)
Injury	3.31 (3.12 - 3.52)	3.09 (2.91 - 3.29)	2.35 (2.16 - 2.55)	2.43 (2.24 - 2.64)	1.94 (1.69 - 2.23)	2.66 (2.35 - 3.02)	4.95 (4.49 - 5.45)	3.23 (2.88 - 3.61)
Cancer	4.02 (3.71 - 4.37)	4.04 (3.73 - 4.37)	2.09 (1.83 - 2.38)	1.80 (1.57 - 2.06)	23.29 (21.69 - 25.01)	19.35 (17.86 - 20.97)	4.83 (4.19 - 5.58)	3.04 (2.61 - 3.55)
Circulatory diagnoses	3.14 (2.85 - 3.46)	3.32 (2.97 - 3.71)	1.99 (1.72 - 2.30)	2.30 (1.94 - 2.72)	3.60 (3.06 - 4.25)	6.61 (5.84 - 7.48)	11.96 (10.78 - 13.27)	18.02 (16.09 - 20.19)
Other diagnoses	5.09 (4.92 - 5.28)	3.93 (3.79 - 4.08)	4.29 (4.10 - 4.49)	3.56 (3.40 - 3.73)	2.79 (2.57 - 3.03)	1.25 (1.11 - 1.41)	9.67 (9.18 - 10.19)	7.25 (6.82 - 7.69)

* 2012 was a leap year, thus those individuals were on full-time SA the whole year.

For peer review only

For peer review only

For peer review only - http://bmjopen.bmj.com/site/about/guidelines.xhtml

For peer review only

For peer review only - http://bmjopen.bmj.com/site/about/guidelines.xhtml

Supplementary materials. Salonen, Farrants, Alexanderson. *Sequence analysis of sickness absence and disability pension days in 2012–2018 among privately employed white-collar workers in Sweden: a prospective cohort study*

Supplementary Table 1. The distribution of sociodemographic variables in each cluster of sickness absence (SA) and disability pension (DP) among privately employed white-collar workers

	Cluster 1) low or no SA or DP		Cluster 2) SA due to other diagnoses		Cluster 3) SA due to mental diagnoses		Cluster 4) ineligible for SA and DP		Cluster 5) DP	
	**n**	**%**	**n**	**%**	**n**	**%**	**n**	**%**	**n**	**%**
**Sex**										
Women	512 131	*45.0*	45 859	*68.4*	32 110	*73.2*	7416	*40.9*	11 277	*71.7*
Men	626 646	*55.0*	21 138	*31.6*	11 761	*26.8*	10 734	*59.1*	4444	*28.3*
**Age group**										
16-24	58 138	*5.1*	3864	*5.8*	2032	*4.6*	924	*5.1*	132	*0.8*
25-34	245 816	*21.6*	16 017	*23.9*	10 857	*24.7*	3891	*21.4*	588	*3.7*
35-44	338 445	*29.7*	16 566	*24.7*	15 502	*35.3*	4008	*22.1*	2700	*17.2*
45-54	287 310	*25.2*	19 226	*28.7*	11 588	*26.4*	3819	*21.0*	5993	*38.1*
55-64	102 487	*9.0*	8736	*13.0*	3326	*7.6*	2358	*13.0*	4030	*25.6*
65-67	106 581	*9.4*	2588	*3.9*	566	*1.3*	3150	*17.4*	2278	*14.5*
**Type of living area**										
Large city	589 869	*51.8*	33 849	*50.5*	22 600	*51.5*	10 778	*59.4*	5548	*35.3*
Medium-sized town	347 373	*30.5*	20 336	*30.4*	13 230	*30.2*	4745	*26.1*	5552	*35.3*
Rural or small town	201 535	*17.7*	12 812	*19.1*	8041	*18.3*	2627	*14.5*	4621	*29.4*
**Educational level**										
Primary	54 084	*4.7*	3689	*5.5*	2253	*5.1*	1442	*7.9*	1681	*10.7*
Secondary	461 868	*40.6*	32 312	*48.2*	20 029	*45.7*	6127	*33.8*	9339	*59.4*
Tertiary	622 825	*54.7*	30 996	*46.3*	21 589	*49.2*	10 581	*58.3*	4701	*29.9*
**Country of birth**										
Sweden	1 023 594	*89.9*	58 529	*87.4*	38 952	*88.8*	13 606	*75.0*	14 079	*89.6*
Other Nordic country	23 540	*2.1*	1622	*2.4*	1009	*2.3*	1219	*6.7*	548	*3.5*
Other EU25 country	22 782	*2.0*	1301	*1.9*	866	*2.0*	1219	*6.7*	269	*1.7*
Other countries	68 861	*6.0*	5545	*8.3*	3044	*6.9*	2106	*11.6*	825	*5.2*
**Family composition**										
Couple without children <18 at home	15 3085	*13.4*	8510	*12.7*	3455	*7.9*	3310	*18.2*	3991	*25.4*
Couple with children <18 at home	543 569	*47.7*	28 512	*42.6*	20 232	*46.1*	6087	*33.5*	5034	*32.0*
Single without children <18 at home	371 865	*32.7*	23 388	*34.9*	14 354	*32.7*	7822	*43.1*	5102	*32.5*

For peer review only

Supplementary materials. Salonen, Farrants, Alexanderson. *Sequence analysis of sickness absence and disability pension days in 2012–2018 among privately employed white-collar workers in Sweden: a prospective cohort study*

Single with children <18 at home	70 258	*6.2*	6587	*9.8*	5830	*13.3*	931	*5.1*	1594	*10.1*
**Sector**										
Manufacturing	242 538	*21.3*	10 409	*15.5*	6237	*14.2*	4000	*22.0*	2068	*13.2*
Service	495 208	*43.5*	25 925	*38.7*	18 436	*42.0*	8090	*44.6*	6738	*42.9*
Trade, hotel, restaurant	146 137	*12.8*	8461	*12.6*	5636	*12.8*	2135	*11.8*	1855	*11.8*
Transport	49 760	*4.4*	3091	*4.6*	1686	*3.8*	822	*4.5*	728	*4.6*
Construction	46 164	*4.1*	2228	*3.3*	1147	*2.6*	534	*2.9*	598	*3.8*
Education, care, nursing, social services	158 460	*13.9*	16 861	*25.2*	10 718	*24.4*	2547	*14.0*	3733	*23.7*
**Income (SEK)**										
7920-87 999	304 420	*26.7*	26 911	*40.2*	19 263	*43.9*	4687	*25.8*	6798	*43.2*
88 000-175 999	20 142	*1.8*	1206	*1.8*	842	*1.9*	1317	*7.3*	810	*5.2*
176 000-329 999	66 039	*5.8*	5248	*7.8*	4159	*9.5*	1393	*7.7*	6430	*40.9*
330 000-439 999	318 059	*27.9*	18 897	*28.2*	11 372	*25.9*	3951	*21.8*	1161	*7.4*
>440 000	430 117	*37.8*	14 735	*22.0*	8235	*18.8*	6802	*37.5*	522	*3.3*
**Number of SA net days in 2012**										
0	1 080 290	*94.9*	53 898	*80.4*	32 433	*73.9*	15 296	*84.3*	9801	*62.3*
1 - 14	20 262	*1.8*	3935	*5.9*	2719	*6.2*	458	*2.5*	1054	*6.7*
15 - 30	12 121	*1.1*	2523	*3.8*	1808	*4.1*	360	*2.0*	602	*3.8*
31 - 90	16 252	*1.4*	3703	*5.5*	3178	*7.2*	682	*3.8*	1233	*7.8*
91 - 180	6145	*0.5*	1723	*2.6*	1828	*4.2*	515	*2.8*	1274	*8.1*
181 - 365	3353	*0.3*	1077	*1.6*	1619	*3.7*	659	*3.6*	1438	*9.1*
366*	352	*0.0*	138	*0.2*	286	*0.7*	180	*1.0*	319	*2.0*
**SA diagnoses in 2012**										
Mental diagnoses	16 509	*1.4*	3111	*4.6*	6460	*14.7*	486	*2.7*	1793	*11.4*
Musculoskeletal diagnoses	12 285	*1.1*	2815	*4.2*	1418	*3.2*	364	*2.0*	1620	*10.3*
Injury	6655	*0.6*	1278	*1.9*	597	*1.4*	205	*1.1*	444	*2.8*
Cancer	3058	*0.3*	718	*1.1*	245	*0.6*	1071	*5.9*	202	*1.3*
Circulatory diagnoses	2627	*0.2*	483	*0.7*	201	*0.5*	150	*0.8*	423	*2.7*
Other diagnoses	14 648	*1.3*	4171	*6.2*	2323	*5.3*	637	*3.5*	1760	*11.2*
**Total**	1 138 777	100.0	66 997	100.0	43 871	100.0	18 150	100.0	15 721	100.0

* 2012 was a leap year, thus those indviduals were on SA for full-time all year.

For peer review only

Supplementary materials. Salonen, Farrants, Alexanderson. *Sequence analysis of sickness absence and disability pension days in 2012–2018 among privately employed white-collar workers in Sweden: a prospective cohort study*

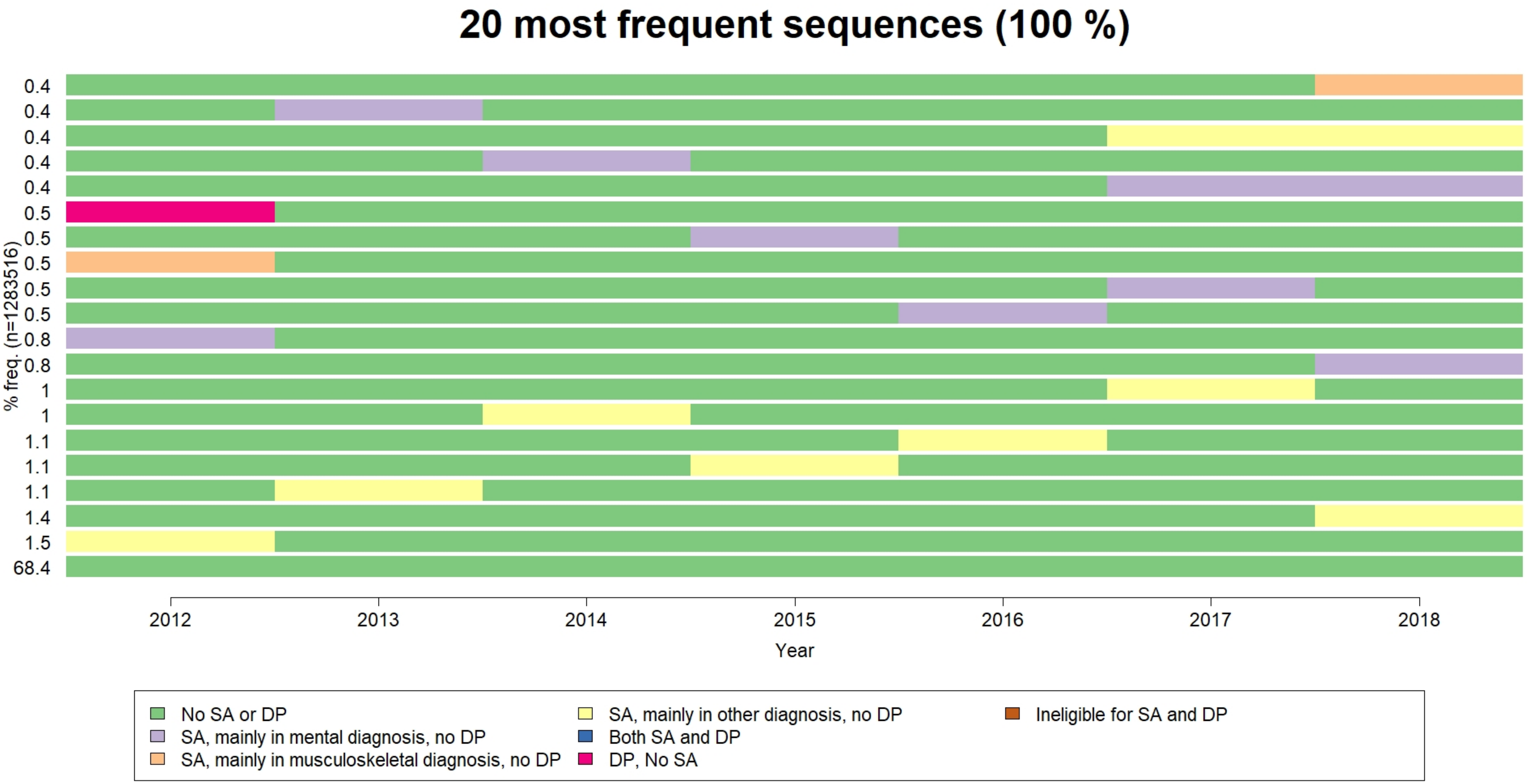

Supplementary Figure 1 Frequency plot for the 20 most frequent sequences of sickness absence (SA) and disability pension (DP) over 7 years among privately employed white-collar workers in Sweden

STROBE Statement—Checklist of items that should be included in reports of ***cohort studies***

	Item No	Recommendation	Page No
**Title and abstract**	1	(*a*) Indicate the study's design with a commonly used term in the title or the abstract	1
		(*b*) Provide in the abstract an informative and balanced summary of what was done and what was found	2
**Introduction**			
Background/rationale	2	Explain the scientific background and rationale for the investigation being reported	4
Objectives	3	State specific objectives, including any prespecified hypotheses	5
**Methods**			
Study design	4	Present key elements of study design early in the paper	5
Setting	5	Describe the setting, locations, and relevant dates, including periods of recruitment, exposure, follow-up, and data collection	5
Participants	6	(*a*) Give the eligibility criteria, and the sources and methods of selection of participants. Describe methods of follow-up	5
		(*b*) For matched studies, give matching criteria and number of exposed and unexposed	NA
Variables	7	Clearly define all outcomes, exposures, predictors, potential confounders, and effect modifiers. Give diagnostic criteria, if applicable	6
Data sources/ measurement	8*	For each variable of interest, give sources of data and details of methods of assessment (measurement). Describe comparability of assessment methods if there is more than one group	6
Bias	9	Describe any efforts to address potential sources of bias	5
Study size	10	Explain how the study size was arrived at	6
Quantitative variables	11	Explain how quantitative variables were handled in the analyses. If applicable, describe which groupings were chosen and why	6
Statistical methods	12	(*a*) Describe all statistical methods, including those used to control for confounding	6-7
		(*b*) Describe any methods used to examine subgroups and interactions	7
		(*c*) Explain how missing data were addressed	NA
		(*d*) If applicable, explain how loss to follow-up was addressed	NA
		(*e*) Describe any sensitivity analyses	NA
**Results**			
Participants	13*	(a) Report numbers of individuals at each stage of study—eg numbers potentially eligible, examined for eligibility, confirmed eligible, included in the study, completing follow-up, and analysed	5
		(b) Give reasons for non-participation at each stage	NA
		(c) Consider use of a flow diagram	NA
Descriptive data	14*	(a) Give characteristics of study participants (eg demographic, clinical, social) and information on exposures and potential confounders	Table 1
		(b) Indicate number of participants with missing data for each variable of interest	Table 1
		(c) Summarise follow-up time (eg, average and total amount)	5
Outcome data	15*	Report numbers of outcome events or summary measures over time	7-8

For peer review only

Main results	16	(*a*) Give unadjusted estimates and, if applicable, confounder-adjusted estimates and their precision (eg, 95% confidence interval). Make clear which confounders were adjusted for and why they were included<br>(*b*) Report category boundaries when continuous variables were categorized<br>(*c*) If relevant, consider translating estimates of relative risk into absolute risk for a meaningful time period	Table 2<br>Table 2<br>NA
Other analyses	17	Report other analyses done—eg analyses of subgroups and interactions, and sensitivity analyses	NA
**Discussion**			
Key results	18	Summarise key results with reference to study objectives	10
Limitations	19	Discuss limitations of the study, taking into account sources of potential bias or imprecision. Discuss both direction and magnitude of any potential bias	11
Interpretation	20	Give a cautious overall interpretation of results considering objectives, limitations, multiplicity of analyses, results from similar studies, and other relevant evidence	11
Generalisability	21	Discuss the generalisability (external validity) of the study results	11
**Other information**			
Funding	22	Give the source of funding and the role of the funders for the present study and, if applicable, for the original study on which the present article is based	12

*Give information separately for exposed and unexposed groups.

Note: An Explanation and Elaboration article discusses each checklist item and gives methodological background and published examples of transparent reporting. The STROBE checklist is best used in conjunction with this article (freely available on the Web sites of PLoS Medicine at http://www.plosmedicine.org/, Annals of Internal Medicine at http://www.annals.org/, and Epidemiology at http://www.epidem.com/). Information on the STROBE Initiative is available at http://www.strobe-statement.org.

For peer review only

For peer review only - http://bmjopen.bmj.com/site/about/guidelines.xhtml

BMJ Open

Sequence analysis of sickness absence and disability pension days in 2012–2018 among privately employed white-collar workers in Sweden: a prospective cohort study

Journal:	*BMJ Open*
Manuscript ID	bmjopen-2023-078066.R2
Article Type:	Original research
Date Submitted by the Author:	23-Nov-2023
Complete List of Authors:	Salonen, Laura; Finnish Institute of Occupational Health<br>Alexanderson, Kristina; Karolinska Institute Division of Insurance Medicine<br>Farrants, Kristin ; Karolinska Institute Division of Insurance Medicine,
**Primary Subject Heading**:	Occupational and environmental medicine
Secondary Subject Heading:	Epidemiology, Public health
Keywords:	EPIDEMIOLOGIC STUDIES, EPIDEMIOLOGY, OCCUPATIONAL & INDUSTRIAL MEDICINE, PUBLIC HEALTH, REGISTRIES

SCHOLARONE™
Manuscripts

I, the Submitting Author has the right to grant and does grant on behalf of all authors of the Work (as defined in the below author licence), an exclusive licence and/or a non-exclusive licence for contributions from authors who are: i) UK Crown employees; ii) where BMJ has agreed a CC-BY licence shall apply, and/or iii) in accordance with the terms applicable for US Federal Government officers or employees acting as part of their official duties; on a worldwide, perpetual, irrevocable, royalty-free basis to BMJ Publishing Group Ltd ("BMJ") its licensees and where the relevant Journal is co-owned by BMJ to the co-owners of the Journal, to publish the Work in this journal and any other BMJ products and to exploit all rights, as set out in our licence.

The Submitting Author accepts and understands that any supply made under these terms is made by BMJ to the Submitting Author unless you are acting as an employee on behalf of your employer or a postgraduate student of an affiliated institution which is paying any applicable article publishing charge ("APC") for Open Access articles. Where the Submitting Author wishes to make the Work available on an Open Access basis (and intends to pay the relevant APC), the terms of reuse of such Open Access shall be governed by a Creative Commons licence – details of these licences and which Creative Commons licence will apply to this Work are set out in our licence referred to above.

Other than as permitted in any relevant BMJ Author's Self Archiving Policies, I confirm this Work has not been accepted for publication elsewhere, is not being considered for publication elsewhere and does not duplicate material already published. I confirm all authors consent to publication of this Work and authorise the granting of this licence.

For peer review only

Sequence analysis of sickness absence and disability pension days in 2012–2018 among privately employed white-collar workers in Sweden: a prospective cohort study

Laura Salonen[1], Kristina Alexanderson[2], & Kristin Farrants[2]

1 Finnish Institute of Occupational Health, Finland

2 Division of Insurance Medicine, Department of Clinical Neuroscience, Karolinska Institutet, Sweden

Corresponding author: Kristin Farrants, kristin.farrants@ki.se

For peer review only

For peer review only - http://bmjopen.bmj.com/site/about/guidelines.xhtml

ABSTRACT

Objective: To explore sequences of sickness absence (SA) and disability pension (DP) days from 2012 through 2018 among privately employed white-collar workers.

Design: A seven-year prospective cohort study using microdata from nationwide registers.

Setting: Sweden.

Participants: All 1,283,516 privately employed white-collar workers in Sweden in 2012 aged 16–67.

Methods: Sequence analysis was used to describe clusters of individuals who followed similar development of SA and DP net days/year, and multinomial logistic regression to analyze associations between sociodemographic variables and belonging to each observed cluster of sequences. Odds ratios (OR) and 95% confidence intervals (CI) were adjusted for baseline sociodemographics.

Results: We identified five clusters of SA and DP sequences: 1) 'low or no SA or DP' (88.7% of the population), 2) 'SA due to other than mental diagnosis' (5.2%), 3) 'SA due to mental diagnosis' (3.4%), 4) 'not eligible for SA or DP' (1.4%), and 5) 'DP' (1.2%). Men, highly educated, born outside Sweden, and high-income earners were more likely to belong to the first and the fourth cluster (ORs range 1.13-4.49). The second, third, and fifth clusters consisted mainly of women, low educated, and low-income (ORs range 1.22-8.90). There were only small differences between branches of industry in adjusted analyses, and many were not significant.

Conclusion: In general, only a few privately employed white-collar workers had SA and even fewer had DP during the seven-year follow-up. The risk of belonging to a cluster characterized by SA or DP varied by sex, levels of education and income, and other sociodemographic factors.

Keywords: sick leave, sequence analysis, private sector

Strengths and limitations of this study:

- Detailed sociodemographic microdata, linked from different population-based registers, about a cohort of all privately employed white-collar workers in Sweden in 2012

For peer review only - http://bmjopen.bmj.com/site/about/guidelines.xhtml

- Long study period (7 years) with no loss to follow-up and no bias from self-reports
- Use of sequence analysis to capture the heterogeneity of the different sickness absence and disability pension patterns over time
- How many and which states to include in the analyses is dependent on researcher judgement and thus can be arbitrary

For peer review only

For peer review only - http://bmjopen.bmj.com/site/about/guidelines.xhtml

INTRODUCTION

Sickness absence (SA) and disability pension (DP) have adverse consequences for individuals, their employers, and welfare states. The development of SA and DP is often a long process and varies with type of occupation and work tasks (1,2). In general, white-collar workers have a lower risk of SA and DP compared to other occupational groups (3–5). Nevertheless, they constitute a large part of the workforce—approximately half in Sweden in 2018 (6), and about half of them are privately employed (7). Thus, work incapacity in this group can impose high costs for employees, employers, and the welfare state. To prevent work incapacity in this population, more knowledge is needed on the determinants and the process of developing long-term work incapacity.

Previous research on SA and DP within specific occupations or occupational groups has mainly focused on so-called high-risk groups, e.g., manual workers and blue-collar workers (8–14), while studies on white-collar workers are scarce. Those conducted are mainly based on small sample sizes (15,16). Most of the research on SA and DP among white-collar employees has focused on publicly employed white-collar employees; for instance, the Whitehall-II studies of British civil servants (17–20). These studies have shown that there are differences in rates of SA among white-collar workers by age, gender, education, occupational status, and other sociodemographic and socioeconomic factors.

Studies on white-collar workers in the *private* sector are even more limited. In general, large-scale studies have demonstrated that SA rates in the private sector are generally lower than in the public sector (21,22). There are several studies on SA and/or DP among private-sector employees, however, hardly any among specifically among white-collar workers, despite how many it concerns. Moreover, the few such studies are mainly based on small, selected populations, have large drop-out rates, and are mainly based on self-reported data (5,23–25). So far, only three large-scale studies on private sector white-collar employees have been published: two Swedish studies (3,26) and a Greek study on private sector employees (also including blue-collar employees) that found a smaller SA rate in the shipyard industry than in other industries (27). The results of the two Swedish studies showed that the risk of SA and DP—and the risk of belonging to an adverse SA/DP trajectory—differed among white-collar workers by age, sex, education, a branch of industry, psychosocial exposures at work, and other sociodemographic factors. Further, none of these studies have accounted for transitions between other labour market states in addition to SA and DP, such as employment and unemployment. More studies using full population data with a longitudinal research design are needed to increase the knowledge base.

Moreover, both SA and DP are complex phenomena affected by many factors. Both increase with age, are lower in people with higher education and non-immigrants, and differ by sex; in most occupations, women have higher SA/DP levels than men, hence it is important to include such factors in studies of future SA/DP (3,28–30).

For peer review only - http://bmjopen.bmj.com/site/about/guidelines.xhtml

Sequence analysis is a good method to study developments over time. Unlike more traditionally used methods, such as event history analysis or growth curve models, sequence analysis can describe the duration and frequency of multiple categorical statuses. This holistic perspective is essential in providing an overview of the future development of SA and DP, and in identifying potential sub-groups within a population who share particular patterns in terms of such SA and DP.

The aim of this study was to identify sequences of white-collar workers in the private sector who follow future similar sequences of SA and DP days/year and second, to analyze the sociodemographic and diagnostic composition of the observed clusters of SA and DP.

METHODS

Data sources and population

We conducted a seven-year prospective population-based cohort study. We used microdata from the following three nationwide Swedish administrative registers, linked at the individual level by personal identity number (PIN; a unique 10-digit number assigned to all Swedish residents) (31):

-The Longitudinal Integration Database for Health Insurance and Labour Market Studies (LISA) held by Statistics Sweden, to identify the study cohort and for information on socio-demographic characteristics at baseline 2012 and regarding being in paid work or not in 2012-2018 (see Variables below) or emigrating in 2013-2018.

-The MicroData for Analysis of the Social Insurance database (MiDAS) held by the Swedish Social Insurance Agency, for information on SA and DP in the years 2012–2018 regarding SA and DP (dates, grades (full- or part-time), and diagnoses), and

-The Cause of Death Register held by the National Board of Health and Welfare for year of death.

The study population consisted of all individuals aged 18–67 years who lived in Sweden on both 31 December 2011 and 31 December 2012, who had an occupational code according to the Swedish Standard for Occupational Classification indicating a white-collar occupation (3), were employed at a private-sector company during 2012, and had an income from work, parental benefits, SA, and/or DP that amounted to at least 75% of the necessary income level to qualify for SA benefits from the Social Insurance Agency (7920 Swedish krona (SEK) in 2012, approx. €910 by the 2012 exchange rate, updated yearly in line with inflation). We excluded unemployed, self-employed, and those who were on full-time DP for the entire year 2012 (n=461). The total study cohort included 1,283,516 individuals.

Public sickness absence insurance in Sweden

In Sweden, all residents aged at least 16 years with an income from work or unemployment benefits who have a reduced work capacity due to morbidity are covered by the national public SA insurance

For peer review only - http://bmjopen.bmj.com/site/about/guidelines.xhtml

(32). A physician's certificate is required after seven days. After an unpaid qualifying day, the employer pays the following 13 SA days, after which SA benefits are paid by the Social Insurance Agency. For unemployed, the Social Insurance Agency pays after the first qualifying day. Thus, we excluded SA spells shorter than 15 days, in order not to introduce bias, since we only had information of SA spells exceeding 14 days for the employed. There was no limitation regarding how long a SA spell could be ongoing for. Residents in Sweden aged 19–64 years, whose work capacity is long-term or permanently reduced can be granted DP from the Social Insurance Agency. SA covers about 80% and DP about 65% of lost income, both up to a certain level. Both SA and DP can be granted for part- or full-time (25%, 50%, 75%, or 100% of ordinary work hours). This means that people can be on partial SA and DP at the same time.

Sociodemographic and work-related variables

We included information on sex, age group, country of birth, educational level, family composition, type of living area, and branch of industry based on the Swedish Standard for Industry Classification (SNI) categorized into the following six groups: manufacturing, services, transport, construction and installation, care and education, or commerce and hospitality. All variables were measured at the baseline year 2012.

Measures on sickness absence and disability pension

We used SA net days/year and DP net days/year as outcomes. Net days were calculated so that partial days of SA or DP were combined, e.g., two days of part-time SA for 50% were summed to one net day, and a similar procedure was used for DP days. The first 14 days of SA spells (>14 days) were counted as being of the same grade as day 15 for the purpose of calculating net days. The number of SA net days in 2012 were categorized as shown in Table 1. The SA diagnoses were categorized into the following seven International Classification of Diseases (ICD-10) groups (33): Cancer (C00-D48), Mental diagnoses (F00-F99 and Z73), Circulatory diseases (I00-I99), Musculoskeletal diagnoses (M00-M99), Pregnancy-related diagnoses (O00-O99), Injuries (S00-T98), and other diagnostic groups (all others, including missing diagnosis (approximately 1% of all spells). In the multinomial logistic regression, pregnancy-related diagnoses were dropped, as no men could have pregnancy-related diagnoses, which made it highly correlated with sex.

In analyses of the yearly states of SA/DP, all diagnoses other than mental and musculoskeletal diseases were combined to form one status. Any DP, regardless of diagnosis, was considered as one group.

Sequence analysis and multinomial regression analysis

For peer review only - http://bmjopen.bmj.com/site/about/guidelines.xhtml

We used sequence analysis to examine different statuses of SA and DP days/year, and the transitions between such statuses. SA and DP status was measured on a yearly basis for each of the seven follow-up years and was coded into one of the following seven statuses:

1) no SA or DP,

2) SA due to mental diagnoses but no DP,

3) SA due to musculoskeletal diagnoses but no DP,

4) SA due to other diagnoses but no DP,

5) both SA and DP,

6) only DP, and

7) ineligible for SA and DP (due to being emigrated, dead, retired, or having no qualifying income from work or work-related benefits).

Individuals who had SA in more than one diagnostic category were assigned to the diagnostic category they had the most days in that year. We illustrated the individual and proportional changes in SA/DP statuses over time with sequence index plots and status proportion plots (34).

We used optimal matching (OM) method to group similar sequences with each other. OM measures the dissimilarities through the changes needed to make two sequences identical (35). In other words, the OM algorithm creates metric distances between two sequences, which can be defined as the minimum combination of replacements, insertion and deletions to transform one sequence to another (36). We used R statistical program version 4.1.0 and packages TraMineR and nnet for the sequence analysis.

We used multinomial regression analysis to analyze how sociodemographic characteristics and branch of industry were associated with each of the obtained clusters, using the first cluster as the reference category. Odds ratios (ORs) with their 95% confidence intervals were reported.

Patient and public involvement

Representatives from the private white-collar sector in Sweden, both for employees and employers (the labour union PTK, the Confederation of Swedish Enterprise, and Alecta) were involved in selecting the research questions through joint meetings throughout the project period, and afterwards in disseminating results.

RESULTS

Characteristics of the study population

For peer review only - http://bmjopen.bmj.com/site/about/guidelines.xhtml

Table 1 shows the characteristics of the study cohort of the 1,283,516 privately employed white-collar workers. There were slightly more men (52.4%) in the cohort. The largest age group was those aged 35-44 years (29.5%), over half lived in a large city (51.5%) and had a tertiary education (53.7%). The majority were born in Sweden (89.7%), and almost half were married or cohabiting and having children below the age of 18 at home (47.3%). The largest group was the service industry (43.1%) and the largest income group was those who earned over 440,000 SEK (around 50,556 EUR according to the average 2012 conversion rate) per year (35.8%). A large majority did not have any SA in 2012: only around 7% had at least some SA spell >14 days. Around 2.2% had SA due to mental diagnoses, 1.4% due to musculoskeletal diagnoses and around 3.8% due to any other diagnoses.

[Table 1. Characteristics of the study population in 2012.]

Clusters of sickness absence and disability pension trajectories

We identified five different groups of sequences, i.e., clusters. Figure 1 shows each of the five clusters, as well as the proportion of individuals in each cluster and the proportion of individuals within the respective clusters in each state during each year. The sociodemographic characteristics of each cluster can be seen in Supplementary Table 1. The first cluster (n=1,138,777, 88.7% of all in the cohort) was the largest one, and almost 95% of individuals in this cluster had no SA or DP days. We called this cluster 'low or no SA or DP'.

Cluster 2 (n=66 997, 5.2%), which was the second largest, was characterized by SA due other than mental diagnosis, including those with mainly musculoskeletal diagnoses (Figure 1). We called this cluster 'SA due to other diagnoses'.

Cluster 3 (n=43 871, 3.4%) consisted mostly of those who had SA mainly due to mental diagnoses (Figure 1). We called this cluster 'SA due to mental diagnoses'.

Cluster 4 (n=18 150, 1.4%) was characterized by individuals who were not eligible for SA or DP since they either died, emigrated, or left the labor force (Figure 1). We called this cluster 'ineligible for SA and DP'.

The smallest cluster, Cluster 5 (n=15 721, 1.2%) was characterized by individuals who had either partial or full-time DP (Figure 1). We called this cluster 'DP'.

To better understand the most common SA and DP sequences, we examined the 20 most frequent sequences (Supplementary Figure 1). Most (68.4%) had no SA or DP during the follow-up. The remaining trajectories largely consisted of sequences where individuals had SA for one year and then returned to no SA or DP. Very few had DP during the follow-up.

For peer review only - http://bmjopen.bmj.com/site/about/guidelines.xhtml

[Figure 1: density plot of sickness absence and disability pension visualizing the proportion of each sickness absence and disability pension status for each cluster over the follow-up]

The associations between individual characteristics and belonging to clusters of sickness absence and disability pension

To study how individual characteristics and SA at baseline were associated with cluster membership, we used multinomial regression analysis. Cluster 1 'low or no SA or DP' was used as the reference category since it was the largest and most homogenous in its sequence content (Table 2). Cluster 1 could be described as consisting of men of younger working-age, who had high levels of education and income, worked in service industry or in manufacturing and had no or only little SA in 2012 (Supplementary Table 1).

In the fully adjusted models, compared to cluster 1 'low or no SA or DP', women (men having an OR of 0.47 (95% CI 0.46-0.47)), over or under 35-44 year old (but not over 64 year old), those with less than tertiary education, who were born outside EU25 countries, living with children, worked in service industry or education, care, nursing, or social service industry, had a medium income, had any SA and especially SA due to cancer or due to 'other diagnoses' were more likely to belong to cluster 2 'SA due to mental diagnoses' (Table 2).

Women (men having an OR of 0.38 (95% CI 0.37-0.39)), 34-44 years old, who had less than tertiary education, who were single living with children, worked in education, care, nursing, or social service industry, had medium low income, had more than 188 SA days in 2012, especially due to mental diagnoses had the highest ORs of belonging to cluster 3 'SA due to mental diagnoses' (Table 2). The second and third clusters could be described as consisting of working-age women, who had less than tertiary education and medium income, who worked in education, care, nursing, or social service industry and had some SA in baseline year, especially due to mental diagnoses in the third cluster (Supplementary Table 1).

The OR for belonging to cluster 4 'ineligible for SA and DP' was the highest in men (OR 1.13; 95% CI 1.10-1.17), 65-67 years old, had primary education, lived without children, were born outside Sweden, had a very low income, who worked in trade, hotel, or restaurant industry or transport industry, had >180 SA days in 2012 and had SA due to circulatory diagnoses (Table 2). The fourth cluster could be described as consisting of men over 64 years old, who had primary education and were born outside Sweden, had low income, and had long-term SA in 2012, especially due to cancer (Supplementary Table 1).

For peer review only - http://bmjopen.bmj.com/site/about/guidelines.xhtml
For peer review only

The OR of belonging to cluster 5 ‘DP’ were higher in women (OR 0.69 (95% CI 0.66-0.72) in men), 45-64 years old, who had less than tertiary education, were born in Sweden, who were single, worked in manufacturing, had low to medium low income, had at least 30 SA days in 2012 and especially those with SA due to circulatory diagnoses (Table 2). This fifth cluster could be described as consisting of older working age women, with low education, working in service industry with low income and long-term SA at baseline (Supplementary Table 1).

[Table 2: associations between sociodemographic factors and work disability clusters]

DISCUSSION

In this large prospective cohort study of all 1.3 million privately employed white-collar workers in Sweden in 2012, we analyzed the development of their future number of SA and DP days/year up through 2018. In general, most of the employees had no SA during the follow-up and DP was even rarer. We found five clusters of future SA and DP trajectories: 1) ‘low or no SA or DP’ (88.7% of all), 2) ‘SA due to other (than mental) diagnosis’ (5.2%), 3) ‘SA due to mental diagnosis’ (3.4%), 4) ‘not eligible for SA or DP’ (1.4%), and 5) ‘DP’ (1.2%). These results suggest that the majority of privately employed white-collar workers were doing well in terms of SA/DP.

We found some differences related to sociodemographic factors in terms of belonging to different sequence clusters. Many of those in cluster 1 ‘low or no SA or DP’ were Swedish-born, 25–54-years old, highly educated, and high-income earning men, who lived in a large city, and were married or cohabiting with children at home. The same sociodemographic characteristics are typically associated with lower risk of SA or DP in longitudinal nationwide studies (28,29).

We also found that female sex, low education, low income, and working in education, care, nursing, or social services were associated with a higher risk of belonging to clusters characterized by at least some SA or DP. Similar results were found in a previous cross-sectional study using the same data with number and prevalence of SA days as outcomes (3), as well as studies on SA and DP among white-collar workers in the retail and wholesale industry (26,37). In general, previous longitudinal population-based studies have consistently found that women, low educated, and low-and income earners (28,29), and those working in healthcare and service industries (22) have a higher risk of SA and/or DP. While these characteristics—low education, low income, and working in the healthcare industry—are usually considered as explanations to why blue-collar workers have a higher risk of SA or DP than white-collar workers (4,38), our results indicate that the same risk factors apply within white-collar employees working in the private sector. More knowledge is warranted regarding potential mechanisms behind this.

For peer review only - http://bmjopen.bmj.com/site/about/guidelines.xhtml

It is understandable that SA due to mental diagnoses constituted an independent cluster since among white-collar workers that is the most common specific diagnostic group of SA and/or DP (1,37,39–41). This cluster was more common among women, 34-44 years old, less than tertiary educated, low-income earners who worked in education, care, nursing and social industry, and had a long SA spell in 2012, which are known risk factors for SA due to mental diagnoses in general (42,43).

The cluster 'ineligible for SA or DP' had relatively many individuals aged ≥55 years, which makes sense since those who left paid work (e.g., through old-age pension) or died during the follow-up belonged to this cluster. There were also many highly educated and high-income earners, who typically are occupationally and geographically mobile, in this cluster. Relatively many of them were born outside Sweden; hence many of them probably emigrated from Sweden. Those who had SA due to cancer in 2012, had higher OR of belonging to this cluster than to any other cluster.

We found that the estimates for associations between branch of industry and cluster attenuated in the adjusted analyses, indicating that differences between the various branches of industry were more related to other factors. The Swedish Social Insurance Agency has found that in Sweden, occupation is more closely associated with SA than branch of industry (44). However, to what extent this is true within the group white-collar workers is unknown and should be further studied.

Strength and limitations

Strengths of this study are the use of a large, population-based cohort, that use of linked microdata from three high-quality nationwide registers without dropouts, the long prospective follow-up, and that all data were administrative, not self-reports with possible bias. Using sequence analysis allowed us to explore specific sub-groups in the development of SA and DP. Other strengths are that all included were covered by the same public SA and DP insurances, and the high employment-frequency in Sweden, that is, the healthy-worker effect did not bias the result much.

Since the study population consisted of privately employed white-collar workers in Sweden, the results cannot directly be generalized to other types of occupational populations or to other countries with other SA/DP systems or employment frequencies. Future studies might choose to explore other, or more specific SA states, regarding number of SA days or part- and full time SA/DP. As this was an observational study, no causal inferences can be drawn from the results.

CONCLUSION

In general, privately employed white-collar workers rarely had SA and even more rarely DP days during the seven-year follow-up. The risk of belonging to a cluster characterized by receiving SA varied by sex, levels of education and income, branch of industry, and other sociodemographic factors.

For peer review only

For peer review only - http://bmjopen.bmj.com/site/about/guidelines.xhtml

Funding: The study was funded by Alecta Insurance, Award/Grant number is not applicable. We utilized data from the REWHARD consortium supported by the Swedish Research Council (grant no. 2017-00624).

Data availability statement: The used data cannot be made publicly available due to privacy regulations. According to the General Data Protection Regulation, the Swedish law SFS 2018:218, the Swedish Data Protection Act, the Swedish Ethical Review Act, and the Public Access to Information and Secrecy Act, these types of sensitive data can only be made available for specific purposes that meets the criteria for access to this type of sensitive and confidential data as determined by a legal review. Professor Kristina Alexanderson (Kristina.alexanderson@ki.se) can be contacted regarding the data.

Ethics statements: The project was approved by the Regional Ethical Review Board in Stockholm, reference numbers 2009/1917-32, 2016/1533-32. In this observational study, based on population-based de-identified register data, informed consent was not applicable. All methods were performed in accordance with the relevant guidelines and regulations.

Patient consent for publication: Not applicable.

Acknowledgements: Not applicable.

Competing Interest: None declared.

Author Contributions: KF and KA planned and designed the study. KF supervised the analyses. LS wrote the first draft of the paper. All authors critically revised the paper for intellectual content. All authors approved the submission of the study.

REFERENCES

1. Helgadóttir B, Narusyte J, Ropponen A, Bergström G, Mather L, Blom V, et al. The role of occupational class on the association between sickness absence and disability pension: A Swedish register-based twin study. Scandinavian Journal of Work, Environment & Health. 2019. 20;45(6):622–30.

2. Salonen L, Blomgren J, Laaksonen M, Niemelä M. Sickness absence as a predictor of disability retirement in different occupational classes: a register-based study of a working-age cohort in Finland in 2007–2014. BMJ Open. 2018. 8(5):e020491.

3. Farrants K, Alexanderson K. Sickness absence among privately employed white-collar workers: A total population study in Sweden. Scand J Public Health. 2021. 49(2):159–67.

For peer review only - http://bmjopen.bmj.com/site/about/guidelines.xhtml

4. Piha K, Laaksonen M, Martikainen P, Rahkonen O, Lahelma E. Interrelationships between education, occupational class, income and sickness absence. The European Journal of Public Health. 2010. 20(3):276–80.

5. Väänänen A, Kalimo R, Toppinen-Tanner S, Mutanen P, Peiró JM, Kivimäki M, et al. Role clarity, fairness, and organizational climate as predictors of sickness absence: A prospective study in the private sector. Scandinavian Journal of Public Health. 2004. 32(6):426–34.

6. Statistics Sweden. Yrkesregistret med yrkesstatistik 2018: Yrkesstrukturen I Sverige [The Swedish Occupational Register with statistics 2018: The occupational structure in Sweden]. Available from: https://www.scb.se/hitta-statistik/statistik-efter-amne/arbetsmarknad/sysselsattning-forvarvsarbete-och-arbetstider/yrkesregistret-med-yrkesstatistik/. Accessed on 31 October 2023.

7. Alecta. Särskild statistik med anledning av coronapandemin. 2023. Available from: https://www.alecta.se/om-alecta/var-syn-pa-saken/var-statistik/coronasiffror/. Accessed on 31 October 2023.

8. Ahola K, Gould R, Virtanen M, Honkonen T, Aromaa A, Lonnqvist J. Occupational burnout as a predictor of disability pension: a population-based cohort study. Occupational and Environmental Medicine. 2009. 66(5):284–90.

9. Alexopoulos EC. Prognostic factors for respiratory sickness absence and return to work among blue collar workers and office personnel. Occupational and Environmental Medicine. 2001. 58(4):246–52.

10. Andersen LL, Fallentin N, Thorsen SV, Holtermann A. Physical workload and risk of long-term sickness absence in the general working population and among blue-collar workers: prospective cohort study with register follow-up. Occupational and Environmental Medicine. 2016. 73(4):246–53.

11. Arndt V. Construction work and risk of occupational disability: a ten year follow up of 14 474 male workers. Occupational and Environmental Medicine. 2005. 62(8):559–66.

12. Haukka E, Kaila-Kangas L, Luukkonen R, Takala EP, Viikari-Juntura E, Leino-Arjas P. Predictors of sickness absence related to musculoskeletal pain: a two-year follow-up study of workers in municipal kitchens. Scandinavian Journal of Work, Environment & Health. 2014. 40(3):278–86.

13. Järvholm B, Stattin M, Robroek SJ, Janlert U, Karlsson B, Burdorf A A. Heavy work and disability pension—a long term follow-up of Swedish construction workers. Scandinavian Journal of Work, Environment & Health. 2014. 40(4):335–42.

For peer review only

14. Rantonen O, Alexanderson K, Pentti J, Kjeldgård L, Hämäläinen J, Mittendorfer-Rutz E, et al. Trends in work disability with mental diagnoses among social workers in Finland and Sweden in 2005–2012. Epidemiology and Psychiatric Sciences. 2016. 1–11.

15. Krantz G, Lundberg U. Workload, work stress, and sickness absence in Swedish male and female white-collar employees. Scandinavian Journal of Social Medicine. 2006. 34(3):238–46.

16. Roelen CAM, Heymans MW, van Rhenen W, Groothoff JW, Twisk JWR, Bültmann U. Fatigue as Prognostic Risk Marker of Mental Sickness Absence in White Collar Employees. Journal of Occupational Rehabilitation. 2013.

17. Feeney A, North F, Head J, Canner R, Marmot M. Socioeconomic and sex differentials in reason for sickness absence from the Whitehall II Study. Occupational and Environmental Medicine. 1998. 55(2):91–8.

18. Head J, Ferrie JE, Alexanderson K, Westerlund H, Vahtera J, Kivimaki M. Diagnosis-specific sickness absence as a predictor of mortality: the Whitehall II prospective cohort study. BMJ. 2008. 337(oct02 2):a1469–a1469.

19. North F, Syme SL, Feeney A, Shipley MJ, Marmot MG. Psychosocial work environment and sickness absence among British civil servants: the Whitehall II study. American Journal of Public Health. 1996. 86:332–40.

20. Stansfeld S, Feeney A, Head J, Canner R, North F, Marmot M. Sickness absence for psychiatric illness: The Whitehall II study. Social Science & Medicine. 1995. 40(2):189–97.

21. Björkenstam E, Helgesson M, Gustafsson K, Virtanen M, Hanson LLM, Mittendorfer-Rutz E. Sickness absence due to common mental disorders in young employees in Sweden: are there differences in occupational class and employment sector? Social Psychiatry and Psychiatric Epidemiology. 2021. 57(5):1097–106.

22. Lund T, Labriola M, Villadsen E. Who is at risk for long-term sickness absence? A prospective cohort study of Danish employees. Work. 2007. 28(3):225–30.

23. Koopmans PC, Roelen CAM, Groothoff JW. Frequent and long-term absence as a risk factor for work disability and job termination among employees in the private sector. Occupational and Environmental Medicine. 2008. 65(7):494–9.

24. Lund T, Kivimäki M, Labriola M, Villadsen E, Christensen KB. Using administrative sickness absence data as a marker of future disability pension: the prospective DREAM study of Danish private sector employees. Occupational and Environmental Medicine. 2008. 65(1):28–31.

For peer review only

For peer review only - http://bmjopen.bmj.com/site/about/guidelines.xhtml

25. Väänänen A, Toppinen-Tanner S, Kalimo R, Mutanen P, Vahtera J, Peiró JM. Job characteristics, physical and psychological symptoms, and social support as antecedents of sickness absence among men and women in the private industrial sector. Social Science & Medicine. 2003. 57(5):807–24.

26. Farrants K, Alexanderson K. Trajectories of sickness absence and disability pension days among 189,321 white-collar workers in the trade and retail industry; a 7-year longitudinal Swedish cohort study. BMC Public Health. 2022. 22(1):1592.

27. Alexopoulos EC, Merekoulias G, Tanagra D, Konstantinou EC, Mikelatou E, Jelastopulu E. Sickness Absence in the Private Sector of Greece: Comparing Shipyard Industry and National Insurance Data. International Journal of Environmental Research and Public Health. 2012. 9(4):1171–81.

28. Allebeck P, Mastekaasa A, Swedish Council on Technology Assessment in Health Care (SBU). Chapter 5. Risk factors for sick leave - general studies. Scandinavian Journal of Public Health. 2004. 32(63_suppl):49–108.

29. Beemsterboer W, Stewart R, Groothoff J, Nijhuis F. A literature review on sick leave determinants (1984-2004). International Journal of Occupational Medicine and Environmental Health 2009. 22(2).

30. De Vries H, Fishta A, Weikert B, Rodriguez Sanchez A, Wegewitz U. Determinants of Sickness Absence and Return to Work Among Employees with Common Mental Disorders: A Scoping Review. J Occup Rehabil. 2018. 28(3):393–417.

31. Ludvigsson JF, Otterblad-Olausson P, Pettersson BU, Ekbom A. The Swedish personal identity number: possibilities and pitfalls in healthcare and medical research. European Journal of Epidemiology. 2009. 24(11):659–67.

32. Swedish Social Insurance Agency. Social Insurance in Figures 2021. Stockholm, Sweden; 2021. Available from: https://statistik.forsakringskassan.se/wps/wcm/connect/11bc72d6-4bbb-4893-8a3b-c9e9eae568f8/social-insurance-in-figures-2021.pdf?MOD=AJPERES&CVID=. Accessed on 31 October 2023.

33. WHO. International Classification of Diseases: Tenth Revision, ICD-10. 2010. Available from: http://apps.who.int/classifications/icd10/browse/2010/en. Accessed on 31 October 2023.

34. Aisenbrey S, Fasang AE. New Life for Old Ideas: The “Second Wave” of Sequence Analysis Bringing the “Course” Back Into the Life Course. Sociological Methods & Research. 2010. 38(3):420–62.

35. Abbott A, Forrest J. Optimal Matching Methods for Historical Sequences. Journal of Interdisciplinary History. 1986. 16(3):471–94.

For peer review only - http://bmjopen.bmj.com/site/about/guidelines.xhtml

36. Abbott A, Tsay A. Sequence Analysis and Optimal Matching Methods in Sociology: Review and Prospect. Sociological Methods & Research. 2000. 29(1):3–33.

37. Farrants K, Alexanderson K. Sickness Absence and Disability Pension in the Trade and Retail Industry: A Prospective Cohort Study of 192,000 White-Collar Workers in Sweden. Journal of Occupational and Environmental Medicine. 2022. 64(11):912–9.

38. Sumanen H, Pietiläinen O, Lahti J, Lahelma E, Rahkonen O. Interrelationships between education, occupational class and income as determinants of sickness absence among young employees in 2002–2007 and 2008–2013. BMC Public Health. 2015. 15(1):332.

39. Karolaakso T, Autio R, Näppilä T, Nurmela K, Pirkola S. Socioeconomic factors in disability retirement due to mental disorders in Finland. European Journal of Public Health. 2020. 30(6):1218–24.

40. Leinonen T, Pietiläinen O, Laaksonen M, Rahkonen O, Lahelma E, Martikainen P. Occupational social class and disability retirement among municipal employees—the contribution of health behaviors and working conditions. Scandinavian Journal of Work, Environment & Health. 2011. 37(6):464–72.

41. Salonen L, Alexanderson K, Rugulies R, Framke E, Niemelä M, Farrants K. Combinations of Job Demands and Job Control and Future Trajectories of Sickness Absence and Disability Pension An 11-year Follow-up of Two Million Employees in Sweden. Journal of Occupational & Environmental Medicine. 2020. 62(10):795–802.

42. Lidwall U, Bill S, Palmer E, Olsson Bohlin C. Mental disorder sick leave in Sweden: A population study. Work. 2018. 59(2):259–72.

43. Foss L, Gravseth HM, Kristensen P, Claussen B, Mehlum IS, Skyberg K. Risk Factors for Long-Term Absence Due To Psychiatric Sickness: A Register-Based 5-Year Follow-Up From the Oslo Health Study. Journal of Occupational & Environmental Medicine. 2010. 52(7):698–705.

44. The Swedish Social Insurance Agency. Sjukfrånvaron på svensk arbetsmarknad: Sjukskrivningar längre än 14 dagar och avslut inom 180 dagar i olika branscher och yrken. [Sickness absence on the Swedish labour market: Sickness absence spells longer than 14 days and ended within 180 days in different branches of industry and occupations]. 2018. Report No.: 12.

For peer review only

For peer review only - http://bmjopen.bmj.com/site/about/guidelines.xhtml

Table 1. Characteristics of the study cohort in 2012.

	Total	
**Sex**	**n**	**%**
Women	598 965	*47.59*
Men	659 755	*52.41*
**Age group**		
16-24	63 788	*5.07*
25-34	271 754	*21.59*
35-44	371 803	*29.54*
45-54	322 900	*25.65*
55-64	117 802	*9.36*
65-67	110 673	*8.79*
**Type of living area**		
Large city	647 868	*51.47*
Medium-sized town	384 746	*30.57*
Rural or small town	226 106	*17.96*
**Educational level**		
Primary	61 256	*4.87*
Secondary	521 351	*41.42*
Tertiary	676 113	*53.71*
**Country of birth**		
Sweden	1 129 201	*89.71*
Other Nordic country	26 478	*2.10*
Other EU25 country	25 010	*1.99*
Other countries	78 031	*6.20*
**Family composition**		
Couple without children <18 at home	167 791	*13.33*
Couple with children <18 at home	595 073	*47.28*
Single without children <18 at home	411 846	*32.72*
Single with children <18 at home	84 010	*6.67*
**Branch of industry**		
Manufacturing	259 419	*20.61*
Service	543 452	*43.17*
Trade, hotel, restaurant	161 308	*12.82*
Transport	54 978	*4.37*
Construction	49 938	*3.97*
Education, care, nursing, social services	189 083	*15.02*
Unknown	542	*0.04*
**Income (SEK)**		
7920-87 999	23 701	*1.88*
88 000-175 999	81 257	*6.46*
176 000-329 999	355 583	*28.25*
330 000-439 999	347 772	*27.63*
>440 000	450 407	*35.78*
**Number of SA net days in 2012 in SA spells >14 gross days**		

For peer review only

For peer review only - http://bmjopen.bmj.com/site/about/guidelines.xhtml

0	1 170 169	*92.96*
1-14	27 895	*2.22*
15-30	17 001	*1.35*
31-90	24 292	*1.93*
91-180	10 885	*0.86*
181-365	7405	*0.59*
366*	1071	*0.09*
**Total**	1 258 720	*100.00*
**SA diagnoses in 2012****		
Mental diagnoses	27 765	*2.21*
Musculoskeletal diagnoses	18 502	*1.44*
Injury	9179	*0.72*
Cancer	5294	*0.41*
Circulatory diagnoses	3884	*0.30*
Pregnancy-related diagnoses	7005	*0.55*
Other diagnoses	23 539	*1.83*

* 2012 was a leap year. ** Individuals could have had several SA spells with different diagnoses.

SA = sickness absence. SEK = Swedish Krona

For peer review only

Table 2. Multinomial regression with five clusters of sickness absence (SA) and disability pension (DP) days/year among privately employed white-collar workers, odds ratios (OR) with their 95% confidence intervals, Cluster 1 'low or no SA or DP' was used as reference group.

	Cluster 2) SA due to other diagnoses		Cluster 3) SA due to mental diagnoses		Cluster 4) ineligible for SA and DP		Cluster 5) DP	
	Crude OR	Adjusted OR	Crude OR	Adjusted OR	Crude OR	Adjusted OR	Crude OR	Adjusted OR
**Sex**								
Women	ref.	ref.	ref.	ref.	ref.	ref.	ref.	ref.
Men	0.38 (0.37 - 0.38)	0.47 (0.46 - 0.47)	0.30 (0.29 - 0.31)	0.38 (0.37 - 0.39)	1.18 (1.15 - 1.22)	1.13 (1.10 - 1.17)	0.32 (0.31 - 0.33)	0.69 (0.66 - 0.72)
**Age group**								
16-24	1.36 (1.31 - 1.41)	1.09 (1.05 - 1.14)	0.76 (0.73 - 0.80)	0.37 (0.35 - 0.39)	1.34 (1.25 - 1.44)	1.54 (1.42 - 1.66)	0.28 (0.24 - 0.34)	0.00 (0.00—0.00)
25-34	1.33 (1.30 - 1.36)	1.30 (1.27 - 1.33)	0.96 (0.94 - 0.99)	0.79 (0.77 - 0.81)	1.34 (1.28 - 1.4)	1.54 (1.47 - 1.61)	0.30 (0.27 - 0.33)	0.10 (0.09 - 0.11)
35-44	ref.	ref.	ref.	ref.	ref.	ref.	ref.	ref.
45-54	1.37 (1.34 - 1.40)	1.42 (1.39 - 1.45)	0.88 (0.86 - 0.90)	0.79 (0.77 - 0.82)	1.12 (1.07 - 1.17)	1.28 (1.22 - 1.34)	2.61 (2.50 - 2.74)	3.34 (3.18 - 3.51)
55-64	1.74 (1.70 - 1.79)	1.73 (1.67 - 1.78)	0.71 (0.68 - 0.74)	0.57 (0.54 - 0.59)	1.94 (1.85 - 2.05)	1.99 (1.88 - 2.10)	4.93 (4.69 - 5.18)	4.29 (4.04 - 4.55)
65-67	0.50 (0.48 - 0.52)	0.45 (0.43 - 0.47)	0.12 (0.11 - 0.13)	0.10 (0.09 - 0.11)	2.50 (2.38 - 2.62)	2.32 (2.20 - 2.45)	2.68 (2.53 - 2.83)	1.26 (1.18 - 1.35)
**Type of living area**								
Large city	ref.	ref.	ref.	ref.	ref.	ref.	ref.	ref.
Medium-sized town	1.02 (1.00 - 1.04)	1.02 (1.00 - 1.04)	0.99 (0.97 - 1.02)	1.06 (1.03 - 1.08)	0.75 (0.72 - 0.77)	0.82 (0.79 - 0.85)	1.70 (1.64 - 1.76)	1.05 (1.01 - 1.10)
Rural or small town	1.11 (1.08 - 1.13)	1.07 (1.05 - 1.09)	1.04 (1.01 - 1.07)	0.97 (0.94 - 0.99)	0.71 (0.68 - 0.74)	0.67 (0.64 - 0.70)	2.44 (2.34 - 2.54)	1.03 (0.99 - 1.08)
**Educational level**								
Primary	1.37 (1.32 - 1.42)	1.66 (1.60 - 1.73)	1.20 (1.15 - 1.26)	1.79 (1.71 - 1.87)	1.57 (1.48 - 1.66)	1.39 (1.32 - 1.48)	4.12 (3.89 - 4.36)	1.68 (1.57 - 1.79)
Secondary	1.41 (1.38 - 1.43)	1.34 (1.32 - 1.36)	1.25 (1.23 - 1.28)	1.22 (1.19 - 1.24)	0.78 (0.76 - 0.81)	0.86 (0.84 - 0.89)	2.68 (2.59 - 2.78)	1.50 (1.44 - 1.56)
Tertiary	ref.	ref.	ref.	ref.	ref.	ref.	ref.	ref.
**Country of birth**								
Sweden	ref.	ref.	ref.	ref.	ref.	ref.	ref.	ref.
Other Nordic country	1.21 (1.15 - 1.27)	1.04 (0.99 - 1.10)	1.13 (1.06 - 1.20)	0.86 (0.80 - 0.92)	3.90 (3.67 - 4.14)	3.25 (3.05 - 3.46)	1.69 (1.55 - 1.85)	0.80 (0.72 - 0.89)
Other EU25 country	1.00 (0.94 - 1.06)	1.07 (1.01 - 1.13)	1.00 (0.93 - 1.07)	1.11 (1.05 - 1.19)	4.03 (3.79 - 4.27)	4.49 (4.25 - 4.76)	0.86 (0.76 - 0.97)	0.31 (0.26 - 0.37)
Other countries	1.41 (1.37 - 1.45)	1.13 (1.10 - 1.17)	1.16 (1.12 - 1.21)	0.87 (0.84 - 0.91)	2.30 (2.20 - 2.41)	2.58 (2.46 - 2.70)	0.87 (0.81 - 0.93)	0.48 (0.44 - 0.52)
**Family composition**								
Couple without children <18 at home	ref.	ref.	ref.	ref.	ref.	ref.	ref.	ref.
Couple with children <18 at home	0.94 (0.92 - 0.97)	0.98 (0.95—1.00)	1.65 (1.59 - 1.71)	0.79 (0.76 - 0.82)	0.52 (0.50 - 0.54)	0.62 (0.59 - 0.65)	0.36 (0.34 - 0.37)	0.71 (0.67 - 0.74)
Single without children <18 at home	1.13 (1.10 - 1.16)	1.19 (1.15 - 1.22)	1.71 (1.65 - 1.78)	0.93 (0.90 - 0.97)	0.97 (0.93 - 1.01)	1.04 (0.99 - 1.08)	0.53 (0.50 - 0.55)	1.22 (1.16 - 1.28)

For peer review only - http://bmjopen.bmj.com/site/about/guidelines.xhtml

Single with children <18 at home	1.69 (1.63 - 1.74)	1.35 (1.30 - 1.40)	3.68 (3.52 - 3.84)	1.31 (1.25 - 1.36)	0.61 (0.57 - 0.66)	0.54 (0.50 - 0.59)	0.87 (0.82 - 0.92)	1.05 (0.98 - 1.13)
**Branch of industry**								
Manufacturing	0.82 (0.80 - 0.84)	0.89 (0.87 - 0.91)	0.69 (0.67 - 0.71)	0.83 (0.80 - 0.85)	1.01 (0.97 - 1.05)	1.07 (1.03 - 1.11)	0.63 (0.60 - 0.66)	1.11 (1.05 - 1.17)
Service	ref.	ref.	ref.	ref.	ref.	ref.	ref.	ref.
Trade, hotel, restaurant	1.11 (1.08 - 1.13)	0.89 (0.87 - 0.91)	1.04 (1.01 - 1.07)	0.92 (0.89 - 0.95)	0.89 (0.85 - 0.94)	1.10 (1.05 - 1.16)	0.93 (0.89 - 0.98)	0.67 (0.63 - 0.71)
Transport	1.19 (1.14 - 1.23)	1.02 (0.98 - 1.07)	0.91 (0.87 - 0.96)	0.85 (0.81 - 0.90)	1.01 (0.94 - 1.09)	1.11 (1.03 - 1.19)	1.08 (1.00 - 1.16)	0.66 (0.60 - 0.72)
Construction	0.92 (0.88 - 0.96)	1.01 (0.97 - 1.06)	0.67 (0.63 - 0.71)	0.45 (0.42 - 0.49)	0.71 (0.65 - 0.77)	0.98 (0.90 - 1.06)	0.95 (0.88 - 1.04)	0.78 (0.71 - 0.86)
Education, care, nursing, social services	2.03 (1.99 - 2.07)	1.34 (1.31 - 1.37)	1.82 (1.77 - 1.86)	1.19 (1.16 - 1.22)	0.98 (0.94 - 1.03)	1.00 (0.96 - 1.05)	1.73 (1.66 - 1.80)	0.80 (0.76 - 0.83)
**Income (SEK)**								
7920-87 999	0.68 (0.64 - 0.72)	0.62 (0.58 - 0.66)	0.66 (0.62 - 0.71)	0.75 (0.70 - 0.81)	4.25 (3.99 - 4.52)	3.81 (3.55 - 4.10)	1.80 (1.67 - 1.94)	4.50 (4.17 - 4.87)
88 000-175 999	0.90 (0.87 - 0.93)	0.94 (0.91 - 0.98)	1.00 (0.96 - 1.03)	1.17 (1.13 - 1.21)	1.37 (1.29 - 1.46)	1.06 (0.99 - 1.14)	4.36 (4.21 - 4.52)	8.90 (8.57 - 9.25)
176 000-329 999	ref.	ref.	ref.	ref.	ref.	ref.	ref.	ref.
330 000-439 999	0.67 (0.66 - 0.69)	0.89 (0.87 - 0.91)	0.57 (0.55 - 0.58)	0.73 (0.71 - 0.74)	0.81 (0.77 - 0.84)	1.20 (1.15 - 1.25)	0.16 (0.15 - 0.17)	0.12 (0.11 - 0.12)
>440 000	0.39 (0.38 - 0.40)	0.58 (0.57 - 0.60)	0.30 (0.29 - 0.31)	0.45 (0.44 - 0.47)	1.03 (0.99 - 1.07)	1.56 (1.49 - 1.63)	0.05 (0.05 - 0.06)	0.00 (0.00—0.00)
**Number of SA net days in 2012**								
0	ref.	ref.	ref.	ref.	ref.	ref.	ref.	ref.
1-14	3.89 (3.76 - 4.03)	3.59 (3.47 - 3.72)	4.47 (4.29 - 4.66)	2.91 (2.77 - 3.05)	1.60 (1.45 - 1.75)	1.72 (1.56 - 1.89)	5.71 (5.35 - 6.09)	3.36 (3.10 - 3.65)
15-30	4.17 (3.99 - 4.35)	2.80 (2.67 - 2.94)	4.97 (4.73 - 5.23)	4.04 (3.84 - 4.26)	2.10 (1.89 - 2.33)	1.22 (1.06 - 1.41)	5.48 (5.04 - 5.96)	3.27 (2.95 - 3.62)
31-90	4.56 (4.40 - 4.73)	4.10 (3.95 - 4.25)	6.50 (6.25 - 6.76)	4.70 (4.50 - 4.90)	2.96 (2.74 - 3.20)	2.44 (2.23 - 2.67)	8.36 (7.87 - 8.89)	6.78 (6.34 - 7.26)
91-180	5.62 (5.32 - 5.93)	5.42 (5.14 - 5.72)	9.92 (9.40 - 10.46)	7.71 (7.28 - 8.17)	5.92 (5.4 - 6.49)	5.11 (4.60 - 5.67)	22.85 (21.45 - 24.35)	20.67 (19.25 - 22.2)
181-365	6.43 (6.00 - 6.89)	4.73 (4.41 - 5.08)	16.10 (15.16 - 17.10)	10.92 (10.25 - 11.63)	13.88 (12.74 - 15.11)	13.60 (12.42 - 14.89)	47.31 (44.34 - 50.49)	22.75 (21.08 - 24.55)
366*	7.86 (6.45 - 9.57)	2.65 (2.07 - 3.39)	27.08 (23.16 - 31.67)	4.76 (3.78 - 5.98)	36.19 (30.22 - 43.33)	50.60 (42.93 - 59.65)	100.07 (85.89 - 116.59)	61.44 (52.82 - 71.47)
**SA diagnoses in 2012**								
Mental diagnoses	3.31 (3.18 - 3.44)	2.80 (2.69 - 2.91)	11.74 (11.39 - 12.10)	8.27 (8.00 - 8.54)	1.87 (1.71 - 2.05)	2.44 (2.24 - 2.65)	8.76 (8.32 - 9.22)	6.71 (6.32 - 7.12)
Musculoskeletal diagnoses	4.02 (3.86 - 4.19)	3.16 (3.03 - 3.30)	3.06 (2.90 - 3.24)	2.47 (2.33 - 2.62)	1.88 (1.69 - 2.08)	1.54 (1.37 - 1.73)	10.54 (9.98 - 11.13)	6.63 (6.23 - 7.06)
Injury	3.31 (3.12 - 3.52)	3.09 (2.91 - 3.29)	2.35 (2.16 - 2.55)	2.43 (2.24 - 2.64)	1.94 (1.69 - 2.23)	2.66 (2.35 - 3.02)	4.95 (4.49 - 5.45)	3.23 (2.88 - 3.61)
Cancer	4.02 (3.71 - 4.37)	4.04 (3.73 - 4.37)	2.09 (1.83 - 2.38)	1.80 (1.57 - 2.06)	23.29 (21.69 - 25.01)	19.35 (17.86 - 20.97)	4.83 (4.19 - 5.58)	3.04 (2.61 - 3.55)
Circulatory diagnoses	3.14 (2.85 - 3.46)	3.32 (2.97 - 3.71)	1.99 (1.72 - 2.30)	2.30 (1.94 - 2.72)	3.60 (3.06 - 4.25)	6.61 (5.84 - 7.48)	11.96 (10.78 - 13.27)	18.02 (16.09 - 20.19)
Other diagnoses	5.09 (4.92 - 5.28)	3.93 (3.79 - 4.08)	4.29 (4.10 - 4.49)	3.56 (3.40 - 3.73)	2.79 (2.57 - 3.03)	1.25 (1.11 - 1.41)	9.67 (9.18 - 10.19)	7.25 (6.82 - 7.69)

* 2012 was a leap year, thus those individuals were on full-time SA the whole year.

For peer review only - http://bmjopen.bmj.com/site/about/guidelines.xhtml

For peer review only

For peer review only

For peer review only - http://bmjopen.bmj.com/site/about/guidelines.xhtml

For peer review only

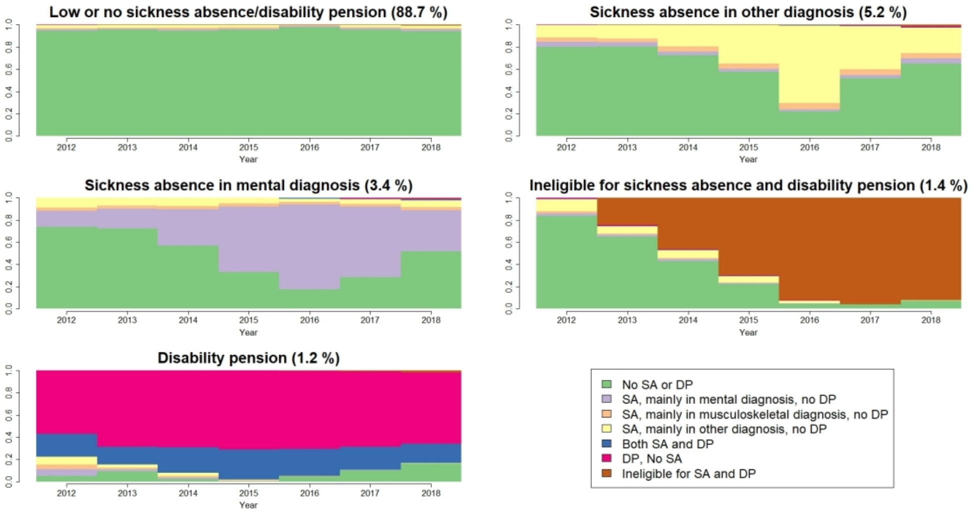

Figure 1: density plot of sickness absence and disability pension visualizing the proportion of each sickness absence and disability pension status for each cluster over the follow-up

677x351mm (72 x 72 DPI)

Supplementary materials. Salonen, Farrants, Alexanderson. *Sequence analysis of sickness absence and disability pension days in 2012–2018 among privately employed white-collar workers in Sweden: a prospective cohort study*

Supplementary Table 1. The distribution of sociodemographic variables in each cluster of sickness absence (SA) and disability pension (DP) among privately employed white-collar workers

	Cluster 1) low or no SA or DP		Cluster 2) SA due to other diagnoses		Cluster 3) SA due to mental diagnoses		Cluster 4) ineligible for SA and DP		Cluster 5) DP	
	**n**	**%**	**n**	**%**	**n**	**%**	**n**	**%**	**n**	**%**
**Sex**										
Women	512 131	*45.0*	45 859	*68.4*	32 110	*73.2*	7416	*40.9*	11 277	*71.7*
Men	626 646	*55.0*	21 138	*31.6*	11 761	*26.8*	10 734	*59.1*	4444	*28.3*
**Age group**										
16-24	58 138	*5.1*	3864	*5.8*	2032	*4.6*	924	*5.1*	132	*0.8*
25-34	245 816	*21.6*	16 017	*23.9*	10 857	*24.7*	3891	*21.4*	588	*3.7*
35-44	338 445	*29.7*	16 566	*24.7*	15 502	*35.3*	4008	*22.1*	2700	*17.2*
45-54	287 310	*25.2*	19 226	*28.7*	11 588	*26.4*	3819	*21.0*	5993	*38.1*
55-64	102 487	*9.0*	8736	*13.0*	3326	*7.6*	2358	*13.0*	4030	*25.6*
65-67	106 581	*9.4*	2588	*3.9*	566	*1.3*	3150	*17.4*	2278	*14.5*
**Type of living area**										
Large city	589 869	*51.8*	33 849	*50.5*	22 600	*51.5*	10 778	*59.4*	5548	*35.3*
Medium-sized town	347 373	*30.5*	20 336	*30.4*	13 230	*30.2*	4745	*26.1*	5552	*35.3*
Rural or small town	201 535	*17.7*	12 812	*19.1*	8041	*18.3*	2627	*14.5*	4621	*29.4*
**Educational level**										
Primary	54 084	*4.7*	3689	*5.5*	2253	*5.1*	1442	*7.9*	1681	*10.7*
Secondary	461 868	*40.6*	32 312	*48.2*	20 029	*45.7*	6127	*33.8*	9339	*59.4*
Tertiary	622 825	*54.7*	30 996	*46.3*	21 589	*49.2*	10 581	*58.3*	4701	*29.9*
**Country of birth**										
Sweden	1 023 594	*89.9*	58 529	*87.4*	38 952	*88.8*	13 606	*75.0*	14 079	*89.6*
Other Nordic country	23 540	*2.1*	1622	*2.4*	1009	*2.3*	1219	*6.7*	548	*3.5*
Other EU25 country	22 782	*2.0*	1301	*1.9*	866	*2.0*	1219	*6.7*	269	*1.7*
Other countries	68 861	*6.0*	5545	*8.3*	3044	*6.9*	2106	*11.6*	825	*5.2*
**Family composition**										
Couple without children <18 at home	15 3085	*13.4*	8510	*12.7*	3455	*7.9*	3310	*18.2*	3991	*25.4*
Couple with children <18 at home	543 569	*47.7*	28 512	*42.6*	20 232	*46.1*	6087	*33.5*	5034	*32.0*
Single without children <18 at home	371 865	*32.7*	23 388	*34.9*	14 354	*32.7*	7822	*43.1*	5102	*32.5*

For peer review only

Supplementary materials. Salonen, Farrants, Alexanderson. *Sequence analysis of sickness absence and disability pension days in 2012–2018 among privately employed white-collar workers in Sweden: a prospective cohort study*

Single with children <18 at home	70 258	*6.2*	6587	*9.8*	5830	*13.3*	931	*5.1*	1594	*10.1*
**Sector**										
Manufacturing	242 538	*21.3*	10 409	*15.5*	6237	*14.2*	4000	*22.0*	2068	*13.2*
Service	495 208	*43.5*	25 925	*38.7*	18 436	*42.0*	8090	*44.6*	6738	*42.9*
Trade, hotel, restaurant	146 137	*12.8*	8461	*12.6*	5636	*12.8*	2135	*11.8*	1855	*11.8*
Transport	49 760	*4.4*	3091	*4.6*	1686	*3.8*	822	*4.5*	728	*4.6*
Construction	46 164	*4.1*	2228	*3.3*	1147	*2.6*	534	*2.9*	598	*3.8*
Education, care, nursing, social services	158 460	*13.9*	16 861	*25.2*	10 718	*24.4*	2547	*14.0*	3733	*23.7*
**Income (SEK)**										
7920-87 999	304 420	*26.7*	26 911	*40.2*	19 263	*43.9*	4687	*25.8*	6798	*43.2*
88 000-175 999	20 142	*1.8*	1206	*1.8*	842	*1.9*	1317	*7.3*	810	*5.2*
176 000-329 999	66 039	*5.8*	5248	*7.8*	4159	*9.5*	1393	*7.7*	6430	*40.9*
330 000-439 999	318 059	*27.9*	18 897	*28.2*	11 372	*25.9*	3951	*21.8*	1161	*7.4*
>440 000	430 117	*37.8*	14 735	*22.0*	8235	*18.8*	6802	*37.5*	522	*3.3*
**Number of SA net days in 2012**										
0	1 080 290	*94.9*	53 898	*80.4*	32 433	*73.9*	15 296	*84.3*	9801	*62.3*
1 - 14	20 262	*1.8*	3935	*5.9*	2719	*6.2*	458	*2.5*	1054	*6.7*
15 - 30	12 121	*1.1*	2523	*3.8*	1808	*4.1*	360	*2.0*	602	*3.8*
31 - 90	16 252	*1.4*	3703	*5.5*	3178	*7.2*	682	*3.8*	1233	*7.8*
91 - 180	6145	*0.5*	1723	*2.6*	1828	*4.2*	515	*2.8*	1274	*8.1*
181 - 365	3353	*0.3*	1077	*1.6*	1619	*3.7*	659	*3.6*	1438	*9.1*
366*	352	*0.0*	138	*0.2*	286	*0.7*	180	*1.0*	319	*2.0*
**SA diagnoses in 2012**										
Mental diagnoses	16 509	*1.4*	3111	*4.6*	6460	*14.7*	486	*2.7*	1793	*11.4*
Musculoskeletal diagnoses	12 285	*1.1*	2815	*4.2*	1418	*3.2*	364	*2.0*	1620	*10.3*
Injury	6655	*0.6*	1278	*1.9*	597	*1.4*	205	*1.1*	444	*2.8*
Cancer	3058	*0.3*	718	*1.1*	245	*0.6*	1071	*5.9*	202	*1.3*
Circulatory diagnoses	2627	*0.2*	483	*0.7*	201	*0.5*	150	*0.8*	423	*2.7*
Other diagnoses	14 648	*1.3*	4171	*6.2*	2323	*5.3*	637	*3.5*	1760	*11.2*
**Total**	1 138 777	100.0	66 997	100.0	43 871	100.0	18 150	100.0	15 721	100.0

* 2012 was a leap year, thus those indviduals were on SA for full-time all year.

For peer review only

Supplementary materials. Salonen, Farrants, Alexanderson. *Sequence analysis of sickness absence and disability pension days in 2012–2018 among privately employed white-collar workers in Sweden: a prospective cohort study*

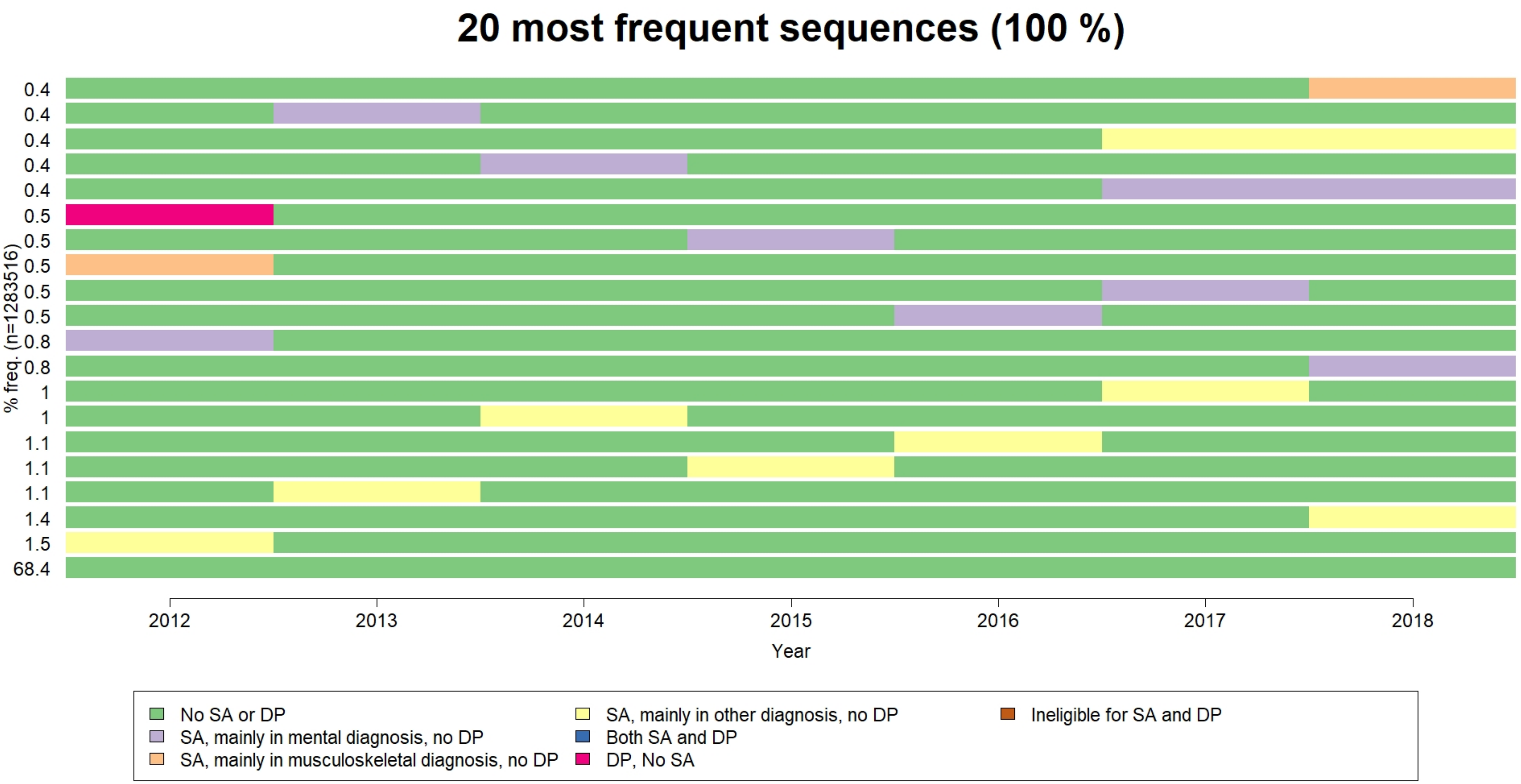

Supplementary Figure 1 Frequency plot for the 20 most frequent sequences of sickness absence (SA) and disability pension (DP) over 7 years among privately employed white-collar workers in Sweden

STROBE Statement—Checklist of items that should be included in reports of ***cohort studies***

	Item No	Recommendation	Page No
**Title and abstract**	1	(*a*) Indicate the study's design with a commonly used term in the title or the abstract	1
		(*b*) Provide in the abstract an informative and balanced summary of what was done and what was found	2
**Introduction**			
Background/rationale	2	Explain the scientific background and rationale for the investigation being reported	4
Objectives	3	State specific objectives, including any prespecified hypotheses	5
**Methods**			
Study design	4	Present key elements of study design early in the paper	5
Setting	5	Describe the setting, locations, and relevant dates, including periods of recruitment, exposure, follow-up, and data collection	5
Participants	6	(*a*) Give the eligibility criteria, and the sources and methods of selection of participants. Describe methods of follow-up	5
		(*b*) For matched studies, give matching criteria and number of exposed and unexposed	NA
Variables	7	Clearly define all outcomes, exposures, predictors, potential confounders, and effect modifiers. Give diagnostic criteria, if applicable	6
Data sources/ measurement	8*	For each variable of interest, give sources of data and details of methods of assessment (measurement). Describe comparability of assessment methods if there is more than one group	6
Bias	9	Describe any efforts to address potential sources of bias	5
Study size	10	Explain how the study size was arrived at	6
Quantitative variables	11	Explain how quantitative variables were handled in the analyses. If applicable, describe which groupings were chosen and why	6
Statistical methods	12	(*a*) Describe all statistical methods, including those used to control for confounding	6-7
		(*b*) Describe any methods used to examine subgroups and interactions	7
		(*c*) Explain how missing data were addressed	NA
		(*d*) If applicable, explain how loss to follow-up was addressed	NA
		(*e*) Describe any sensitivity analyses	NA
**Results**			
Participants	13*	(a) Report numbers of individuals at each stage of study—eg numbers potentially eligible, examined for eligibility, confirmed eligible, included in the study, completing follow-up, and analysed	5
		(b) Give reasons for non-participation at each stage	NA
		(c) Consider use of a flow diagram	NA
Descriptive data	14*	(a) Give characteristics of study participants (eg demographic, clinical, social) and information on exposures and potential confounders	Table 1
		(b) Indicate number of participants with missing data for each variable of interest	Table 1
		(c) Summarise follow-up time (eg, average and total amount)	5
Outcome data	15*	Report numbers of outcome events or summary measures over time	7-8

For peer review only - http://bmjopen.bmj.com/site/about/guidelines.xhtml

Main results	16	(*a*) Give unadjusted estimates and, if applicable, confounder-adjusted estimates and their precision (eg, 95% confidence interval). Make clear which confounders were adjusted for and why they were included<br>(*b*) Report category boundaries when continuous variables were categorized<br>(*c*) If relevant, consider translating estimates of relative risk into absolute risk for a meaningful time period	Table 2<br>Table 2<br>NA
Other analyses	17	Report other analyses done—eg analyses of subgroups and interactions, and sensitivity analyses	NA
**Discussion**			
Key results	18	Summarise key results with reference to study objectives	10
Limitations	19	Discuss limitations of the study, taking into account sources of potential bias or imprecision. Discuss both direction and magnitude of any potential bias	11
Interpretation	20	Give a cautious overall interpretation of results considering objectives, limitations, multiplicity of analyses, results from similar studies, and other relevant evidence	11
Generalisability	21	Discuss the generalisability (external validity) of the study results	11
**Other information**			
Funding	22	Give the source of funding and the role of the funders for the present study and, if applicable, for the original study on which the present article is based	12

*Give information separately for exposed and unexposed groups.

Note: An Explanation and Elaboration article discusses each checklist item and gives methodological background and published examples of transparent reporting. The STROBE checklist is best used in conjunction with this article (freely available on the Web sites of PLoS Medicine at http://www.plosmedicine.org/, Annals of Internal Medicine at http://www.annals.org/, and Epidemiology at http://www.epidem.com/). Information on the STROBE Initiative is available at http://www.strobe-statement.org.

For peer review only
